# Supplementary material for: Changes in quality of life (QoL) and other patient-reported outcome measures (PROMs) in living-donor and deceased-donor kidney transplant recipients and those awaiting transplantation in the UK ATTOM programme: a longitudinal cohort questionnaire survey with additional qualitative interviews
Source: BMJ Open. 2021 Apr 8;11(4):e047263. doi: 10.1136/bmjopen-2020-047263 (PMC8098938; doi:10.1136/bmjopen-2020-047263)
Supplement: Supplementary data [file bmjopen-2020-047263supp001.pdf]

Supplementary Data

Supplementary Data 1. Interview schedule for semi-structured interviews with LD and DD recipients.

The interview schedule included prompt questions, but each schedule was tailored to the individual and their questionnaire responses to the Renal Dependent Quality of Life (RDQoL) Questionnaire and the Renal Treatment Satisfaction Questionnaires (RTSQs).

HISTORY
Can you describe the process that you went through before you received a kidney transplant?

All patients:
- When did you first start having problems with your kidneys?
- How did your kidney problems develop?
- Were you placed on the transplant list? When?

For patients who received a DD transplant:
- How long were you waiting for a kidney?
- How did you feel about the time you spent waiting?
- Was the option of a living donor mentioned to you?

For patients who received an LD transplant:
- Who first suggested a living donor to you?
- How did you find information about living donation?
    - How satisfied were you with the information you were given?
- How did you bring the topic up with your family or friends?
    - Did you feel able to talk about it?
- Who donated a kidney to you?
    - How are they doing now?

QUALITY OF LIFE
Ok, so now I'd like to move on and talk to you about your quality of life.
In the questionnaires that you completed recently, you report that overall, your quality of life is excellent / very good / good / neither good nor bad / bad / very bad / extremely bad.

You said that your renal condition does / does not have an impact on your quality of life.
Can you give me an example of how (aspect of life) has been affected?
Other aspect(s) of your life that appeared to be most impacted by your renal condition was/were …………
Can you tell me in what ways your renal condition impacts on ………………….?

For patients with QoL changes over time:
In the questionnaires, you report that your quality of life has changed since you received a kidney transplant. Can you tell me why that is?

TREATMENT SATISFACTION
In terms of your treatment satisfaction, I can see from your questionnaires that you are very satisfied / satisfied / a little dissatisfied / dissatisfied / very dissatisfied with your current treatment. Can you tell me why?
I can see from your questionnaires that the aspect(s) of your treatment that you are most satisfied with was/were ………… Can you tell me why?
You were least satisfied with ………….. Can you tell me why?

Your treatment satisfaction did / did not change over time from 3 months after recruitment / transplant to 12 months after recruitment / transplant. Can you tell me why?

How might your satisfaction with treatment be improved?

Supplementary Data 2. Table 1. Summary of outcome measures

		No. of items	Range
Quality of life (ADDQoL and RDQoL)			
Generic QoL item	A single item assessing present QoL.	1	+3 (excellent) to -3 (extremely bad)
Renal Dependent Quality of Life (RDQoL)	Weighted-impact scores calculated by multiplying (a) impact of renal condition on aspect of life with (b) importance rating of aspect of life and summing all applicable items to give an average weighted-impact (AWI) score reflecting the impact of the renal condition on QoL.	21 (6 N/A options) *	-9 (most negative weighted impact) to +3 (most positive weighted impact)
Well-being (W-BQ12)			
Total well-being	12 items scored on 4-point scales from 3 (all the time) to 0 (not at all). Overall well-being score calculated by combining the scores from the 3 subscales (negative well-being, energy, positive well-being). Higher scores indicate greater well-being during the past few weeks.	12	36-0
Health status (EQ-5D-5L)			
Utility values	5 dimensions of health (today) rated on 5 levels converted to population preference values using the new value set for England.[18] The higher the score, the greater the preference utility.	5	+1.00 (perfect health) to -0.281 (worse than death)
EQ-VAS ratings	Self-reported health (today) on visual analogue scale from 100 (best health you can imagine) to 0 (worst health you can imagine).	1	100-0
Treatment satisfaction (RTSQs/c)			
Renal Treatment Satisfaction Questionnaire (RTSQ) status version (RTSQs)	Items assess satisfaction with current renal treatment on 7-point scales from 6 (e.g. very satisfied) to 0 (e.g. very dissatisfied). Items are summed to give a total satisfaction score. The higher the score, the greater the satisfaction with renal treatment.	13	78-0
RTSQ change version (RTSQc)	Items assess satisfaction with current renal treatment compared with satisfaction with previous renal treatment, from +3 (e.g. much more satisfied now) to -3 (e.g. much less satisfied now). A score of 0 indicates no change in satisfaction. Items are summed to give a total change in satisfaction score.	13	+39 (much more satisfied now) to 39 (much less satisfied now)

*after preliminary psychometric analyses, only 17 of the items (including 3 with not applicable options) were included in the Average Weighted Impact (AWI) score (see Appendix 2). The range of AWI scores remains the same.

Supplementary Data 3. Psychometric properties of the RDQoL and RTSQs/c

The Renal Dependent Quality of Life (RDQoL) questionnaire was designed to examine the impact of renal conditions on QoL.[1] Following the template of the Audit of Diabetes-Dependent QoL (ADDQoL),[2,3] the RDQoL contains two overview items and a series of domain-specific items covering different aspects of life commonly impacted by a renal condition and its treatment. Each domain-specific item is worded *'If did not have a renal condition my…would be'*, followed by a rating scale for the impact of renal condition on that domain of life from -3 (e.g. very much better) to +1 (e.g. worse), and a rating of the importance of the domain for QoL from very important (+3) to not at all important (0). The impact and importance scores are multiplied to give a weighted impact score for each item, ranging from -9 (most negative weighted impact on QoL) to +3 (most positive weighted impact). Some of the items include preliminary questions to determine if the domain is applicable to the individual (e.g. work). Each of the applicable weighted impact scores are summed and the total is divided by the number of applicable items to give an average weighted impact (AWI) score, also ranging from -9 to +3.

The Renal Treatment Satisfaction Questionnaire (RTSQ) is a measure of satisfaction with renal treatment. Following the templates of the Diabetes Treatment Satisfaction Questionnaire (DTSQ) [4-6] there are two versions: the status version (RTSQs)[7] and the change version (RTSQc). The RTSQs assesses level of satisfaction with current renal treatment. The 13 items are summed to give an overall treatment satisfaction score, with higher scores indicating greater satisfaction (range=78-0). The RTSQc was developed to overcome the ceiling effects sometimes found with the status version. Using similar item stems as the RTSQs but different response options, the RTSQc asks participants to compare satisfaction with their current treatment with satisfaction with their previous treatment (range +39 e.g. much more satisfied to -39 much less satisfied). Barendse et al[7] conducted analyses on the original 11-item RTSQs, and found that it had acceptable reliability. Although a forced one-factor structure was acceptable for all patients, a two-factor structure was found for transplant recipients, and a three-factor structure for non-transplant patients.[6] The 13-item version of the RTSQs has additional

items related to satisfaction with the side effects of treatment, and satisfaction with the demands of treatment. No psychometric analyses have been published using the 13-item version, and none have been conducted with the RTSQc. Analyses, therefore, were conducted to test the psychometric properties of these measures in this sample.

In ATTOM, the number of participants in each group recruited were as follows; 104 DD recipients, 94 LD recipients, 98 patients wait-listed for a transplant, 136 participants receiving PD or HD, and 16 patients whose transplant failed during ATTOM. Participants were invited to complete the measures at 12m post-transplant or post-recruitment. There was a possibility that differences between the groups might create artefactual correlations in the combined sample, if the subgroup mean scores differed systematically across items. The possibility of artefactual correlations was therefore investigated by converting the scores on each measure to standardised z scores within each subgroup before combining the sub-sets of scores and conducting the factor analyses. Such standardisation renders group means identical and thereby removes the possibility of correlations caused by subgroup differences. Forced one-factor solutions on the raw scores, and then on the Z scores, produced two sets of factor loadings that were then compared using correlation and regression cross-check analyses.

The crosscheck analyses tested whether the transplant groups (DD and LD recipients) and non-transplant groups could be combined for these analyses. The non-transplant groups included dialysis and pre-dialysis patients including those wait-listed for a kidney transplant, and those whose transplants failed during ATTOM. For ease of analysis, these were grouped into those on HD, PD, or pre-dialysis. Participants with missing data on any items were excluded from the analyses. The transplant groups could be combined for analyses for the RDQoL, RTSQs and RTSQc. The non-transplant groups (on either HD, PD or pre-dialysis) could be combined for the RDQoL and RTSQs, whilst only the HD and PD groups could be combined for the RTSQc. Those not yet on dialysis formed a much smaller sub-group (n=15), so were not included in separate analyses.

RDQoL

Factor structure

Analyses were conducted to confirm the factor structure of the RDQoL, RTSQs, and RTSQc. Parallel analyses were conducted to identify how many factors are acceptable to retain in the structure of each measure. Principal Axis Factoring (PAF) was then used to examine the variance and the item loadings unto these factors. A Kaiser-Meyer-Olkin Measure of Sampling Adequacy (KMO) value of 0.60 or above, and correlation coefficients of 0.30 and above between each item indicates that the analysis is appropriate and the items should be retained. An item loading above 0·40 is considered acceptable. See Tables 5 and 6 for a summary of the descriptive statistics, reliability analysis, and factor structure of the RDQoL for transplant and non-transplant groups. Parallel analysis indicated that a one-factor solution was acceptable for both the transplant and non-transplant groups.

Transplant (LD and DD) group: Principal Axis Factoring (PAF) with a forced one-factor solution led to 48·04% of the variance being explained, with item loadings from 0.266 to 0.855 (KMO=0.92, $\chi^2_{(210)}$=1738.60, p<0.001). The spiritual/religious life item had the lowest item loading of 0.266, and correlated with only one other item above 0.30, so was removed, and the analysis re-run. This led to 49·97% of the variance being explained, with more acceptable loadings ranging from 0.417 to 0.856 (KMO=0.93, $\chi^2_{(210)}$=1715.451, p<0.001). Item 19 (fuss/worry), although displaying an acceptable factor loading (0.417), correlated with few other items above 0.30.

Non-transplant group: Principal Axis Factoring with a forced one-factor solution led to 38.95% of the variance being explained, with item loadings from 0.250 to 0.730 (KMO=0.92, $\chi^2_{(210)}$=1431.71, p<0.001). Item 2 (work, 0.250), item 4 (holidays 0.331), and item 14 (religious/spiritual life 0.349) had item loadings below 0.40. Work was only correlated above 0.30 with one other item, whilst holidays and spiritual/religious life were each correlated with four other items above 0.30. These items were removed, and the analysis re-run. This led to 43.70% of the variance being explained, with item loadings from 0.552 to 0.740 (KMO=0.93, $\chi^2_{(153)}$=1308.43, p<0.001).

Supplementary Table 2. Descriptive statistics, reliability analysis, and forced one-factor solution Principal Axis Factoring (PAF) on the RDQoL weighted impact scores in the transplant recipients (with deceased and living donor kidneys; $n = 137$).

	Missing N	**Not applicable**	**Mean**	**Median**	***SD***	**Corrected Item-total correlation**	**Cronbach's alpha if item deleted***	**PAF Item Loadings**
**1. Leisure activities**	1	-	-2.72	-2.00	2.68	0.717	0.940	0.741
**2. Work**	3	37	-2.46	0.00	3.18	0.610	0.942	0.617
**3. Local/long distance journeys**	1	-	-1.79	-1.00	2.41	0.612	0.942	0.630
**4. Holidays**	3	9	-2.83	-2.00	2.96	0.716	0.940	0.735
**5. Physical ability**	2	-	-3.06	-3.00	2.80	0.783	0.939	0.811
**6. Family**	1	1	-2.42	-1.50	2.89	0.714	0.940	0.743
**7. Friendships/social life**	0	-	-2.34	-1.50	2.88	0.789	0.939	0.815
**8. Close personal relationship**	3	14	-2.37	0.00	3.10	0.713	0.940	0.746
**9. Sex life**	2	16	-2.61	-2.00	2.99	0.723	0.940	0.751
**10. Physical appearance**	3	-	-2.37	-2.00	2.60	0.540	0.943	0.561
**11. Self-confidence**	1	-	-2.25	-2.00	2.74	0.783	0.939	0.813
**12. Motivation**	4	-	-2.26	-2.00	2.86	0.825	0.938	0.855
**13. People react to me**	1	-	-0.84	0.00	1.78	0.607	0.942	0.633
**14. Spiritual/religious life**	0	75	-0.31	0.00	1.75	-	-	0.266
**15. Feelings about the future**	0	-	-3.42	-2.00	3.34	0.576	0.943	0.588
**16. Financial situation**	1	-	-2.61	-2.00	3.23	0.686	0.941	0.696
**17. Living conditions**	1	-	-1.41	0.00	2.40	0.659	0.941	0.685
**18. Dependence on others**	2	-	-2.40	-1.00	3.06	0.543	0.943	0.548
**19. People fuss/worry**	0	-	-3.20	-2.00	3.05	0.417	0.945	0.427
**20. Freedom to eat**	0	-	-1.76	-1.00	2.45	0.614	0.942	0.635
**21. Freedom to drink**	0	-	-1.69	-1.00	2.51	0.603	0.942	0.614

*Total Cronbach's alpha = 0·944.

Reliability analyses

Cronbach's alpha (α) was calculated as a test of internal consistency; how closely related the items in the measure are as a group. Scores range from 0 to 1, with a score above 0.70 commonly being used as a threshold for acceptable reliability for group comparison with 0.80 providing a more stringent threshold. Analyses are repeated, removing one item at a time to calculate how many missing items a measure can tolerate, whilst still retaining an acceptable alpha coefficient. Even if the alpha

coefficient is above the threshold, no more than 50% of the items in a scale or subscale are allowed to be missing, because to do so is likely to produce too much distortion of the construct measured by the scale.

Transplant (LD and DD) group: Analyses conducted using all items except item 14 (spiritual/religious life) revealed that the RDQoL has excellent reliability (Cronbach's α=0.944). The removal of item 19 (fuss or worry) would improve the alpha coefficient from 0.944 to 0.945. The fuss and worry item also had the lowest item-total correlations (0·417), and the lowest item loadings (0.427), suggesting that its removal could improve the factor structure and reliability of the RDQoL.

Non-transplant group: Reliability analyses revealed that, not including item 2 (work) and 14 (spiritual/religious life), the RDQoL has excellent reliability (Cronbach's α=0.920; Table 6). The removal of item 4 (holidays) would improve the alpha coefficient from 0.920 to 0.922. The item had a low factor loading (0.331) and low item-total correlation (r = 0.326).

Not including those items that have a non-applicable option, reliability analyses indicated that the questionnaire (14 items) could tolerate up to seven missing items and still retain a Cronbach's α coefficient above 0.70 for the transplant group (α = 0.786) and non-transplant group (α = 0.792).

Supplementary Table 3. Descriptive statistics, reliability analysis, and forced one-factor solution Principal Axis Factoring (PAF) on the RDQoL weighted impact scores in the non-transplant groups (*n*= 172).

	Missing N	Not applicable	Mean	Median	*SD*	Corrected Item-total correlation	Cronbach's alpha if item deleted*	PAF Item Loadings
**1. Leisure activities**	3	-	-4.84	-4.00	2.88	0.587	0.916	0.604
**2. Work**	2	85	-2.85	0.00	3.55	-	-	0.250
**3. Local/long distance journeys**	5	-	-4.34	-4.00	3.27	0.545	0.917	0.556
**4. Holidays**	2	30	-4.94	-6.00	3.42	0.326	0.922	0.331
**5. Physical ability**	1	-	-5.64	-6.00	2.81	0.576	0.916	0.582
**6. Family**	3	1	-5.14	-6.00	3.42	0.619	0.915	0.650
**7. Friendships and social life**	1	-	-4.59	-4.00	3.22	0.676	0.914	0.695
**8. Close personal relationship**	3	21	-4.13	-4.00	3.57	0.657	0.914	0.698
**9. Sex life**	1	35	-3.69	-4.00	3.24	0.611	0.915	0.638
**10. Physical appearance**	0	-	-2.92	-2.00	2.99	0.650	0.942	0.688
**11. Self-confidence**	1	-	-3.40	-2.00	3.14	0.659	0.914	0.702
**12. Motivation**	0	-	-4.20	-4.00	3.05	0.696	0.913	0.730
**13. People react to me**	2	-	-1.45	0.00	2.22	0.542	0.917	0.584
**14. Spiritual/religious life**	2	104	-0.74	0.00	2.03	-	-	0.349
**15. Feelings about the future**	1	-	-5.20	-6.00	3.35	0.682	0.913	0.701
**16. Financial situation**	1	-	-3.79	-4.00	3.50	0.490	0.918	0.519
**17. Living conditions**	2	-	-3.09	-2.00	3.26	0.609	0.915	0.665
**18. Dependence on others**	3	-	-3.95	-4.00	3.27	0.660	0.914	0.695
**19. People fuss/worry**	1	-	-3.64	-4.00	3.16	0.564	0.916	0.582
**20. Freedom to eat**	0	-	-4.56	-4.00	3.24	0.557	0.916	0.570
**21. Freedom to drink**	0	-	-4.84	-4.00	3.35	0.531	0.917	0.543

*Total Cronbach's alpha = 0.920.

These findings suggest that item 14 (spiritual/religious life) should be removed for both groups, as the majority of people reported either not having a spiritual or religious life (54.4% transplant group, 60.4% non-transplant group), or reported that it was not impacted by their renal condition (70.8% transplant group, 52.8% non-transplant group). Removing item 19 (fuss or worry) would improve the psychometrics of the questionnaire for transplant recipients. For the non-transplant group, treating item 2 (work) and item 4 (holidays) as separate items to the AWI scores may be indicated. Although the holidays item was relevant for the majority of the patients (93.6% transplant group, 73.8% non-transplant group), the analyses indicate that its removal from the overall AWI scores would improve the factor structure and reliability for the non-transplant group.

Supplementary Table 4. Reliability analysis and forced one-factor solution Principal Axis Factoring (PAF) on 17 items of the RDQoL weighted impact scores for the transplant group (*n*=142) and non-transplant group (*n*=158).

	**Transplant group**			**Non-transplant group**		
	**Corrected Item-total correlation**	**Cronbach's alpha if item deleted***	**PAF Item Loadings**	**Corrected Item-total correlation**	**Cronbach's alpha if item deleted***	**PAF Item Loadings**
**1. Leisure activities**	0.708	0.934	0.729	0.575	0.915	0.596
**3. Local/long distance journeys**	0.592	0.937	0.608	0.529	0.916	0.550
**5. Physical ability**	0.784	0.932	0.812	0.547	0.915	0.565
**6. Family**	0.727	0.934	0.753	0.622	0.913	0.658
**7. Friendships/social life**	0.761	0.933	0.791	0.666	0.912	0.696
**8. Close personal relationship**	0.718	0.934	0.747	0.658	0.912	0.695
**9. Sex life**	0.727	0.934	0.751	0.622	0.913	0.650
**10. Physical appearance**	0.557	0.937	0.577	0.645	0.913	0.674
**11. Self-confidence**	0.786	0.932	0.810	0.673	0.912	0.714
**12. Motivation**	0.817	0.932	0.848	0.709	0.911	0.744
**13. People react to me**	0.627	0.937	0.650	0.553	0.916	0.585
**15. Feelings about the future**	0.570	0.938	0.584	0.672	0.912	0.699
**16. Financial situation**	0.686	0.935	0.701	0.497	0.917	0.516
**17. Living conditions**	0.662	0.935	0.689	0.623	0.913	0.651
**18. Dependence on others**	0.496	0.939	0.512	0.637	0.913	0.668
**20. Freedom to eat**	0.611	0.936	0.629	0.553	0.915	0.572
**21. Freedom to drink**	0.587	0.937	0.600	0.530	0.916	0.548

*Transplant group total Cronbach's alpha 0.939; non-transplant group total Cronbach's alpha 0.918

Running the analyses again with the removal of items 2 (work), 4 (holidays), 14 (spiritual/religious life), and 19 (fuss/worry) for both groups indicated an acceptable factor structure and good reliability (Table 7). Therefore, to be able to calculate the AWI scores for all participants, without negatively impacting the psychometrics of the questionnaires, it was decided that item 2 (work) and 4 (holidays) items would be considered as separate items, whilst items 14 (spiritual/religious life) and 19 (fuss/worry) would be removed.

RTSQs

Factor structure: Parallel analysis indicated that a one-factor solution was optimal for both the transplant and non-transplant groups (see Table 8). For the transplant group, Principal Axis Factoring with a forced one-factor solution explained 50.65% of the variance, with item loadings from 0.521 to 0.814 (KMO=0.88, $\chi^2_{(78)}$=1197.74, p<0.001). Similar results were found with the non-transplant group, with 50.80% of the variance being explained, with item loadings from 0.452 to 0.848 (KMO=0.91, $\chi^2_{(78)}$=1321.69, p<0.001).

Reliability analyses: Previous research on the 11-item RTSQs[24] indicated that a forced one-factor solution was acceptable and up to 5 items could be tolerated whilst retaining an alpha coefficient of 0.79. Reliability analyses on the 13-item RTSQs revealed that it has excellent reliability for the transplant (α=0.905) and non-transplant groups (α=0.918). The removal of item 3 (side effects of treatment) would improve the alpha coefficient for both groups, but the item was correlated with the majority of the other items above 0.30, indicating that it should be included. All other items contributed to the reliability of the measure. It was possible to remove up to five items in this 13-item measure and retain a Cronbach's α coefficient above 0.80 for the transplant (α = 0.806) and non-transplant groups (α = 0.832). These findings confirmed that optimal scoring of the RTSQs includes all 13 items.

Supplementary Table 5. Descriptive statistics, reliability analysis, and forced one-factor solution Principal Axis Factoring (PAF) on the RTSQs items, completed by the transplant and non-transplant groups.

	Missing *N*	Mean	Median	*SD*	Corrected Item-total correlation	Cronbach's alpha if item deleted*	PAF Item Loadings
**Transplant group (*N* = 156)**							
**1. Overall satisfaction score**	0	5.58	6.00	0.78	0.707	0.896	0.740
**2. Control of renal condition**	0	5.48	6.00	0.83	0.668	0.897	0.721
**3. Side effects**	1	4.44	5.00	1.51	0.547	0.908	0.558
**4. Demands of treatment**	0	5.23	5.00	0.93	0.732	0.893	0.756
**5. Convenience of treatment**	0	5.23	6.00	1.08	0.730	0.893	0.763
**6. Flexibility of treatment**	0	5.22	6.00	1.16	0.518	0.904	0.559
**7. Freedom on treatment**	0	5.52	6.00	0.81	0.701	0.896	0.745
**8. Understanding of treatment**	0	5.63	6.00	0.69	0.526	0.902	0.569
**9. Time taken by treatment**	0	5.43	6.00	0.93	0.755	0.892	0.814
**10. Discomfort or pain**	1	5.32	6.00	1.15	0.522	0.904	0.521
**11. Treatment fits in with lifestyle**	0	5.41	6.00	1.02	0.700	0.894	0.745
**12. Recommend treatment to others**	1	5.75	6.00	0.70	0.607	0.900	0.634
**13. Satisfaction to continue treatment**	1	5.71	6.00	0.70	0.647	0.898	0.684
**Non-transplant group (*N* = 190)**							
**1. Overall satisfaction score**	1	4.99	5.00	1.09	0.522	0.917	0.546
**2. Control of renal condition**	0	4.94	5.00	1.03	0.552	0.916	0.576
**3. Side effects**	0	4.04	4.00	1.51	0.606	0.914	0.635
**4. Demands of treatment**	1	3.97	4.00	1.62	0.786	0.906	0.821
**5. Convenience of treatment**	0	4.19	5.00	1.64	0.812	0.905	0.848
**6. Flexibility of treatment**	0	4.30	5.00	1.71	0.730	0.909	0.764
**7. Freedom on treatment**	0	3.78	4.00	1.78	0.732	0.909	0.764
**8. Understanding of treatment**	0	5.16	5.00	1.05	0.430	0.919	0.452
**9. Time taken by treatment**	0	4.03	4.00	1.77	0.763	0.907	0.795
**10. Discomfort or pain**	1	4.39	5.00	1.46	0.557	0.915	0.579
**11. Treatment fits in with lifestyle**	1	3.77	4.00	1.65	0.699	0.910	0.726
**12. Recommend treatment to others**	3	5.05	6.00	1.31	0.619	0.913	0.642
**13. Satisfaction to continue treatment**	1	4.68	5.00	1.62	0.619	0.913	0.646

*Transplant group total Cronbach's alpha = 0.905. Non-transplant total Cronbach's alpha =0.918

RTSQc

Factor structure: See Table 9 for summaries of descriptive statistics, reliability analyses, and the factor structure of the RTSQc. Parallel analysis indicated that a one-factor solution was acceptable for the transplant and non-transplant groups. Principal Axis Factoring with a forced one-factor solution explained 56.22% of the variance with item loadings from 0.466 to 0.832 (KMO=0.90, $\chi^2_{(78)}$ =1353.40, p<0.001). Similar results were found for the non-transplant groups; 67.87% of the variance was explained, with item loadings from 0.601 to 0.935 (KMO=0.93, $\chi^2_{(78)}$ =1929.77, p<0.001).

Reliability analyses: Analyses revealed that the RTSQc has excellent reliability for the transplant (α=0.930) and non-transplant group (α=0.960). The removal of item 8 (understanding of treatment) would improve the alpha coefficient slightly for both groups. Despite this, item was 8 correlated with other items above 0.30, and the item loadings were acceptable, indicating that it should be included. Reliability analyses indicated that in this 13-item measure, it was possible to remove up to six items and retain a Cronbach's α coefficient of 0.826 for the transplant group and 0.906 for the non-transplant group.

In line with previous research, these findings suggest that the RTSQs and RTSQc can be considered to have a one-factor structure across various treatment groups and time points. Although the exclusion of various items is possible for the RTSQs and RTSQc, all items contribute to the factor structure, and the reliability is excellent using all 13 items.

Supplementary Table 6. Descriptive statistics, reliability analysis, and forced one-factor solution Principal Axis Factoring (PAF) on the RTSQc items, completed by transplant (DD and LD) and non-transplant (HD and PD) groups.

	Missing *N*	Mean	Median	*SD*	Corrected Item-total correlation	Cronbach's alpha if item deleted*	PAF Item Loadings for 1 factor solution
**LD and DD groups (*n*=150)**							
**1. Overall satisfaction score**	3	2.42	3.00	1.07	0.702	0.924	0.723
**2. Control of renal condition**	3	2.50	3.00	1.03	0.720	0.923	0.748
**3. Side effects**	3	1.69	2.00	1.54	0.599	0.930	0.616
**4. Demands of treatment**	4	2.32	3.00	1.08	0.800	0.920	0.833
**5. Convenience of treatment**	4	2.27	3.00	1.26	0.783	0.920	0.824
**6. Flexibility of treatment**	4	2.26	3.00	1.21	0.678	0.924	0.714
**7. Freedom on treatment**	4	2.33	3.00	1.09	0.796	0.921	0.832
**8. Understanding of treatment**	5	2.17	3.00	1.15	0.449	0.932	0.486
**9. Time taken by treatment**	5	2.37	3.00	1.07	0.739	0.922	0.764
**10. Discomfort or pain**	8	2.18	3.00	1.24	0.616	0.927	0.628
**11. Treatment fits in with lifestyle**	7	2.32	3.00	1.16	0.760	0.922	0.803
**12. Recommend treatment to others**	6	2.58	3.00	0.99	0.564	0.928	0.581
**13. Satisfaction to continue treatment**	7	2.50	3.00	1.08	0.787	0.921	0.814
**HD and PD groups (*n*=170)**							
**1. Overall satisfaction score**	0	1.30	2.00	1.73	0.788	0.957	0.802
**2. Control of renal condition**	0	1.48	2.00	1.60	0.676	0.960	0.686
**3. Side effects**	0	0.89	1.00	1.77	0.777	0.957	0.791
**4. Demands of treatment**	0	0.96	1.00	1.72	0.888	0.955	0.910
**5. Convenience of treatment**	0	0.98	1.00	1.82	0.912	0.954	0.935
**6. Flexibility of treatment**	0	0.91	1.00	1.68	0.832	0.956	0.852
**7. Freedom on treatment**	0	0.62	1.00	1.86	0.941	0.956	0.862
**8. Understanding of treatment**	0	1.76	2.00	1.27	0.592	0.961	0.601
**9. Time taken by treatment**	0	0.89	2.00	1.80	0.862	0.955	0.884
**10. Discomfort or pain**	0	0.91	2.00	1.57	0.733	0.958	0.746
**11. Treatment fits in with lifestyle**	0	0.73	2.00	1.73	0.851	0.955	0.871
**12. Recommend treatment to others**	0	1.69	3.00	1.59	0.737	0.958	0.748
**13. Satisfaction to continue treatment**	0	1.39	1.00	1.73	0.748	0.958	0.761

*LD and DD groups total Cronbach's alpha = 0.930. HD and PD groups total Cronbach's alpha = 0.960. LD=living donor; DD= deceased donor; HD=haemodialysis' PD= peritoneal dialysis.

References

1. Bradley C. Design of a Renal-Dependent Individualized Quality of Life Questionnaire. *Adv Perit Dial* 1997; 13: 116-120.
2. Bradley C, Todd C, Gorton T, et al. The development of an individualised measure of perceived impact of diabetes on quality of life: The ADDQoL. *Qual Life Res* 1999; 8(1-2): 79-91.doi:10.1023/A:1026485130100.
3. Wee HL, Tan CE, Goh SY, Li SC. Usefulness of the Audit of Diabetes-Dependent Quality-of-Life (ADDQoL) Questionnaire in patients with diabetes in a multi-ethnic Asian country. *Pharmacoeconomics* 2006; 24(7): 673-682.
4. Bradley C, Lewis KS. Measures of psychological well-being and treatment satisfaction developed from the responses of people with tablet-treated diabetes. Diabetic Med 1990; 7, 445-451.
5. Bradley C. The Diabetes Treatment Satisfaction Questionnaire: DTSQ. In Bradley C. (Ed). Handbook of Psychology and Diabetes: A guide to psychological measurement in diabetes research and practice. Chur Switzerland: Harwood Academic Publishers, 1994; 111-132.
6. Howorka K, Pumprla J, Schlusche C, et al. Dealing with ceiling baseline treatment satisfaction level in patients with diabetes under flexible, functional insulin treatment: Assessment of improvements in treatment satisfaction with a new insulin analogue. *Qual Life Res* 2000; 9: 915-930.
7. Barendse SM, Speight J, Bradley C. The Renal Treatment Satisfaction Questionnaire (RTSQ): A measure of satisfaction with treatment for chronic kidney failure. *Am J Kidney Dis* 2005; 45: 572-579.
