# Supplementary material for: Changes in quality of life (QoL) and other patient-reported outcome measures (PROMs) in living-donor and deceased-donor kidney transplant recipients and those awaiting transplantation in the UK ATTOM programme: a longitudinal cohort questionnaire survey with additional qualitative interviews
Source: BMJ Open. 2021 Apr 8;11(4):e047263. doi: 10.1136/bmjopen-2020-047263 (PMC8098938; doi:10.1136/bmjopen-2020-047263)
Supplement: Author's manuscript [file bmjopen-2020-047263.draft_revisions.pdf]

BMJ Open is committed to open peer review. As part of this commitment we make the peer review history of every article we publish publicly available.

When an article is published we post the peer reviewers' comments and the authors' responses online. We also post the versions of the paper that were used during peer review. These are the versions that the peer review comments apply to.

The versions of the paper that follow are the versions that were submitted during the peer review process. They are not the versions of record or the final published versions. They should not be cited or distributed as the published version of this manuscript.

BMJ Open is an open access journal and the full, final, typeset and author-corrected version of record of the manuscript is available on our site with no access controls, subscription charges or pay-per-view fees (http://bmjopen.bmj.com).

If you have any questions on BMJ Open's open peer review process please email info.bmjopen@bmj.com

BMJ Open

Changes in quality of life (QoL) and other patient-reported outcome measures (PROMs) in living-donor and deceased-donor kidney transplant recipients and those awaiting transplantation in the UK ATTOM programme: a longitudinal cohort questionnaire survey with additional qualitative interviews.

Journal: *BMJ Open*

Manuscript ID: bmjopen-2020-047263

Article Type: Original research

Date Submitted by the Author: 02-Dec-2020

Complete List of Authors:
Gibbons, Andrea; University of Winchester Department of Psychology; Royal Holloway University of London, Health Psychology Research Unit
Bayfield, Janet; Royal Holloway University of London, Health Psychology Research Unit; Health Psychology Research (HPR) Ltd
Cinnirella, Marco; Royal Holloway University of London, Psychology
Draper, Heather; University of Warwick Warwick Medical School, Centre for Biomedical Ethics
Johnson, Rachel; NHS Blood and Transplant Organ Donation and Transplantation Directorate
Oniscu, Gabriel; Royal Infirmary of Edinburgh, Edinburgh Transplant Centre
Ravanan, Rommel; Southmead Hospital, Richard Bright Renal Unit
Tomson, Charles; Newcastle Upon Tyne Hospitals NHS Foundation Trust
Roderick, Paul; University of Southampton Faculty of Medicine, Academic Unit of Primary Care and Population Sciences, Faculty of Medicine
Metcalfe, Wendy; Royal Infirmary of Edinburgh, Transplant Unit
Forsythe, John L. R.; Royal Infirmary of Edinburgh, Edinburgh Transplant Centre; NHS Blood and Transplant Organ Donation and Transplantation Directorate, Organ Donation and Transplantation
Dudley, Christopher; Southmead Hospital, Richard Bright Renal Unit
Watson, Christopher; University of Cambridge, NIHR Cambridge Biomedical Research Centre and the NIHR Blood and Transplant Research Unit in Organ Donation and Transplantation ; Addenbrooke's Hospital
Bradley, J Andrew; University of Cambridge, NIHR Cambridge Biomedical Research Centre and the NIHR Blood and Transplant Research Unit in Organ Donation and Transplantation ; Addenbrooke's Hospital
Bradley, Clare; Health Psychology Research (HPR) Ltd; Royal Holloway University of London, Health Psychology Research Unit

Keywords: Renal transplantation < NEPHROLOGY, TRANSPLANT SURGERY, NEPHROLOGY

I, the Submitting Author has the right to grant and does grant on behalf of all authors of the Work (as defined in the below author licence), an exclusive licence and/or a non-exclusive licence for contributions from authors who are: i) UK Crown employees; ii) where BMJ has agreed a CC-BY licence shall apply, and/or iii) in accordance with the terms applicable for US Federal Government officers or employees acting as part of their official duties; on a worldwide, perpetual, irrevocable, royalty-free basis to BMJ Publishing Group Ltd ("BMJ") its licensees and where the relevant Journal is co-owned by BMJ to the co-owners of the Journal, to publish the Work in this journal and any other BMJ products and to exploit all rights, as set out in our licence.

The Submitting Author accepts and understands that any supply made under these terms is made by BMJ to the Submitting Author unless you are acting as an employee on behalf of your employer or a postgraduate student of an affiliated institution which is paying any applicable article publishing charge ("APC") for Open Access articles. Where the Submitting Author wishes to make the Work available on an Open Access basis (and intends to pay the relevant APC), the terms of reuse of such Open Access shall be governed by a Creative Commons licence – details of these licences and which Creative Commons licence will apply to this Work are set out in our licence referred to above.

Other than as permitted in any relevant BMJ Author's Self Archiving Policies, I confirm this Work has not been accepted for publication elsewhere, is not being considered for publication elsewhere and does not duplicate material already published. I confirm all authors consent to publication of this Work and authorise the granting of this licence.

For peer review only

Changes in quality of life (QoL) and other patient-reported outcome measures (PROMs) in living-donor and deceased-donor kidney transplant recipients and those awaiting transplantation in the UK ATTOM programme: a longitudinal cohort questionnaire survey with additional qualitative interviews.

Andrea Gibbons (0000-0002-7774-0563), Janet Bayfield, Marco Cinnirella, Heather Draper, Rachel J Johnson, Gabriel C Oniscu, Rommel Ravanan, Charles RV Tomson, Paul Roderick, Wendy Metcalfe, John LR Forsythe, Christopher Dudley, Christopher JE Watson, J Andrew Bradley, Clare Bradley.

Department of Psychology, University of Winchester, Winchester, SO22 4NR, UK; the Health Psychology Research Unit, Royal Holloway, University of London, Egham, Surrey, TW20 0EX, UK, Andrea Gibbons, Lecturer in Psychology

Health Psychology Research Unit, Royal Holloway, University of London, Egham, Surrey, TW20 0EX, UK. Janet Bayfield, Administrator/Research Assistant

Department of Psychology, Royal Holloway, University of London, Egham, Surrey, TW20 0EX, UK, Marco Cinnirella, Professor of Applied Social Psychology

Division of Health Sciences, University of Warwick, Warwick Medical School, Coventry, CV4 7AL, UK, Heather Draper, Professor of Biomedical Ethics

NHS Blood and Transplant, Fox Den Road, Stoke Gifford, Bristol, BS34 8RR, UK, Rachel J. Johnson, Assistant Director - Statistics and Clinical Studies

Edinburgh Transplant Centre, Royal Infirmary of Edinburgh, 51 Little France Crescent, Edinburgh, EH16 4SA, UK, Gabriel C. Oniscu, Consultant Transplant Surgeon and Honorary Reader

Richard Bright Renal Unit, Southmead Hospital, Southmead Road, Bristol, BS10 5NB, UK, Rommel Ravanan, Consultant Nephrologist

Department of Renal Medicine, Freeman Hospital, Newcastle upon Tyne, NE12 9NY, UK, Charles R. V. Tomson, Consultant Nephrologist

Academic Unit of Primary Care and Population Sciences, Faculty of Medicine, University of Southampton, Tremona Road, Southampton, SO16 6YD, UK, Paul Roderick, Professor of Public Health

Edinburgh Transplant Centre, Royal Infirmary of Edinburgh, 51 Little France Crescent, Edinburgh, EH16 4SA, UK, Wendy Metcalfe, Consultant Nephrologist

Edinburgh Transplant Centre, Royal Infirmary of Edinburgh, 51 Little France Crescent, Edinburgh, EH16 4SA, UK, and Organ Donation and Transplantation, NHS Blood and Transplant, Fox Den Road, Stoke Gifford, Bristol, BS34 8RR, UK, John L. R. Forsythe, Honorary Professor, University of Edinburgh and Medical Director of Organ Donation and Transplantation, NHS Blood and Transplant

Richard Bright Renal Unit, Southmead Hospital, Southmead Road, Bristol, BS10 5NB, UK, Christopher Dudley, Consultant Nephrologist

For peer review only - http://bmjopen.bmj.com/site/about/guidelines.xhtml

Department of Surgery, University of Cambridge, and the NIHR Cambridge Biomedical Research Centre and the NIHR Blood and Transplant Research Unit in Organ Donation and Transplantation at the University of Cambridge, Addenbrooke's Hospital, Hills Road, Cambridge, CB2 0QQ, UK, Christopher J. E. Watson, Professor of Transplantation

Department of Surgery, University of Cambridge and the NIHR Cambridge, Biomedical Research Centre, and the NIHR Blood and Transplant Research Unit in Organ Donation and Transplantation, University of Cambridge, Addenbrooke's Hospital, Hills Road, Cambridge, CB2 0QQ, UK. J. Andrew Bradley, Emeritus Professor

Health Psychology Research (HPR) Ltd, 188 High Street, Egham, Surrey, TW20 9ED; Health Psychology Research Unit, Royal Holloway, University of London, Egham, Surrey, TW20 0EX, UK. Clare Bradley Professor emerita, Royal Holloway, Head of the Health Psychology Research Unit and CEO of HPR Ltd.

Correspondence to: Clare Bradley cb@healthpsychologyresearch.com

For peer review only

Abstract

Objective: To examine quality of life (QoL) and other patient-reported outcome measures (PROMs) in kidney transplant recipients and those awaiting transplantation.

Design: Longitudinal cohort questionnaire surveys and qualitative semi-structured interviews using thematic analysis using a pragmatic approach.

Setting: Completion of generic and disease-specific PROMs at two time points, and telephone interviews with participants UK-wide.

Participants: 101 incident deceased-donor (DD) and 94 incident living-donor (LD) kidney transplant recipients, together with 165 patients on the waiting list (WL) from 18 UK centres recruited to the Access to Transplantation and Transplant Outcome Measures (ATTOM) programme completed PROMs at recruitment (Nov 2011-March 2013) and one-year follow-up. Forty-one of the 165 WL patients received a DD transplant and 26 a LD transplant during the study period, completing PROMs initially as WL patients, and again one-year post-transplant. A subsample of 10 LD and 10 DD recipients participated in qualitative semi-structured interviews.

Results: LD recipients were younger, had more educational qualifications and more often received a transplant before dialysis. Controlling for these and other factors, cross-sectional analyses at 12-months post-transplant suggested better QoL, renal-dependent QoL and treatment satisfaction for LD than DD recipients. WL patients reported worse outcomes compared with both transplant groups. However, longitudinal analyses (controlling for pre-transplant differences) showed that LD and DD recipients reported similarly improved health status and renal- dependent QoL ($p<0.01$) pre- to post-transplant. WL patients had worsened health status but no change in QoL. Qualitative analyses revealed transplant recipients' expectations influenced their recovery and satisfaction with transplant.

Conclusions: Whilst cross-sectional analyses suggested LD kidney transplantation leads to better QoL and treatment satisfaction, longitudinal assessment showed similar QoL improvements in PROMs for both transplant groups, with better outcomes than for those still wait-listed. Regardless of

For peer review only - http://bmjopen.bmj.com/site/about/guidelines.xhtml

transplant type, clinicians need to be aware that managing expectations is important for facilitating patients' adjustment post-transplant.

Keywords: living donor, deceased donor, kidney transplantation, quality of life, PROMs

Article summary

Strengths and limitations of this study

- We examined a number of patient-reported outcomes in people requiring kidney transplantation, including quality of life, well-being, treatment satisfaction, and health status.
- We collected pre-transplant data for a subsample of deceased-donor and living-donor kidney transplant recipients whilst they were still waiting for a transplant, and assessed outcomes from pre-transplant to one-year post-transplant.
- Controlling for various medical and demographic factors, including age, time on dialysis and education, impacts the findings, removing apparent benefits of living-donor kidneys over deceased-donor kidneys.
- The sample of kidney transplant recipients with pre-transplant data is relatively small (n=67 including 26 with living donors and 41 with deceased donors).
- Longer follow-up may be required to examine fully any differences in PROMs between groups post-transplant.

For peer review only - http://bmjopen.bmj.com/site/about/guidelines.xhtml

Introduction

Healthcare usually aims to improve health and minimise disability,[1] and there is growing acknowledgment that patient-reported outcome measures (PROMs) are important for evaluating the effectiveness of healthcare. PROMs are measures of outcomes that are directly reported by the patient, and are usually questionnaires that can be generic or condition-specific. PROMs may measure a wide range of outcomes including health status, QoL, treatment satisfaction, well-being, and other outcomes such as symptoms. Despite their importance in assessing the patient's perspective, PROMs such as quality of life are not measured routinely as part of the evaluation of surgical interventions.[1] Part of the reason for this has been the belief that brief health-status tools (such as the EuroQoL-5D (EQ-5D) captures quality of life (QoL)[1], as well as the time and cost involved in collecting and interpreting the data.[2,3] However, PROMs other than health status tools have an important role in clinical care[3] as they may determine the comparative benefits of different medical interventions used to treat the same condition.[4]

One example of a condition that can benefit from the measurement of PROMs is advanced chronic kidney disease (CKD Grade (G) 5). Kidney transplantation is commonly considered to be the best medical treatment for most people with advanced CKD G5 who are fit enough for the procedure.[5] Recent changes to the kidney donor system, such as deemed consent legislation in Wales since 2015, and a similar system introduced in England in May 2020, and due to be enacted in Scotland in 2021, aim to increase the number of deceased-donor (DD) kidneys available.[6] Kidney transplant waiting times have also reduced in recent years due to an increase in unrelated living donation including altruistic donation, and kidney sharing schemes that allow for greater access to living-donor (LD) transplantation for blood-group or HLA incompatible donor-recipient pairs.[7] Transplantation success is most often measured by patient and graft survival and as such LD transplantation provides a greater survival advantage when compared directly with DD transplantation, controlling for differences in variables such as age[8] and time on dialysis.[9] LD transplantation is associated with lower rejection rates[7] and spares individuals the uncertain wait for

For peer review only - http://bmjopen.bmj.com/site/about/guidelines.xhtml

a deceased-donor organ.[7] However, it is important to note that LD recipients tend to have better clinical indices of health pre-transplant. [10,11] These potential confounding factors can impact graft survival,[12] so pre-transplant differences between LD and DD recipients should be measured and controlled, before comparisons are made between the groups. The other issue apparent with these studies is that they focus on the comparative benefits of LD and DD transplantation for health, but not for other outcomes such as QoL.

When PROMs have been assessed in kidney transplantation, cross-sectional post-transplant comparisons indicated that LD recipients reported greater social involvement and happiness compared with DD recipients.[13] When cross-sectional data are analysed, it is important to have well-matched controls but in practice that is difficult to achieve because those who are left on the waiting list for deceased-donor kidneys tend to have more health problems than those who receive a transplant. Studies that have controlled for underlying differences across the groups, such as differences in age or comorbid disease, report similar outcomes for both transplant groups on health status measures such as the SF-36,[13] although many LD recipients report experiencing feelings of guilt about the risks to their donor.[14,15] Longitudinal studies, which can include and control for baseline measures of outcomes before participants receive a transplant, suggest that improvements in health status can be seen in the first few months post-transplant for both DD and LD, but that these improvements remain stable after this time.[16-19] However, these studies lack any group with which to compare transplant recipients, such as those still awaiting transplantation, and not all include data pre-transplant. Those who are transplanted are more likely to be in better health than those patients still wait-listed for a transplant, so it is important to control for baseline differences between participants who receive a transplant and those who do not.

One major problem with of all of these studies is that the instruments used to measure quality of life (QoL) such as the SF-36, actually measure health status.[20] Health status includes aspects of a person's life such as their physical ability, daily functioning, and experience of symptoms. Health-related QoL is a term that is commonly and misleadingly used to refer to health

For peer review only - http://bmjopen.bmj.com/site/about/guidelines.xhtml

status tools such as the SF-36 and EQ-5D, which may assess health and functioning but do not assess the impact of health and functioning on QoL. In contrast, QoL as measured by the Renal Dependent QoL (RDQoL) measure used in ATTOM,[21] is defined within the questionnaire as 'how good or bad you feel your life to be'. RDQoL, modelled on the ADDQoL for diabetes[22] and influenced by McGee et al's[23] individualised interview method of generic QoL measurement, establishes the relevance of different aspects of life for each individual respondent and excludes non-relevant items from the scoring. It allows for individual differences in the importance of relevant aspects of life for QoL as well as measuring the impact of the renal condition on each relevant aspect of life. Impact is weighted by importance and an average weighted impact (AWI) score obtained for each individual indicating the nature and extent of the impact of the renal condition on the individual's QoL. A single item to measure generic QoL is also included in the measure and this item can be expected to be more strongly related to health status generally than the condition-specific AWI score.[24] Given the confusion surrounding the definitions and measurement of these concepts, it is important that research includes genuine measures of QoL and condition-dependent QoL as well as a health status measure when investigating PROMs in people with CKD. The addition of qualitative research methods alongside PROMs, can be valuable in providing more detailed insight into how transplant recipients experience transplantation and how it impacts QoL.

The main objectives of the present study were 1) to examine QoL (generic and condition-dependent), health status, and other PROMs in recipients of DD or LD transplants, and 2) to compare them over time with those on the waiting list (WL) for a DD transplant in a matched cross-sectional cohort and in a subsample with longitudinal data pre- and post-transplant. Specifically, it was hypothesised that, controlling for potential confounding factors, there would be very few differences in outcomes between DD and LD recipients. It was also hypothesised that transplant recipients would report less negatively impacted QoL and better scores on other PROMs than participants waiting for a transplant. The third objective was to use qualitative interview methods to

For peer review only - http://bmjopen.bmj.com/site/about/guidelines.xhtml

explore in more depth the expectations and experience of transplantation and how it influences QoL one-year post-transplant in DD and LD transplant recipients.

Methods

Study design, participants and procedure.

This study employed a longitudinal cohort survey design, as well as a qualitative interview approach. The study was part of the UK Access to Transplantation and Transplant Outcome Measures (ATTOM) research programme.[25] ATTOM was based on a large national observational cohort study involving all kidney units in the UK in order to examine the reasons for disparities in access to renal transplantation.[26,27] Full ATTOM methodology has been reported elsewhere.[25] The current study was conducted within a work-stream examining detailed PROMs in patients fluent in English, aged less than 75 years, receiving renal-replacement therapy (RRT) who completed measures of health status, well-being, QoL, and treatment satisfaction. Qualitative interviews were conducted with a subset of patients one-year post-transplant.

Following ethical approval (East of England Research Ethics Committee 11/EE/0120), and informed consent, participants were recruited by ATTOM research nurses to the study as incident recipients of DD transplants (*n*=104), incident LD transplant recipients (*n*=94), or patients on the waiting list for a transplant (*n*=165) and followed up either one-year post-recruitment or one-year post-transplant (Figure 1). Across 18 UK renal centres, a subset of participants recruited to ATTOM were recruited to this work-stream. The first eligible patient for each transplant group seen each month (November 2011 to March 2013) by each nurse was invited to take part. Inclusion criteria were fluency in English and <75 years of age. Participants were recruited if they had G5 CKD and received a DD or LD transplant within one month. The UK Transplant Registry identified possible matched controls for recruited transplant recipients every two weeks, and members of this list of potential participants were then invited to take part as WL patients. Participants on the WL were matched to DD transplant recipients as closely as possible in terms of renal centre, age (+/- 5 years),

For peer review only - http://bmjopen.bmj.com/site/about/guidelines.xhtml

time on the waiting list (+/-100 days), and previous type of RRT. Participants completed measures of health status and well-being when first recruited to ATTOM by the research nurses, and measures of generic and renal-dependent QoL, and renal treatment satisfaction three months post-transplant/post recruitment via telephone or post. A timeframe of three months was chosen to allow participants time to experience their treatment (i.e. transplant for those receiving a transplant) and be able to reflect on how their QoL and treatment satisfaction were impacted after their return home for those having surgery. At one year, participants were contacted via telephone and completed all measures again (via telephone or post), as well as a measure of change in treatment satisfaction which compared satisfaction with current renal treatment and previous renal treatment. One-year follow-up was chosen because it would be expected that clinical outcomes would be stable one-year post-transplant, but it's not clear whether non-clinical outcomes (i.e. PROMs) would also remain stable. At the same time, choosing one-year follow-up allows for comparisons to be made with previous research that has examined health status using the same timeline. A sample of 100 participants in each of the three groups was calculated as adequate to reach 80% power within the planned analyses. Some of the participants recruited as WL patients subsequently received a DD (n=41) or LD kidney transplant (n=26) in the year of follow-up. For these participants, the initial measures were completed while they were still on the WL, giving a pre-transplant measure, and the second set of measures was completed one-year post-transplant (Figure 1). These data allowed for secondary analyses in patients for whom we had true baseline measures comparing the two transplant groups and those remaining on the waiting list pre- and post-transplant. Participants recruited as WL comparison patients who went on to receive a transplant within ATTOM were not asked to complete the measures at 3 months post-transplant in the same way as those recruited to the study as transplant recipients were asked to do. It was not always possible to contact people within three months of receiving their transplant once they had been recruited as WL patients, and a limit of 4 points of data collection were set for each participant, to minimise participant burden.

For peer review only - http://bmjopen.bmj.com/site/about/guidelines.xhtml

A subset of transplant recipients was selected for qualitative telephone interviews. The aim of this qualitative study was to explore the experience of receiving a transplant, and how having a transplant affects QoL and other PROMs. The sample consisted of 10 DD transplant and 10 LD transplant recipients. All participants had been contacted previously by authors (AG or JB) when arranging completion of questionnaires. Participants were invited to take part based on their average weighted impact (AWI) scores on the Renal Dependent Quality of Life (RDQoL) questionnaire,[21] completed one-year post-transplant. Participants with AWI scores above or below one standard deviation of the mean score, as well as those with a mean score, were included so that participants had varying levels of QoL. The qualitative sample was selected on the basis of QoL scores to aid our understanding of the range of responses and how they may relate to variations in QoL. The profile of the qualitative-study participants reflected the age and sex profile of the larger sample. Participants were informed that the interview would explore their questionnaire responses related to their QoL and treatment satisfaction. Semi-structured telephone interviews were conducted between January and August 2014, and were conducted by a single interviewer using an interview schedule (Supplementary Data 1) guided by published literature and participants' questionnaire responses.[28] Interviews were conducted by a postdoctoral research fellow (AG) experienced in qualitative research and trained in use of NVivo software (QSR International, US) for qualitative analysis, with background in the field of health psychology. Field notes were made following every audio-recorded interview. Interview recordings (mean=52min, *SD*=13.4, range=31-76) were transcribed.

Patient and Public Involvement

Patients were involved in the development of the ATTOM project objectives and procedures, as well as analysis and dissemination. A patient representative from the National Kidney Federation (NKF) was involved in all aspects of the development of the research. A patient representative was also invited to all steering group meetings throughout the duration of the project. Findings were

For peer review only - http://bmjopen.bmj.com/site/about/guidelines.xhtml

presented to patients and lay people as part of an NIHR stakeholder ATTOM meeting in November 2017.

Outcomes

A summary of the primary outcome measures can be seen in Supplementary Data 2, Table 1. QoL was assessed using the RDQoL questionnaire,[21] developed from the Audit of Diabetes Dependent Quality of Life (ADDQoL) questionnaire.[22,29] It includes one overview item to measure generic QoL on a scale from excellent (+3) to extremely bad (−3). Twenty-one items measure the impact of the renal condition on specific aspects of life. Each specific item includes a rating of the impact of the renal condition on QoL, from -3 (most negative impact) to +1 (positive impact), and a rating of importance of that aspect of life for the individual's QoL ranging from very important (+3) to not at all important (0). Impact and importance ratings are multiplied to give a weighted impact score for each item, ranging from −9 (most negative/important impact of the renal condition on QoL) to +3 (most positive/important impact). Some items include preliminary questions to determine applicability to the individual (e.g. work). The weighted impact scores are summed and divided by the number of applicable items to give an average weighted impact (AWI) score, also ranging from −9 to +3. Principal axis factoring and reliability analyses confirmed that the RDQoL was appropriate for both transplant and non-transplant groups (see Supplementary Data 3, Tables 2-4).

Well-being was measured by the Well-being Questionnaire (W-BQ12).[30] Higher scores indicate greater well-being (range=36-0). Health status was measured by the EQ-5D-5L,[31] which has two sections. The first section includes five dimensions of health that are rated on five levels. These data were then converted into a population preference score called a health-utility value, using the new value set for England.[32] The second section asks participants to rate 'Your health today' on a visual analogue scale (EQ-VAS) from 100 (best health you can imagine) to 0 (worst health you can imagine). The EQ-VAS is a PROM, but the utility measure is not, as it depends not only on patients'

For peer review only - http://bmjopen.bmj.com/site/about/guidelines.xhtml

reports but also on the values assigned by the general population to each profile of scores for the five dimensions of health used to create the value set for England.

Satisfaction with current renal treatment was measured using the Renal Treatment Satisfaction Questionnaire status version (RTSQs).[33] It has 13 items, with 7-point scales (6 to 0), that are summed to give a total score (range=78-0). Higher scores indicate greater satisfaction with current renal treatment. The change version (RTSQc) is modelled on the change version of the Diabetes Treatment Satisfaction Questionnaire (DTSQc),[34] developed to counteract ceiling effects found with the status version (DTSQs).[35] It asks participants to compare satisfaction with current treatment with satisfaction with their previous treatment on 7-point scales (+3 to −3 where 0=no change) and is summed to give a total score from +39 (maximum satisfaction improvement) to -39 (maximum deterioration in satisfaction). Principal axis factoring and reliability analyses confirmed that the RTSQs and RTSQc were appropriate for both transplant and non-transplant groups (see Supplementary Data 3, Tables 5-6).

All sociodemographic and medical information was recorded by research nurses from medical notes at time of recruitment. Any condition mentioned in patients' records was identified and classified into groups (heart disease, heart failure, liver disease, diabetes, mental health problems, peripheral vascular disease). Prescribed immunosuppressant and steroid medication were also recorded. Height and weight were recorded to allow calculation of Body Mass Index (BMI). Any subsequent changes to information (such as change from being on the WL to receiving a transplant) were self-reported by participants.

Analysis

Reflecting previous research in this area, the main analyses involved cross-sectional differences at one-year follow-up in those who received a DD transplant (*n*=145), LD transplant (*n*=120), and those who remained waiting for a transplant (*n*=98) at one-year follow-up. These analyses were conducted using one-way Analysis of Covariance (ANCOVAs), controlling for medical

For peer review only - http://bmjopen.bmj.com/site/about/guidelines.xhtml

and demographic differences recorded at recruitment. Clinical factors such as previous renal replacement therapy, as well as demographic factors including age, and indicators of SES (such as car ownership and qualifications), can influence a person's perspective on their QoL and treatment satisfaction. We therefore assessed these variables and controlled for them in the analyses if differences between groups were identified (using Chi Squared tests or ANOVAs).[36] It was hypothesized that patients still on the WL would have more negative scores on PROMs than those who received a transplant.

Secondary analyses involved a prospective assessment of those participants recruited as patients on the WL (*n*=165). The sample was divided into three groups based on their treatment at follow-up; those who remained waiting for a transplant (*n*=98); those who subsequently had a DD (*n*=41); and those who subsequently had an LD transplant (*n*=26). This allowed for changes in outcomes to be measured pre- and post-transplant for these groups and for pre-transplant differences to be controlled for. Differences between groups and over time were investigated with a series of 3 (group) x 2 (time) ANCOVAs with planned comparisons on all PROMs and utility outcome scores (controlling for various factors). It was hypothesised that those who received a transplant would report improved PROMs, whilst those remaining on the waiting list would report either no change, or a deterioration in scores. It was also hypothesised that when differences in sociodemographic factors are controlled, there will be no differences in outcomes between DD and LD recipients. A one-way ANCOVA was also conducted to examine differences between these three groups (those who remained on the WL, those who subsequently had a DD or LD transplant) for change in satisfaction comparing treatment at one year (e.g. one-year post-transplant) with previous renal treatment (e.g. dialysis at recruitment) using RTSQc scores.

For the qualitative interview data,[37] thematic analysis based on a pragmatic and critical realist approach was used to explore the experiences of transplant recipients. Field notes were reviewed and transcripts read three times for familiarisation prior to analysis. The coding was completed in Microsoft Word, then entered into NVivo10 software for analysis. Using established

For peer review only - http://bmjopen.bmj.com/site/about/guidelines.xhtml

guidelines,[38] independent initial coding by AG established major themes derived from the data which enabled development of a coding framework (AG, CB, MC). There was substantial coder agreement (AG and JB) on 5 interviews; AG completed the remaining coding on subsequent interviews. Reiteration of responses indicated data saturation had been achieved after 20 interviews.

Results

Descriptive findings

Descriptive data for the groups at recruitment (165 patients recruited whilst on waiting list for a kidney transplant, 104 DD recipients, 94 LD recipients) are shown in Table 1. Almost 13 percent (12.5%) of DD recipients and 47.5% of LD recipients received their transplants before requiring dialysis. Almost eighteen percent (17.6%) of those on the waiting list were not yet on dialysis when recruited to the study. At recruitment, 47 (28.5%) patients on the WL had had a previous kidney transplant that failed, whilst 13 LD (13.8%) and 12 DD (11.5%) recipients had had a previous transplant.

Of all those who received a DD transplant during the study, 80 (55.2%) received a kidney from a donor after circulatory death (DCD), and 65 (44.8%) received a kidney from a donor after brain death (DBD). Sixty-nine LD recipients (57.5%) received a kidney from a relative (35 parent or adult child, 24 from a sibling and 10 from another relative e.g. aunt, uncle, niece, nephew, cousin), whilst 51 (42.5%) received a genetically unrelated donor kidney (via the paired/pooled exchange or altruistic donors).

In total, 82 (22.6%) participants did not complete measures at one year; 33 (9.1%) were not contactable, 39 (10.7%) did not return questionnaires, and 10 were known to have died (2.8%). Responders at one year were older ($t_{(120.55)}$= -2.4, p=0.017), and had better EQ-VAS health ratings ($t_{(347)}$= -2.9, p=0.005) at recruitment compared with non-responders. There were no differences in sex, employment status, civil status, education, or previous RRT between those who did and did not respond at one year. Responders at one year were more likely to be White (χ^2 =10.3, df=4, p=0.016)

For peer review only - http://bmjopen.bmj.com/site/about/guidelines.xhtml

than non-responders. Missing data were fewer than 5% for all variables and were missing at random, so cases were deleted analysis by analysis.

For those 20 participants who took part in the qualitative interviews, 10 were women, 13 were living with a partner or married, four were single, and three were separated or divorced. Nine were working, 8 were retired, and three were unemployed/long-term disabled. Six had had a previous transplant, and 17 were White, with the remaining three participants being Chinese, Asian, or identified as mixed race. Six were transplanted before requiring dialysis, 12 were previously on haemodialysis, and two received peritoneal dialysis prior to transplant.

<u>Quantitative findings</u>

LD recipients were significantly younger compared with the other groups ($F_{(3,375)}$=9.7, p<0.001; see Table 1). The majority of DD recipients and WL patients were on haemodialysis, whilst a larger proportion of LD recipients were transplanted before commencing dialysis (χ^2=23.3, df=2, p<0.001). LD recipients were more likely to own a car (χ^2=6.9, df=2, p=0.032). Those who remained on the WL differed from those who received a transplant on two marginally related factors (r=0.11, p=0.039); patients on the waiting list were more likely to have experience of a previous transplant that failed (χ^2=16.8, df=2; p<0.001) and experience of mental health problems (as recorded by research nurses from medical notes; χ^2=8.9, df=2; p=0.011). DD recipients and WL participants reported fewer educational qualifications than LD recipients (χ^2=13.7, df=6, p=0.033). All subsequent analyses controlled for age, previous RRT, previous experience of a transplant, education, car ownership, and history of mental health problems. Participants who were classified as obese at recruitment (BMI >30) or had a diagnosis of diabetes were more likely to experience worse QoL than those without either condition (ps<0.05). In separate research conducted as part of the ATTOM programme, obesity was associated with a greater likelihood of delayed graft function in DD transplant recipients, and diabetes was related to a greater risk of transplant failure in LD transplant recipients.[39] Analyses were therefore run controlling for obesity and diabetes. The difference in

For peer review only - http://bmjopen.bmj.com/site/about/guidelines.xhtml

For peer review only

generic QoL at baseline in those patients who remained on the WL and who did not go on to receive a transplant disappeared, but the sample size was reduced by 16 participants. There were no meaningful differences in the outcomes at 12m follow-up in the analyses that did and did not control for these factors, so reported findings do not control for obesity and diabetes.

Table 2 shows the correlations between the main outcome variables at 12m follow-up. Table 3 shows the cross-sectional differences at one-year post-recruitment/post-transplant between those who remained on the WL for a transplant (n=98), and those who received a DD (n=145) or LD transplant (n=120). Participants on the WL reported significantly worse scores on all PROMs at one year compared with the two transplant groups (all p<0.001). LD recipients reported better generic QoL, renal-dependent QoL, well-being, and treatment satisfaction (all p<0.05) than DD recipients. These analyses suggest that LD recipients have better outcomes than DD recipients post-transplant, but do not consider baseline differences in PROMs pre-transplant.

Table 4 and Figure 2 depict the prospective analyses comparing over time, those who remained on the WL (n=98) and those who were recruited as WL patients, but who subsequently received a LD (n=26) or DD transplant (n=41). There were significant interaction effects between the three groups and from recruitment to one-year post-recruitment/post-transplant for all PROMs. At recruitment, WL patients who did not go on to receive a transplant reported worse generic QoL than those who went on to receive DD transplants (p=0.028). Patients who remained on the WL reported worsening EQ-VAS health ratings (p=0.004), utility values (p<0.001), and well-being (p<0.001) from recruitment to one year, but generic QoL, renal-dependent QoL, and treatment satisfaction remained stable over time. In contrast, DD recipients reported improvements in EQ-VAS health ratings (p=0.017), renal-dependent QoL (p=0.011), and RTSQs scores (p<0.001), but no change in utility values (p=0.450), well-being (p=0.380), or generic QoL (p=0.200) from pre- to post-transplant. LD recipients reported improvements in all outcomes post-transplant (p<0.01). Though LD recipients reported greater treatment satisfaction than DD recipients (p=0.038), the transplant groups did not differ on any other PROMs post-transplant. Patients still on the WL reported worse scores on all

For peer review only - http://bmjopen.bmj.com/site/about/guidelines.xhtml

PROMs at one year compared with both transplant groups (p<0.001). The effect sizes ranged from small to moderate (Table 4; partial η^2 = 0.10-0.27). Sixteen percent of DD recipients and 5% of LD recipients reported worse generic QoL post-transplant. In contrast, 7% of DD recipients and no LD recipients reported more negatively impacted AWI scores from pre- to post-transplant. Similarly, 8% of DD recipients and no LD recipients reported worse health status as measured by the EQ-VAS from pre-to post-transplant.

Using the treatment satisfaction scores from the RTSQs at recruitment as a further covariate in analyses, both DD (M=29.6, SE=2.7) and LD recipient groups (M=31.8, SE=3.4) reported greater satisfaction with renal treatment at one year versus previous treatment than those still on the WL (M=14.4, SE=1.9; $F_{(1, 97)}$= 16.1, p<0.001; partial η^2 = 0.25; Figure 3).

<u>Qualitative findings</u>

Three themes were identified from the qualitative interviews; participants discussed the positive impact of transplantation, the impact of expectations on the ability to cope post-transplant, and their feelings towards donors. Illustrative examples of quotations can be seen in Table 5. Overall, participants viewed their transplant as a way to return to “normal”. Both transplant groups reported physical improvements, reduced dietary and fluid restrictions, and improved lifestyle post-transplant. Many DD recipients had not yet returned to work, but some LD recipients reported improvements related to their employment, due to their increased physical ability.

Patient expectations of transplantation had a strong influence on whether or not patients felt their transplant was successful. Many patients believed that once they received a transplant, they would return to the same level of physical health they experienced prior to their kidney problems. Six out of the 10 LD recipients reported ongoing physical problems at one-year post-transplant, such as reduced stamina, which they did not anticipate. This led to more difficulty adjusting post-transplant, and lower rates of treatment satisfaction. An unexpected side effect of transplantation included changes in physical appearance, such as scarring, bloating from the surgery,

For peer review only - http://bmjopen.bmj.com/site/about/guidelines.xhtml

or weight gain from medication. Patients who experienced these changes found it harder to cope, and their QoL was negatively impacted. Other unexpected experiences, such as difficulty adapting to drinking large quantities of fluids and relearning bladder control post-transplant, were issues that participants found particularly difficult.

Although all participants reported feeling immense gratitude to their donors, LD recipients worried about the health of their donors, leading to feelings of guilt up to one-year post-transplant. This was particularly evident in three recipients whose donors were their adult children. Continued worry about their donors meant that LD recipients were likely to report difficulties in coping post-transplant.

Discussion

Previous research has shown consistently that there are differences in the patient characteristics of DD and LD recipients.[13,14,40] Controlling for such factors, our cross-sectional analyses suggest that LD transplantation conveys an advantage over DD transplantation for QoL and treatment satisfaction. However, once baseline differences in factors such as age, education, and time on dialysis were controlled for (in the subset of patients with true baseline measures pre-transplant), DD and LD kidney transplant recipients are found to have similar outcomes post-transplant. The only significant difference between transplant groups with true baseline data was in treatment satisfaction one-year post-transplant with LD recipients having greater treatment satisfaction than DD recipients. Previous research showed that LD recipients reported greater improvements within the first few months post-transplant, but LD and DD recipients did not differ after longer intervals of time.[17] Although the improvements seen in the LD recipients is encouraging, our results suggest that they have worse baseline measures of treatment satisfaction, health status and generic QoL before transplant, so their improvements post-transplant are more evident. Caution should be exercised when interpreting studies that have not controlled for differences at recruitment and rely on cross-sectional follow-up data alone.

For peer review only - http://bmjopen.bmj.com/site/about/guidelines.xhtml

The qualitative interviews show that although participants report improvements to their QoL through a return to 'normal' and improvements in lifestyle, participants still experience ongoing negative impact. Although transplantation is considered a positive and successful treatment, it cannot minimise the negative impact of a renal condition completely. The findings reflect those of previous studies, which report that some transplant recipients find it difficult to adjust and cope post-transplant.[41-43] Differing themes emerged for the LD and DD recipients; Although slightly more LD recipients had returned to work by one year (5/10 LD vs 3/10 DD), they were more likely to mention differences in adapting post-transplant and feel that their positive expectations for recovery and return to work were not realised. Most LD recipients were not on dialysis before they received their transplant, so the benefits they perceived from transplantation were fewer than those who have experienced dialysis. It is important to manage patient expectations (e.g. recovery times) to avoid disappointment and promote effective coping with physical and psychosocial changes following transplant. A small number of LD recipients reported experiencing feelings of guilt about the risks to their donors.[13,14] Further support may be required for those considering such a transplant, to help anticipate and manage the potential for the feelings of guilt which may include deciding to decline the offer. The impact of transplantation on QoL and other PROMs on both recipients and their donors need to be considered, especially when assessing the comparative benefit of LD versus DD transplantation.

In line with our hypotheses, WL patients reported worse health and well-being over time. This may in part be due to the fact that almost 18% of those on the WL were not yet on dialysis when recruited to the study but all required dialysis one year later. Some patients may find that their health improves once they commence dialysis, but on average health status declined. However, QoL and treatment satisfaction scores did not change over the course of 12 months in those on the WL despite their deteriorating health status and well-being. This study cannot explain in detail the whole experience of patients on the WL for a transplant, and due to the relatively small sample size and small effect sizes for some of the outcomes, some of the results may not have been adequately

For peer review only - http://bmjopen.bmj.com/site/about/guidelines.xhtml

powered. The lack of differences between groups over time therefore needs to be considered with caution. Despite this, the results highlight differences between health status and QoL.[20] Quality of life can be affected by multiple aspects of people's lives with their health being just one aspect, so it is important to distinguish between measures of genuine QoL and health status when selecting and interpreting the results from PROMs. This is particularly the case where the treatment for long-term conditions such as CKD may benefit health status but may impair QoL.

There are some limitations to this study. The sample of transplant participants with pre-transplant data was relatively small (n=67), as they were part of secondary analyses, some of which may be underpowered, so caution needs to exercised when drawing conclusions. However, the findings provide insight into how QoL and other patient-reported outcomes change over time in transplant recipients. Only those fluent in English were recruited to ensure that participants could complete the questionnaires: cost of linguistic validation for all the PROMs to be translated into other languages exceeded the ATTOM programme's resources. This meant that, for example, few people of South Asian descent took part in our sub-study, despite the fact that approximately 9.4% of transplant recipients recruited to the larger ATTOM research programme were of Asian ethnicity (with the great majority being of South Asian descent). Those interviewed for the qualitative interviews were mainly White. Therefore, the sample is less ethnically diverse than the UK renal population as a whole. Patients who responded at one year were more likely to be older, White, and have better health ratings at recruitment, so our findings may not be generalizable to younger non-White patients, and may give a more positive view of changes in health over time than if those with worse baseline health status were included. Ten patients were known to have died during the study period and no one reported a failed transplant during data collection, limiting the scope of the findings to those who remained healthy enough to be on the WL, and those with a functioning graft. Despite this, participants to our PROMs sub-study were recruited as part of ATTOM from renal units across the UK, reflecting the full ATTOM sample in terms of age, sex, previous transplant, and rate of pre-emptive transplantation. Most patients had not returned to work at follow-up; those who had

For peer review only - http://bmjopen.bmj.com/site/about/guidelines.xhtml

returned to work had varying levels of QoL compared to those who had yet to return to work. The long-term impact of taking immunosuppressants, including the increased risk of cancer and other diseases, may not yet be reflected in participants' QoL and other PROMs within the first year of having a transplant. Longer follow-up, therefore, may be required to show any differences across transplant groups, and the long-term impact of immunosuppressant medication on QoL. The focus of this study was on PROMs, but medical variables such as the number of complications post-transplant which may impact on QoL, were not assessed. Nevertheless, this is one of the few studies that provide any pre-transplant data, allowing us to examine changes over time in transplant recipients and, importantly, to control for baseline differences in PROMs. Additionally, the inclusion of WL patients along with recipients of LD and DD in analyses which controlled for group differences, allowed for direct comparison of outcomes for transplant recipients and those still on the WL. The paper highlights the importance of measuring and controlling for pre-existing differences between groups. A strength of this study is that it included both qualitative and quantitative data, which allowed for further understanding of the subjective experience of transplantation. It also shows that examining a range of PROMs provides a much better understanding of the experience of waiting for or receiving a transplant than is provided by health-status measures alone.

These findings have implications for clinical practice including future monitoring of patient-reported outcomes. Both transplant groups reported better outcomes than those still on the WL for a transplant, but there were few differences in PROMs between groups post-transplant after adjustment for potential confounders. Living donation has certain medical advantages, including that recipients do not have to wait as long for the transplant, but it is also important to acknowledge that living donation doesn't just affect recipients and their donors physically. Discussion is needed about more than just the medical benefits for the recipient and the medical impact on the donor; PROMs need to be better integrated into the information provided to recipients and donors when transplantation is discussed as a treatment option. Knowing that not all patients report improvements in PROMs and why may help future patients avoid disappointment. Routine

For peer review only - http://bmjopen.bmj.com/site/about/guidelines.xhtml

collection of PROMs data at time of wait-listing and periodically thereafter is required so that a more accurate picture of changes pre- to post-transplant can be achieved from larger more representative datasets. Careful expectation management, information on recovery time, anticipated physical changes (including scar size) and side-effects may help avoid disappointment and ensure better informed decisions about transplantation options. Nevertheless, both transplant types were, overall, more beneficial than remaining on the WL and may be considered similarly advantageous in improving QoL and other patient-reported outcomes.

For peer review only

For peer review only - http://bmjopen.bmj.com/site/about/guidelines.xhtml

Contributorship statement:

AG contributed to the design of the qualitative study, developed the interview schedule, conducted the interviews, developed the coding framework, coded and analysed the quantitative and qualitative data, drafted the manuscript, and edited and approved the final submission. JB managed telephone and postal data collection, coded qualitative data, provided feedback on initial drafts of the manuscript, and edited and approved the final submission. MC contributed to the design of the qualitative study, development of the interview schedule and coding framework, provided feedback on initial drafts of the manuscript, and edited and approved the final submission. HD contributed to the design of the qualitative study, development of the interview schedule, and edited and approved the final submission. RJ, GCO, RR, CRVT, PR, WM, JF, and CD contributed to the design, organization and conduct of the wider ATTOM programme including data collection for this sub-study, and provided feedback on the interview schedule, and edited and approved the final submission. JAB, RR, GO, CJEW, CJVT, CD and JF conceived and designed the ATTOM programme, contributed to the design of the present studies, provided feedback on the interview schedule, and edited and approved the final submission. CB contributed to the design of the quantitative and qualitative studies, analysis planning and interpretation of results, development of the interview schedule and coding framework, edited early drafts of the manuscript, and edited and approved the final submission. AG and CB act as guarantor, and accept full responsibility for the work and/or the conduct of the study, had access to the data, and controlled the decision to publish. The corresponding author attests that all listed authors meet authorship criteria and that no others meeting the criteria have been omitted. We thank the patient advisers for their contributions to the planning of ATTOM and discussions of findings.

Competing interests: All authors completed the ICMJE uniform disclosure form at www.icmje.org/coi_disclosure.pdf. Prof Watson reports personal fees from Glaxo Smith Kline outside the submitted work. Prof. Clare Bradley reports grants from NIHR during the conduct of the

For peer review only - http://bmjopen.bmj.com/site/about/guidelines.xhtml

study, and grants from NIHR and GlaxoSmithKline/ViiV Healthcare, outside the submitted work. CB is the majority shareholder in a company, Health Psychology Research Ltd, which licenses her patient-reported outcome measures, for others to use. These questionnaires include the RDQoL, RTSQ, and W-BQ used in the ATTOM programme. CB owns the copyright in all of these instruments and when they are licensed for use by commercial companies in their clinical trials, receives royalties. All other authors declared no competing interests.

The results presented in this paper have not been published previously in whole or part, except in abstract format.

Funding: This article presents independent research funded by the National Institute for Health Research (NIHR) under the Programme Grants for Applied Research scheme (RP-PG-0109-10116). The views expressed are those of the authors and not necessarily those of the NHS, the NIHR or the Department of Health.

Data sharing: Data is available upon reasonable request.

Ethical approval: IRAS project ID: 68259. REC reference 11/EE/0120

AG and CB affirm that the manuscript is an honest, accurate, and transparent account of the study being reported; that no important aspects of the study have been omitted; and that any discrepancies from the study as originally planned (and, if relevant, registered) have been explained.

The Corresponding author has the right to grant on behalf of all authors and does grant on behalf of all authors, a worldwide licence to the Publishers and its licensees in perpetuity, in all forms, formats and media (whether known now or created in the future), to i) publish, reproduce, distribute, display and store the Contribution, ii) translate the Contribution into other languages, create adaptations,

For peer review only - http://bmjopen.bmj.com/site/about/guidelines.xhtml

reprints, include within collections and create summaries, extracts and/or, abstracts of the Contribution, iii) create any other derivative work(s) based on the Contribution, iv) to exploit all subsidiary rights in the Contribution, v) the inclusion of electronic links from the Contribution to third party material where-ever it may be located; and, vi) licence any third party to do any or all of the above.

For peer review only

For peer review only - http://bmjopen.bmj.com/site/about/guidelines.xhtml

References

1. Black N. Patient reported outcome measures could help transform healthcare. *BMJ* 2013; 346: 1167 https://doi.org/10.1136/bmj.f167
2. Tajima, R., Kondo, M., Kai, H. et al. Measurement of health-related quality of life in patients with chronic kidney disease in Japan with EuroQol (EQ-5D). *Clin Exp Nephrol* 2010; 14: 340–348 https://doi.org/10.1007/s10157-010-0304-1
3. Boyce MB, Browne JP, Greenhalgh J. The experiences of professionals with using information from patient-reported outcome measures to improve the quality of healthcare: A systematic review of qualitative research. *BMJ Qual Saf* 2014; 23:508-518.
4. Coulter A. Measuring what matter to patients. *BMJ* 2017; 356 doi: https://doi.org/10.1136/bmj.j816
5. MacNeill SJ, Ford D. UK Renal Registry 19th Annual Report: Chapter 2 UK Renal Replacement Therapy Prevalence in 2015: National and Centre-specific Analyses/ Nephron 2017; 137(suppl):45-72.
6. Kidney Advisory Group on behalf of NHSBT. Kidney Transplantation: Deceased Donor Organ Allocation. 2019. Policy POL186/10. Available from https://nhsbtdbe.blob.core.windows.net/umbraco-assets-corp/16915/kidney-allocation-policy-pol186.pdf
7. NHS Blood and Transplant. Annual Report on Kidney Transplantation 2016/17. 2017. Available from https://nhsbtdbe.blob.core.windows.net/umbraco-assets-corp/4607/kidney-annual-report-2016-17.pdf. [Accessed 23rd October 2017].
8. Nemati E, Einollahi B, Pezeshki ML, Porfarziani V, Fattahi MR. Does kidney transplantation with deceased or living donor affect graft survival? *Nephrol Urol Mon* 2014; 6(4):e12182. doi: 10.5812/numonthly.12182
9. Gjertson DW, Cecka JM. Living unrelated donor kidney transplantation. *Kidney International* 2000; 58:491-499.

For peer review only - http://bmjopen.bmj.com/site/about/guidelines.xhtml

For peer review only

10. Wu DA, Robb ML, Watson CJE, et al. Barriers to living donor kidney transplantation in the United Kingdom: a national observational study. *Nephrol Dial Transplant* 2017; 1;32(5):890-900.

11. Molmenti EP, Alex A, Rosen L, et al. Recipient criteria predictive of graft failure in kidney transplantation. *Int J Angiol* 2016; 25:29-38.

12. Gozdowska J, Zatorski M, Torchalla P, et al. Living-donor versus deceased-donor kidney transplantation: Comparison of psychosocial consequences for recipients. *Transplant Proc* 2016; 38:1498-1505.

13. Ware JE, Sherbourne CD. The MOS 36-item short-form health survey (SF-36). *Med Care* 1992; 30:473-483.

14. Griva K, Ziegelman JP, Thompson D, et al. Quality of life and emotional responses in cadaver and living related renal transplant recipients. *Nephrol Dial Transplant* 2002; 17: 2204-2211.

15. Zimmermann T, Pabst S, Bertram A, Schiffer M, de Zwaan M. Differences in emotional responses in living and deceased donor kidney transplant patients. *Clin Kidney J* 2016; 9(3): 503-509.

16. Costa-Requena G, Cantarell MC, Moreso F, Parramon G, Seron D. Calidad de vida relacionada con la salud en el trasplante renal: seguimiento longitudinal a 2 años. *Med Clin (Barc)* 2017; 49:114-118.

17. Griva K, Stygall J, Ng JH, Davenport A, Harrison MJ, Newman S. Prospective changes in health-related quality of life and emotional outcomes in kidney transplantation over 6 years. *J Transplant* 2011; 671571.

18. Lønning K, Heldal K, Bernklev T, et al. Improved health-related quality of life in older kidney recipients 1 year after transplantation. *Transplant Direct* 2018; 4: e351; doi: 10.1097/TXD.0000000000000770

19. Suzuki A, Kenmochi T, Maruyama M, et al. Changes in quality of life in deceased versus living-donor kidney transplantations. *Transplant Proc* 2012; 44:287-289.

For peer review only

For peer review only - http://bmjopen.bmj.com/site/about/guidelines.xhtml

20. Bradley C. Importance of differentiating health status from quality of life. The Lancet 2001; 357: 7-8. doi: 10.1016/S0140-6736(00)03562-5

21. Bradley C. Design of a Renal-Dependent Individualized Quality of Life Questionnaire. *Adv Perit Dial* 1997; 13: 116-120.

22. Bradley C, Todd C, Gorton T, et al. The development of an individualised measure of perceived impact of diabetes on quality of life: The ADDQoL. *Qual Life Res* 1999; 8(1-2): 79-91.doi:10.1023/A:1026485130100.

23. McGee HM, O'Boyle CA, Hickey A, O'Malley K, Joyce CRB. Assessing the quality of life of the individual: the SEIQoL with a healthy and a gastroenterology unit population. *Psychol Med* 1991; 21: 749-59.

24. Bradley C, Eschwège E, de Pablos-Velasco P, Parhofer KG, Simon D, Vandenberghe H et al. Predictors of quality of life and other patient-reported outcomes in the PANORAMA multinational study of people with type 2 diabetes. *Diabetes Care* 2018; 41(2):267-276.

25. Oniscu GC, Ravanan R, Wu D, et al. Challenges and opportunities in renal transplantation: Access to Renal Transplantation and Transplant Outcome Measures (ATTOM) study. *BMJ Open* 2016; 6:e010377.

26. Ravanan R, Udayaraj U, Ansell D, et al. Variation between centres in access to renal transplantation in UK: longitudinal cohort study. *BMJ* 2010; 341: c3451

27. Oniscu GC, Schalkwijk AAH, Johnson RJ, et al. Equity of access to renal transplant waiting list and renal transplantation in Scotland: cohort study. *BMJ* 2003; 327:1261.

28. Louis ON, Sankar P, Ubel PA. Kidney transplant candidates' views of the transplant allocation system. *J Gen Intern Med* 1997; 12: 478-484.

29. Wee HL, Tan CE, Goh SY, Li SC. Usefulness of the Audit of Diabetes-Dependent Quality-of-Life (ADDQoL) Questionnaire in patients with diabetes in a multi-ethnic Asian country. *Pharmacoeconomics* 2006; 24(7): 673-682.

For peer review only - http://bmjopen.bmj.com/site/about/guidelines.xhtml

30. Riazi A, Bradley C, Barendse S, Ishii H. Development of the Well-being Questionnaire short-form in Japanese: The W-BQ12. *Health Qual Life Out* 2006; 4:40. doi: 1 0.1186/1477-7525-4-40

31. Herdman M, Gudex C, Lloyd A, et al. Development and preliminary testing of the new five-level version of the EQ-5D (EQ-5D-5L). *Qual Life Res* 2011; 20(10):1717-1736. doi: 10.1007/s11136-011-9903-x

32. Feng Y, Devlin N, Shah K, et al. New methods for modelling EQ-5D-5L value sets: An application to English data. Office of Health Economics, Research Paper 16/2 January 2016; London. Available from https://www.ohe.org/publications/new-methods-modelling-eq-5d-5l-value-sets-application-english-data. [Accessed 20th April 2017].

33. Barendse SM, Speight J, Bradley C. The Renal Treatment Satisfaction Questionnaire (RTSQ): A measure of satisfaction with treatment for chronic kidney failure. *Am J Kidney Dis* 2005; 45: 572-579.

34. Howorka K, Pumprla J, Schlusche C, et al. Dealing with ceiling baseline treatment satisfaction level in patients with diabetes under flexible, functional insulin treatment: Assessment of improvements in treatment satisfaction with a new insulin analogue. *Qual Life Res* 2000; 9: 915-930.

35. Bradley C. The Diabetes Treatment Satisfaction Questionnaire: DTSQ. In Bradley C. (Ed). Handbook of Psychology and Diabetes: A guide to psychological measurement in diabetes research and practice. Chur Switzerland: Harwood Academic Publishers, 1994; 111-132.

36. Field A. Discovering statistics. London. Sage 2009.

37. O'Brien BC, Harris IB, Beckman TJ, Reed DA, Cook DA. Standards for reporting qualitative research: a synthesis of recommendations. *Acad Med* 2014; 89(9):1245-1251.

38. Braun V, Clarke V. Using thematic analysis in psychology. *Qual Res Psychol* 2006; 3:77-101.

For peer review only - http://bmjopen.bmj.com/site/about/guidelines.xhtml

39. Wu DA, Robb ML, Forsythe JLR, Bradley C, Cairns J, Draper H, et al. Recipient comorbidity and survival outcomes after kidney transplantation: a UK-wide prospective cohort study. *Transplantation* 2019, Aug 19. doi: 10.1097/TP.0000000000002931.

40. Guimarães J, Araújo AM, Santos F, Nunes CS, Casal M. Living-donor and deceased-donor renal transplantation: Differences in early outcome - A single-center experience. *Transplantation Proceedings* 2015; 47:958-962.

41. Schipper K, Abma TA, Koops C, et al. Sweet and sour after renal transplantation: A qualitative study about the positive and negative consequences of renal transplantation. *Br J Health Psychol* 2014; 19: 580-591.

42. Fallon M, Gould D, Wainwright SP. Stress and quality of life in the renal transplant patient: A preliminary investigation. *J Adv Nurs* 1997; 25: 562-570.

43. Baines LS, Joseph JT, Jindal RM. Emotional issues after kidney transplantation: A prospective psychotherapeutic study. *Clin Transplant* 2002; 16: 455-460.

Table 1. Summary of baseline demographic characteristics of groups at recruitment.

	Wait-list group (*N*=165)	DD recipients (*N*=104)	LD recipients (*N*=94)	
Variable	*M (SE)*	*M (SE)*	*M (SE)*	*p*
Age[a] in years	50.7 (1.6)	51.1 (1.3)	43.9 (1.4)	<0.001
Time on waiting list in days	980 (124.9)	953 (284.9)	990 (414.4)*	0.995
Sociodemographic variables	*N (%)*	*N (%)*	*N (%)*	*p*
Age[a]				<0.001
18-34	25 (15.1)	12 (11.5)	29 (30.9)	
35-49	46 (27.9)	32 (30.8)	32 (34.0)	
50-64	66 (40.0)	41 (39.4)	27 (28.7)	
65-75	28 (17.0)	19 (18.3)	6 (6.4)	
Sex: Female	59 (35.8)	40 (38.5)	34 (36.2)	0.949
Ethnicity				0.411

For peer review only - http://bmjopen.bmj.com/site/about/guidelines.xhtml

White	134 (81.2)	84 (80.8)	85 (90.4)	
Black	15 (9.1)	10 (9.6)	5 (5.3)	
Asian	1 (0.6)	1 (1.0)	0 (0.0)	
Chinese	13 (7.9)	7 (6.7)	3 (3.2)	
Mixed	2 (1.2)	2 (1.9)	1 (1.1)	
Marital status				0.462
Single	32 (19.4)	20 (19.2)	27 (28.7)	
Married/Living with partner	98 (59.4)	63 (60.6)	60 (63.8)	
Divorced/separated/widowed	35 (21.2)	121 (20.2)	7 (7.5)	
Education				0.166
No qualifications[b]	32 (19.4)	16 (15.4)	8 (8.5)	0.033
Basic (GCSE/A level/NVQ 1-3)	94 (57.0)	66 (63.5)	68 (72.3)	
Higher (Degree/higher degree/NVQ 4-5)	39 (23.6)	22 (21.1)	18 (19.2)	
Car ownership: yes	134 (81.2)	89 (85.6)	90 (95.7)	<0.000
Clinical Variables				
Primary renal diagnosis				0.515
Diabetes: Type 1 or 2	9 (5.5)	11 (10.6)	5 (5.3)	
Glomerulonephritis	50 (30.3)	32 (30.8)	42 (44.7)	
Interstitial nephritis/pyelonephritis	21 (12.7)	13 (12.5)	16 (17.0)	
Hypertension/large vessel disease	10 (6.1)	8 (7.7)	0 (0.0)	
Cystic/hereditary/congenital disease	47 (28.4)	19 (18.3)	14 (14.9)	
Other conditions	28 (17.0)	21 (20.1)	17 (18.1)	
Comorbid conditions				
Diabetes	20 (12.2)	16 (15.4)	8 (8.5)	<0.005
Heart disease	19 (11.6)	6 (5.8)	7 (7.4)	0.006
Heart failure	6 (3.7)	4 (3.8)	0 (0.0)	0.305
Liver disease	4 (2.4)	3 (2.9)	1 (1.1)	0.299
Mental health problems	14 (8.5)	2 (1.9)	5 (5.3)	0.022

For peer review only - http://bmjopen.bmj.com/site/about/guidelines.xhtml

Obesity (BMI >30)	32 (19.5)	22 (21.2)	14 (14.9)	0.600
Previous Renal replacement therapy				<0.001
No dialysis[c]	29 (17.6)	13 (12.5)	43 (45.7)	
Peritoneal dialysis (PD)	27 (16.4)	23 (22.1)	14 (14.9)	
Haemodialysis (HD)[d]	109 (66.0)	68 (65.4)	37 (39.4)	
Central venous catheter (CVC)	35 (53.1)	31 (55.6)	21 (56.8)	
Arteriovenous fistula (AVF)	22 (33.3)	28 (41.2)	15 (40.5)	
Unknown	9 (13.6)	9 (13.2)	1 (2.7)	
Previous transplant failure[e]	47 (28.5)	12 (11.5)	13 (13.8)	0.001
Highly sensitized: yes	8 (4.9)	9 (8.7)	8 (8.5)	0.842
Donor related to patient: yes	-	-	56 (59.6)	
Induction suppression				0.116
None	-	16 (15.4)	19 (20.2)	
Anti-thymocyte Globulin (ATG)	-	7 (6.7)	1 (1.1)	
Basiliximab	-	66 (63.5)	53 (56.4)	
Campath	-	15 (14.4)	21 (1.1)	
Calcineurin inhibitor (CNI) maintenance				0.073
Tacrolimus	-	98 (94.2)	93 (98.9)	
Ciclosporine	-	6 (5.8)	1 (1.1)	
Anti-proliferative drugs				0.341
None	-	13 (12.5)	8 (8.5)	
Mycophenolate	-	70 (67.3)	54 (57.4)	
CellCept	-	8 (7.7)	16 (17.0)	
Myfortic	-	7 (6.7)	10 (10.6)	
Azathioprine	-	6 (5.8)	6 (6.4)	
Steroid maintenance plan				0.193
None	-	9 (8.7)	12 (12.8)	
Withdraw within 3 months	-	18 (17.3)	24 (25.5)	

For peer review only

For peer review only - http://bmjopen.bmj.com/site/about/guidelines.xhtml

Long-term use	-	77 (74.0)	58 (61.7)

Notes. *Figures here reflect only those who were on the waiting list (WL) for a DD transplant before receiving a LD transplant (n=16). [a]LD recipients were younger. [b]Fewer LD recipients reported no educational qualifications. [c] A larger proportion of LD recipients were transplanted pre-emptively. [d]More DD recipients and WL patients reported being on HD prior to transplantation/listing. [e]WL patients reported more previous transplant failures.

For peer review only

Table 2. Correlations between outcome measures 12-month follow-up for all participants.

	Variable	*1*	*2*	*3*	*4*	*5*	*6*
**1**	Generic QoL	-					
**2**	Renal-dependent QoL	0.499***	-				
**3**	Well-being	0.740***	0.551***	-			
**4**	Health status (EQ-VAS)	0.725***	0.415***	0.652***	-		
**5**	Health-utility values	0.674***	0.444***	0.678***	0.612***	-	
**6**	Renal treatment satisfaction (RTSQs)	0.545***	0.420***	0.521***	0.454***	0.364***	-
**7**	Change in treatment satisfaction (RTSQc)	0.282***	0.134*	0.292***	0.293***	0.125*	0.617***

Note. *p<0.05, **p<0.01, ***p<0.001

For peer review only - http://bmjopen.bmj.com/site/about/guidelines.xhtml

Table 3. Summary of adjusted means, standard errors, and main effects examining cross-sectional differences in groups in patient-reported outcomes at one year, controlling for age, previous renal replacement therapy (RRT), previous experience of a transplant, education, car ownership, and mental health problems.

	Wait-list group		***DD Transplant***		***LD Transplant***					
	M (SE)	*CI*	*M (SE)*	*CI*	*M (SE)*	*CI*	*F*	*df*	*p*	Partial η^2
Generic QoL[a,b]	0.4 (0.1)	0.1-0.7	1.4 (0.1)	1.1-1.6	1.8 (0.1)	1.5-1.9	27.6	266	<0.001	0.20
Renal-dependent QoL [a,b]	-4.4 (0.3)	-4.9 to -3.9	-2.7 (0.2)	-3.1 to -2.4	-1.9 (0.2)	-2.3 to -1.5	27.2	266	<0.001	0.18
Well-being[a,]	19.0 (0.9)	14.3-20.8	24.9 (0.7)	23.7-26.3	27.4 (0.8)	25.8-28.9	24.7	265	<0.001	0.17
Health status (EQ-VAS)[a]	62.4 (2.2)	58.0-66.8	77.9 (1.6)	74.8-81.2	82.4(1.9)	78.7-86.1	24.1	263	<0.001	0.18
Health-utility values[a]	0.7 (0.1)	0·6-0.8	0.8 (0.1)	0.8-0.9	0.9 (0.1)	0.8-0.9	13.1	259	<0.001	0.11
Renal treatment satisfaction (RTSQs) [a,b]	58.6 (1.3)	56.1-61.1	67.4 (0.9)	65.5-69.2	72.6 (1.1)	70.5-74.7	34.3	265	<0.001	0.21
Change in treatment satisfaction (RTSQc)[a]	14.1 (1.7)	10.8-17.4	29.2 (1.3)	26.7-31.7	30.9 (1.4)	28.2-33.8	33.6	253	<0.001	0.22

Note. DD = deceased-donor transplant, LD = living-donor transplant. *M*=mean, *SE*=standard error. EQ-VAS=Visual Analogue Scale; RTSQs=Renal Treatment Satisfaction Questionnaire status version; RTSQc=Renal Treatment Satisfaction Questionnaire change version. Partial η^2 is a measure of effect size that

For peer review only - http://bmjopen.bmj.com/site/about/guidelines.xhtml

measures the proportion of the total variance in a dependent variable that is associated with the membership of different groups defined by an independent variable, in which the effects of other independent variables and interactions are partialled out. Partial η^2 is seen as giving small (0.01) medium (0.09), or large (0.25) effect sizes.

[a]Wait-list (WL) group reported worse outcomes compared with DD or LD recipients (*ps*<0.001).

[b]LD recipients reported better scores than DD recipients (*ps*<0.05).

For peer review only

For peer review only - http://bmjopen.bmj.com/site/about/guidelines.xhtml

Table 4. Summary of adjusted means, standard errors, and interaction effects examining differences in those who remained on the waiting list and those who subsequently had a LD or DD transplant, in patient-reported outcomes from pre- to 12-months post-transplant/recruitment to 12 months, controlling for age, previous renal replacement therapy (RRT), previous experience of a transplant, education, car ownership, and mental health problems.

		Generic QoL	Renal-dependent QoL	Well-being	Health status EQ-VAS scores	Health-utility values	Renal treatment satisfaction (RTSQs)
Recruitment							
WL	*M (SE)*	0.39 (0.15)	-4.29 (0.27)	22.94 (0.78)	69.32 (2.63)	0.78 (0.03)	58.43 (1.76)
	CI	0.10-0.69	-4.82 to -3.75	21.40-24.48	64.10-74.53	0.73-0.83	54.94-61.93
DD	*M (SE)*	1.11 (0.21)	-3.98 (0.39)	23.18 (1.04)	67.42 (3.50)	086 (0.03)	60.10 (2.42)
	CI	0.69-1.52	-4.74 to -3.12	21.11-25.25	60.48-74.36	0.79-0.92	55.29-64.91
LD	*M (SE)*	0.77 (0.27)	-4.08 (0.49)	22.37 (1.40)	61.10 (4.27)	0.86 (0.04)	54.76 (3.15)
	CI	0.23-1.31	-5.06 to -2.15	19.59-25.15	51.78-70.42	0.77-0.94	48.50-61.01
12m post-recruitment / 12m post-transplant							
WL	*M (SE)*	0.39 (0.14)	-4.45 (0.27)	18.95 (0.99)	61.89 (2.39)	0.69 (0.03)	59.11 (1.45)
	CI	0.11-0.68	-4.99 to -3.91	16.99-20.92	57.16-66.63	0.63-0.75	56.24-61.99
DD	*M (SE)*	1.30 (0.20)	-2.92 (0.39)	24.45 (1.33)	77.19 (3.18)	0.85 (0.04)	66.07 (1.99)
	CI	0.90-1.69	-3.68 to -2.15	21.81-27.09	70.88-83.49	0.76-0.93	62.12-70.03

For peer review only - http://bmjopen.bmj.com/site/about/guidelines.xhtml

LD	*M (SE)*	1.77 (0.26)	-2.41 (0.49)	27.30 (1.79)	83.90 (4.27)	0.93 (0.05)	74.07 (2.59)
	CI	1.25-2.28	-3.39 to –1.43	23.74-30.84	75.43-92.37	0.82-1.03	68.93-79.21
	F	5.91	12.08	14.43	20.63	8.04	15.25
	df	103	102	111	110	108	101
	p	0.004	<0.001	<0.001	<0.001	0.001	<0.001
	partial η^2	0.10	0.19	0.21	0.27	0.13	0.23

Note. WL= those who remained on the waiting list from recruitment to 12m post-recruitment, DD=those recruited as WL patients, but who subsequently had a deceased-donor transplant, LD= those recruited as WL patients, but who subsequently had a living-donor transplant, *M*=mean, *SE*=standard error, CI=confidence interval: upper and lower limits. EQ-VAS=Visual Analogue Scale; RTSQs=Renal Treatment Satisfaction Questionnaire status version.

For peer review only - http://bmjopen.bmj.com/site/about/guidelines.xhtml

Table 5. Summary of qualitative themes with illustrative quotations.

Theme	Illustrative quotations
Positive impact of transplantation	**Physical changes:**<br>▪ Uh, I feel a hell of a lot better. I was terribly, terribly ill, and I even look like me now. I didn't look like me for quite a long time. I had yellow eyes and grey skin. There's no doubt at all that it's made a massive positive difference. *Woman, non-related-LD transplant following HD.*<br>▪ My energy levels are amazing… my mates and that, they just couldn't believe it. They said 'god you look better than I do'. And I was full of life, I'm full of life. That's the sort of euphoria that you need, that gradually, very gradually subsided to normality I suppose. But I was, I was almost hyper. I felt so good. *Man, pre-emptive related-LD transplant.*<br>**Lifestyle:**<br>▪ Everything's gone right back to normal now it's fine… You go back to practically normal. *Man, DD transplant following HD.*<br>▪ I feel a bit normal again you know yeah *Woman, DD transplant following APD*.<br>▪ I'm back to how I was some years back. *Woman, pre-emptive non-related-LD transplant.*<br>▪ It gives you more freedom to, to actually live the life that I wanted to live, before dialysis… me and my wife are planning our honeymoon because I missed that because I just hit dialysis after my wedding. So now… after all these years on dialysis, we can plan for the future. *Man, DD transplant following HD.*<br>▪ Being able to work is massive for me and I can now work full-time and not only work full-time but own my own home which I wasn't able to do before. *Woman, non-related-LD transplant following HD.*<br>▪ I can just get up and do things without worrying… It is so much better than what it was before. Before I thought I was going to die, now ok I know I'm going to die but maybe hopefully in the future. So, I don't worry about the little things anymore. *Woman, DD transplant following HD.*<br>▪ I mean I can drink as much as I like now. That before you could only drink like a litre a day and that. *Woman, DD transplant following HD.*<br>▪ I mean I've gone back; I have a normal diet now. *Man, non-related-LD transplant following APD.*

For peer review only - http://bmjopen.bmj.com/site/about/guidelines.xhtml

Impact of expectations on ability to cope post-transplant	**Physical changes:**<br>▪ I have to drink like a fish. So, I'm all bloated … all I've got to show for (the transplant) is a huge stomach, because obviously they fitted a kidney in and the bag in my bladder so I look like I'm pregnant all the time… I'm a bit of a recluse actually. I just stay in all the time or if I was going out I can't, I can't wear anything fitted anymore because I look pregnant. Just, it's really, rubbish! Woman, *pre-emptive DD transplant.*<br>▪ I've lots of marks on my face (from a reaction to the medication) which isn't the greatest thing for confidence in the world. *Man, related-LD transplant following HD.*<br>▪ I'm on steroids so I have put on a bit of weight. *Man, pre-emptive DD transplant.*<br>▪ There's a thing when you have a transplant that you tend to pile it on, pile the weight on quite badly. *Woman,* non-*related LD transplant following HD.*<br>▪ You have to take a lot of water … after your transplant. You're supposed to take two litres every day…I find I'm running to the toilet a lot. It breaks your sleep at night, you're up maybe three times during the night and you're waking up and you've not really properly slept. *Man, pre-emptive related- LD transplant.*<br>▪ I hadn't used my bladder for seven years, and it was pretty darn painful (to use it again) and took a lot of running to the loo every five minutes. *Woman, DD transplant following HD.*<br>▪ Then oh god I was weeing for Britain! You know, it was, that was something I found very hard to get used to because as you know your bladder would have shrunk to nothing … when I had the transplant, I had to drink six litres. I found that quite hard. *Woman, DD transplant following HD.*<br>▪ When you're on dialysis you dream of being able to drink. Once you're told you have to drink its actually really difficult…. It's becomes normal not to drink anything and then all of a sudden, you're told to drink at least 3, 4 litres of water or fluid a day. *Woman, non-related-LD transplant following HD.*<br>▪ I'm still a little bit sort of behind where I would be but I've got like 30, I've got 35% function which is a little bit beyond where I need to be to be feeling properly sort of strong and active. But I've had a year of it being sort of, it's never been really bad but I've had a year of it being not quite where it needs to be so I'm a lot more tired than I'd like to be. *Man, DD transplant following HD.*

For peer review only - http://bmjopen.bmj.com/site/about/guidelines.xhtml

	▪ I've just not got the stamina I had before like you know. *Man, non-related-LD transplant following APD.*<br>▪ I know it's taken me a whole year to really be as I was before and I still feel in some ways I'm not quite back to what I was before. *Woman, pre-emptive non-related-LD.*<br>▪ My metabolism seems to have changed a great deal from the moment I had the transplant. *Woman, related-LD transplant following HD.*<br>▪ I've always wanted to try out cabin crewing before but now I can't even think of doing it because of my health conditions cos I can deteriorate if I start to fly. I have to be very cautious about what I want, you know like what I choose now, just to have to be very careful basically, even holidays. It's just very depressing. *Woman, DD transplant following PD.*
Feelings towards donor	**Gratitude:**<br>▪ I'm so grateful for having it. *Man, DD transplant following HD.*<br>▪ I'm very lucky to get the chance, due to my donor like; you know to get a chance of living again, and being without dialysis. *Woman, DD transplant following PD.*<br>▪ I mean I really, I'm incredibly, incredibly lucky I've got this person who I don't know, somewhere in the world, somewhere in the UK, I don't know where, to thank for this change of my, total change of my life. *Woman, non-related-LD transplant following HD.*<br>**Worry:**<br>▪ Psychologically I would rather have had a dead donor. For the simple reason that you know, it's difficult to deal with taking a part of your daughter's body out which she may need in later years. She was 27 at the time. So… it was very difficult, very difficult. *Man, pre-emptive related-LD transplant.*<br>▪ She's an only child and she has 2 children of her own and (it's) such a sacrifice and if anything had gone wrong or even now if anything happens to her and her other kidney, … I worry about her. *Woman, related-LD transplant following HD.*<br>▪ You worry about the future you know. My (donor) she's very young, she's all of her life and that you know. *Man, pre-emptive related-LD transplant.*

Note. DD=deceased donor, LD=living donor, HD=haemodialysis, PD=peritoneal dialysis, APD=automated peritoneal dialysis.

For peer review only

For peer review only

For peer review only - http://bmjopen.bmj.com/site/about/guidelines.xhtml

Figure 1. CONSORT diagram.

Note. WL= wait-list group, DD = deceased donor, LD = living donor.

Figure 2. Interaction graphs showing differences in outcomes at recruitment and at one-year post-transplant/post-recruitment in those who remained on the waiting list (WL) for a kidney transplant (*n*=98), or those who were recruited as WL patients and subsequently received a DD kidney transplant (*n*=41), or a LD kidney transplant (*n*=26). Adjusted scores shown, controlling for age, previous RRT, previous experience of a transplant, education, car ownership, and history of mental health problems. *$p<0.05$ WL vs LD/DD, **$p<0.01$ WL vs LD/DD, ***$p<0.001$ WL vs LD/DD. WL= wait-list group, DD = deceased donor, LD = living donor.

Figure 3. Bar graph showing differences in satisfaction with current renal treatment compared with previous renal treatment at one-year post-transplant/ post-recruitment in those who remained on the waiting list for a kidney transplant, or those who received a DD or LD kidney transplant after recruitment. Adjusted scores shown, controlling for age, previous RRT, previous experience of a transplant, education, car ownership, history of mental health problems, and RTSQs scores at recruitment. WL= wait-list group, DD = deceased donor, LD = living donor.

For peer review only

For peer review only - http://bmjopen.bmj.com/site/about/guidelines.xhtml

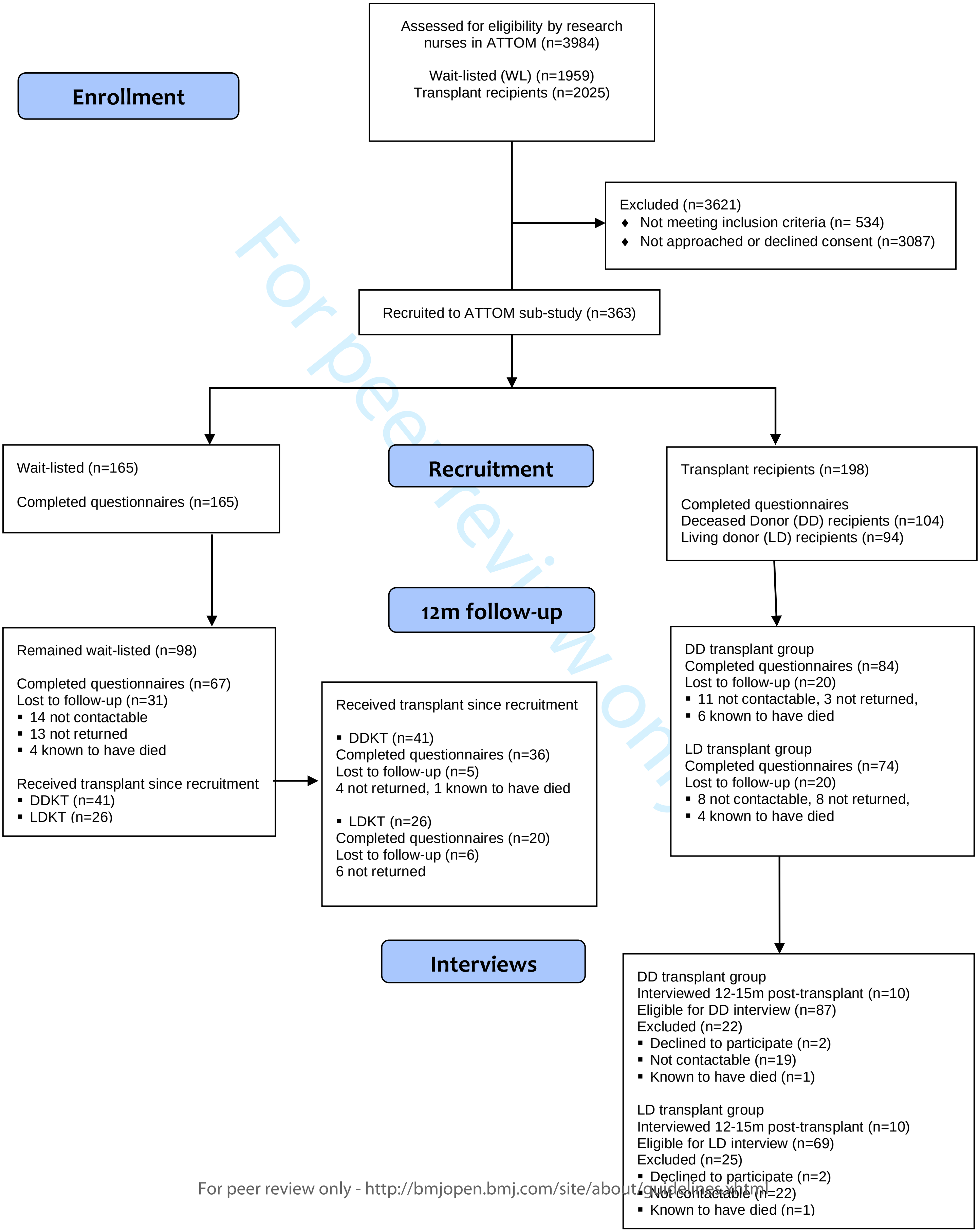

For peer review only

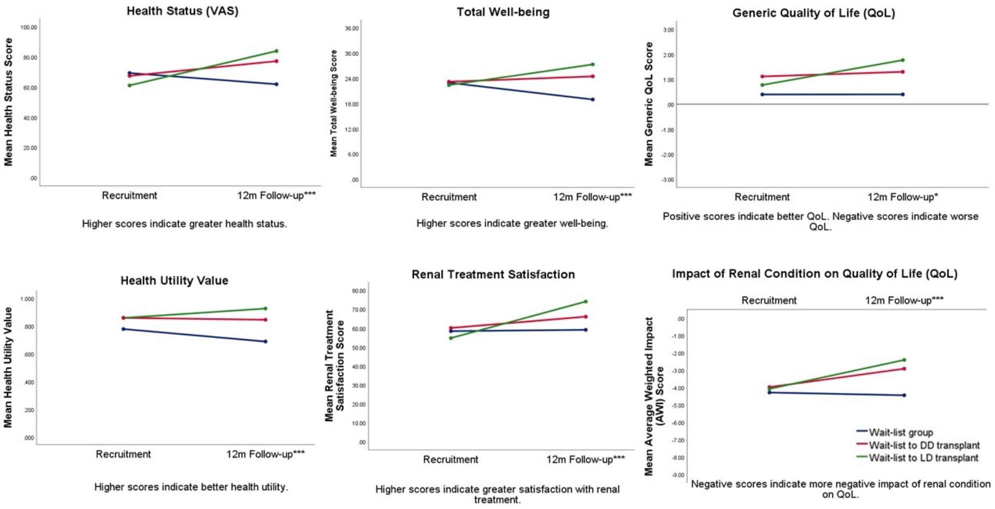

Figure 2. Interaction graphs showing differences in outcomes at recruitment and at one-year post-transplant/post-recruitment in those who remained on the waiting list (WL) for a kidney transplant (n=98), or those who were recruited as WL patients and subsequently received a DD kidney transplant (n=41), or a LD kidney transplant (n=26). Adjusted scores shown, controlling for age, previous RRT, previous experience of a transplant, education, car ownership, and history of mental health problems.

338x190mm (96 x 96 DPI)

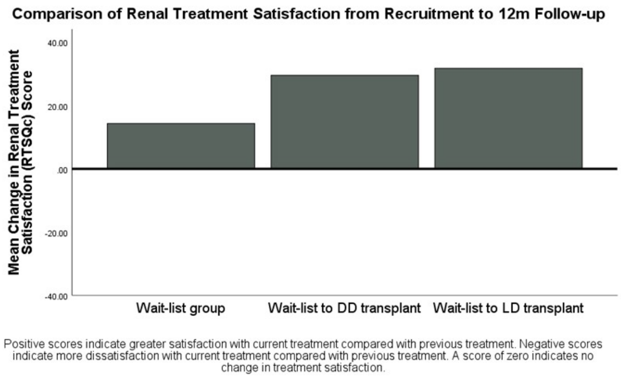

Figure 3. Bar graph showing differences in satisfaction with current renal treatment compared with previous renal treatment at one-year post-transplant/ post-recruitment in those who remained on the waiting list for a kidney transplant, or those who received a DD or LD kidney transplant after recruitment.

338x190mm (96 x 96 DPI)

Supplementary Data

Supplementary Data 1. Interview schedule for semi-structured interviews with LD and DD recipients.

The interview schedule included prompt questions, but each schedule was tailored to the individual and their questionnaire responses to the Renal Dependent Quality of Life (RDQoL) Questionnaire and the Renal Treatment Satisfaction Questionnaires (RTSQs).

HISTORY
Can you describe the process that you went through before you received a kidney transplant?

<u>All patients:</u>
- When did you first start having problems with your kidneys?
- How did your kidney problems develop?
- Were you placed on the transplant list? When?

For patients who received a DD transplant:
- How long were you waiting for a kidney?
- How did you feel about the time you spent waiting?
- Was the option of a living donor mentioned to you?

For patients who received an LD transplant:
- Who first suggested a living donor to you?
- How did you find information about living donation?
    - How satisfied were you with the information you were given?
- How did you bring the topic up with your family or friends?
    - Did you feel able to talk about it?
- Who donated a kidney to you?
    - How are they doing now?

QUALITY OF LIFE
Ok, so now I'd like to move on and talk to you about your quality of life.
In the questionnaires that you completed recently, you report that overall, your quality of life is excellent / very good / good / neither good nor bad / bad / very bad / extremely bad.

You said that your renal condition does / does not have an impact on your quality of life.
Can you give me an example of how (aspect of life) has been affected?
Other aspect(s) of your life that appeared to be most impacted by your renal condition was/were …………
Can you tell me in what ways your renal condition impacts on ………………….?

For patients with QoL changes over time:
In the questionnaires, you report that your quality of life has changed since you received a kidney transplant. Can you tell me why that is?

TREATMENT SATISFACTION
In terms of your treatment satisfaction, I can see from your questionnaires that you are very satisfied / satisfied / a little dissatisfied / dissatisfied / very dissatisfied with your current treatment. Can you tell me why?
I can see from your questionnaires that the aspect(s) of your treatment that you are most satisfied with was/were ………… Can you tell me why?
You were least satisfied with ………….. Can you tell me why?

For peer review only

Your treatment satisfaction did / did not change over time from 3 months after recruitment / transplant to 12 months after recruitment / transplant. Can you tell me why?

How might your satisfaction with treatment be improved?

For peer review only

Supplementary Data 2. Table 1. Summary of outcome measures

		No. of items	Range
Quality of life (ADDQoL and RDQoL)			
Generic QoL item	A single item assessing present QoL.	1	+3 (excellent) to -3 (extremely bad)
Renal Dependent Quality of Life (RDQoL)	Weighted-impact scores calculated by multiplying (a) impact of renal condition on aspect of life with (b) importance rating of aspect of life and summing all applicable items to give an average weighted-impact (AWI) score reflecting the impact of the renal condition on QoL.	21 (6 N/A options) *	-9 (most negative weighted impact) to +3 (most positive weighted impact)
Well-being (W-BQ12)			
Total well-being	12 items scored on 4-point scales from 3 (all the time) to 0 (not at all). Overall well-being score calculated by combining the scores from the 3 subscales (negative well-being, energy, positive well-being). Higher scores indicate greater well-being during the past few weeks.	12	36-0
Health status (EQ-5D-5L)			
Utility values	5 dimensions of health (today) rated on 5 levels converted to population preference values using the new value set for England.[18] The higher the score, the greater the preference utility.	5	+1.00 (perfect health) to -0.281 (worse than death)
EQ-VAS ratings	Self-reported health (today) on visual analogue scale from 100 (best health you can imagine) to 0 (worst health you can imagine).	1	100-0
Treatment satisfaction (RTSQs/c)			
Renal Treatment Satisfaction Questionnaire (RTSQ) status version (RTSQs)	Items assess satisfaction with current renal treatment on 7-point scales from 6 (e.g. very satisfied) to 0 (e.g. very dissatisfied). Items are summed to give a total satisfaction score. The higher the score, the greater the satisfaction with renal treatment.	13	78-0
RTSQ change version (RTSQc)	Items assess satisfaction with current renal treatment compared with satisfaction with previous renal treatment, from +3 (e.g. much more satisfied now) to -3 (e.g. much less satisfied now). A score of 0 indicates no change in satisfaction. Items are summed to give a total change in satisfaction score.	13	+39 (much more satisfied now) to 39 (much less satisfied now)

*after preliminary psychometric analyses, only 17 of the items (including 3 with not applicable options) were included in the Average Weighted Impact (AWI) score (see Appendix 2). The range of AWI scores remains the same.

For peer review only

Supplementary Data 3. Psychometric properties of the RDQoL and RTSQs/c

The Renal Dependent Quality of Life (RDQoL) questionnaire was designed to examine the impact of renal conditions on QoL.[1] Following the template of the Audit of Diabetes-Dependent QoL (ADDQoL),[2,3] the RDQoL contains two overview items and a series of domain-specific items covering different aspects of life commonly impacted by a renal condition and its treatment. Each domain-specific item is worded *'If did not have a renal condition my…would be'*, followed by a rating scale for the impact of renal condition on that domain of life from -3 (e.g. very much better) to +1 (e.g. worse), and a rating of the importance of the domain for QoL from very important (+3) to not at all important (0). The impact and importance scores are multiplied to give a weighted impact score for each item, ranging from -9 (most negative weighted impact on QoL) to +3 (most positive weighted impact). Some of the items include preliminary questions to determine if the domain is applicable to the individual (e.g. work). Each of the applicable weighted impact scores are summed and the total is divided by the number of applicable items to give an average weighted impact (AWI) score, also ranging from -9 to +3.

The Renal Treatment Satisfaction Questionnaire (RTSQ) is a measure of satisfaction with renal treatment. Following the templates of the Diabetes Treatment Satisfaction Questionnaire (DTSQ)[4-6] there are two versions: the status version (RTSQs)[7] and the change version (RTSQc). The RTSQs assesses level of satisfaction with current renal treatment. The 13 items are summed to give an overall treatment satisfaction score, with higher scores indicating greater satisfaction (range=78-0). The RTSQc was developed to overcome the ceiling effects sometimes found with the status version. Using similar item stems as the RTSQs but different response options, the RTSQc asks participants to compare satisfaction with their current treatment with satisfaction with their previous treatment (range +39 e.g. much more satisfied to -39 much less satisfied). Barendse et al[7] conducted analyses on the original 11-item RTSQs, and found that it had acceptable reliability. Although a forced one-factor structure was acceptable for all patients, a two-factor structure was found for transplant recipients, and a three-factor structure for non-transplant patients.[6] The 13-item version of the RTSQs has additional

For peer review only

items related to satisfaction with the side effects of treatment, and satisfaction with the demands of treatment. No psychometric analyses have been published using the 13-item version, and none have been conducted with the RTSQc. Analyses, therefore, were conducted to test the psychometric properties of these measures in this sample.

In ATTOM, the number of participants in each group recruited were as follows; 104 DD recipients, 94 LD recipients, 98 patients wait-listed for a transplant, 136 participants receiving PD or HD, and 16 patients whose transplant failed during ATTOM. Participants were invited to complete the measures at 12m post-transplant or post-recruitment. There was a possibility that differences between the groups might create artefactual correlations in the combined sample, if the subgroup mean scores differed systematically across items. The possibility of artefactual correlations was therefore investigated by converting the scores on each measure to standardised z scores within each subgroup before combining the sub-sets of scores and conducting the factor analyses. Such standardisation renders group means identical and thereby removes the possibility of correlations caused by subgroup differences. Forced one-factor solutions on the raw scores, and then on the Z scores, produced two sets of factor loadings that were then compared using correlation and regression cross-check analyses.

The crosscheck analyses tested whether the transplant groups (DD and LD recipients) and non-transplant groups could be combined for these analyses. The non-transplant groups included dialysis and pre-dialysis patients including those wait-listed for a kidney transplant, and those whose transplants failed during ATTOM. For ease of analysis, these were grouped into those on HD, PD, or pre-dialysis. Participants with missing data on any items were excluded from the analyses. The transplant groups could be combined for analyses for the RDQoL, RTSQs and RTSQc. The non-transplant groups (on either HD, PD or pre-dialysis) could be combined for the RDQoL and RTSQs, whilst only the HD and PD groups could be combined for the RTSQc. Those not yet on dialysis formed a much smaller sub-group (*n*=15), so were not included in separate analyses.

For peer review only

RDQoL

Factor structure

Analyses were conducted to confirm the factor structure of the RDQoL, RTSQs, and RTSQc. Parallel analyses were conducted to identify how many factors are acceptable to retain in the structure of each measure. Principal Axis Factoring (PAF) was then used to examine the variance and the item loadings unto these factors. A Kaiser-Meyer-Olkin Measure of Sampling Adequacy (KMO) value of 0.60 or above, and correlation coefficients of 0.30 and above between each item indicates that the analysis is appropriate and the items should be retained. An item loading above 0·40 is considered acceptable. See Tables 5 and 6 for a summary of the descriptive statistics, reliability analysis, and factor structure of the RDQoL for transplant and non-transplant groups. Parallel analysis indicated that a one-factor solution was acceptable for both the transplant and non-transplant groups.

Transplant (LD and DD) group: Principal Axis Factoring (PAF) with a forced one-factor solution led to 48·04% of the variance being explained, with item loadings from 0.266 to 0.855 (KMO=0.92, $\chi^2_{(210)}$=1738.60, p<0.001). The spiritual/religious life item had the lowest item loading of 0.266, and correlated with only one other item above 0.30, so was removed, and the analysis re-run. This led to 49·97% of the variance being explained, with more acceptable loadings ranging from 0.417 to 0.856 (KMO=0.93, $\chi^2_{(210)}$=1715.451, p<0.001). Item 19 (fuss/worry), although displaying an acceptable factor loading (0.417), correlated with few other items above 0.30.

Non-transplant group: Principal Axis Factoring with a forced one-factor solution led to 38.95% of the variance being explained, with item loadings from 0.250 to 0.730 (KMO=0.92, $\chi^2_{(210)}$=1431.71, p<0.001). Item 2 (work, 0.250), item 4 (holidays 0.331), and item 14 (religious/spiritual life 0.349) had item loadings below 0.40. Work was only correlated above 0.30 with one other item, whilst holidays and spiritual/religious life were each correlated with four other items above 0.30. These items were removed, and the analysis re-run. This led to 43.70% of the variance being explained, with item loadings from 0.552 to 0.740 (KMO=0.93, $\chi^2_{(153)}$=1308.43, p<0.001).

For peer review only

Supplementary Table 2. Descriptive statistics, reliability analysis, and forced one-factor solution Principal Axis Factoring (PAF) on the RDQoL weighted impact scores in the transplant recipients (with deceased and living donor kidneys; *n* = 137).

	Missing N	Not applicable	Mean	Median	*SD*	Corrected Item-total correlation	Cronbach's alpha if item deleted*	PAF Item Loadings
**1. Leisure activities**	1	-	-2.72	-2.00	2.68	0.717	0.940	0.741
**2. Work**	3	37	-2.46	0.00	3.18	0.610	0.942	0.617
**3. Local/long distance journeys**	1	-	-1.79	-1.00	2.41	0.612	0.942	0.630
**4. Holidays**	3	9	-2.83	-2.00	2.96	0.716	0.940	0.735
**5. Physical ability**	2	-	-3.06	-3.00	2.80	0.783	0.939	0.811
**6. Family**	1	1	-2.42	-1.50	2.89	0.714	0.940	0.743
**7. Friendships/social life**	0	-	-2.34	-1.50	2.88	0.789	0.939	0.815
**8. Close personal relationship**	3	14	-2.37	0.00	3.10	0.713	0.940	0.746
**9. Sex life**	2	16	-2.61	-2.00	2.99	0.723	0.940	0.751
**10. Physical appearance**	3	-	-2.37	-2.00	2.60	0.540	0.943	0.561
**11. Self-confidence**	1	-	-2.25	-2.00	2.74	0.783	0.939	0.813
**12. Motivation**	4	-	-2.26	-2.00	2.86	0.825	0.938	0.855
**13. People react to me**	1	-	-0.84	0.00	1.78	0.607	0.942	0.633
**14. Spiritual/religious life**	0	75	-0.31	0.00	1.75	-	-	0.266
**15. Feelings about the future**	0	-	-3.42	-2.00	3.34	0.576	0.943	0.588
**16. Financial situation**	1	-	-2.61	-2.00	3.23	0.686	0.941	0.696
**17. Living conditions**	1	-	-1.41	0.00	2.40	0.659	0.941	0.685
**18. Dependence on others**	2	-	-2.40	-1.00	3.06	0.543	0.943	0.548
**19. People fuss/worry**	0	-	-3.20	-2.00	3.05	0.417	0.945	0.427
**20. Freedom to eat**	0	-	-1.76	-1.00	2.45	0.614	0.942	0.635
**21. Freedom to drink**	0	-	-1.69	-1.00	2.51	0.603	0.942	0.614

*Total Cronbach's alpha = 0·944.

Reliability analyses

Cronbach's alpha (α) was calculated as a test of internal consistency; how closely related the items in the measure are as a group. Scores range from 0 to 1, with a score above 0.70 commonly being used as a threshold for acceptable reliability for group comparison with 0.80 providing a more stringent threshold. Analyses are repeated, removing one item at a time to calculate how many missing items a measure can tolerate, whilst still retaining an acceptable alpha coefficient. Even if the alpha

For peer review only

coefficient is above the threshold, no more than 50% of the items in a scale or subscale are allowed to be missing, because to do so is likely to produce too much distortion of the construct measured by the scale.

Transplant (LD and DD) group: Analyses conducted using all items except item 14 (spiritual/religious life) revealed that the RDQoL has excellent reliability (Cronbach's α=0.944). The removal of item 19 (fuss or worry) would improve the alpha coefficient from 0.944 to 0.945. The fuss and worry item also had the lowest item-total correlations (0·417), and the lowest item loadings (0.427), suggesting that its removal could improve the factor structure and reliability of the RDQoL.

Non-transplant group: Reliability analyses revealed that, not including item 2 (work) and 14 (spiritual/religious life), the RDQoL has excellent reliability (Cronbach's α=0.920; Table 6). The removal of item 4 (holidays) would improve the alpha coefficient from 0.920 to 0.922. The item had a low factor loading (0.331) and low item-total correlation (r = 0.326).

Not including those items that have a non-applicable option, reliability analyses indicated that the questionnaire (14 items) could tolerate up to seven missing items and still retain a Cronbach's α coefficient above 0.70 for the transplant group (α = 0.786) and non-transplant group (α = 0.792).

For peer review only

Supplementary Table 3. Descriptive statistics, reliability analysis, and forced one-factor solution Principal Axis Factoring (PAF) on the RDQoL weighted impact scores in the non-transplant groups (*n*= 172).

	Missing N	**Not applicable**	**Mean**	**Median**	***SD***	**Corrected Item-total correlation**	**Cronbach's alpha if item deleted***	**PAF Item Loadings**
**1. Leisure activities**	3	-	-4.84	-4.00	2.88	0.587	0.916	0.604
**2. Work**	2	85	-2.85	0.00	3.55	-	-	0.250
**3. Local/long distance journeys**	5	-	-4.34	-4.00	3.27	0.545	0.917	0.556
**4. Holidays**	2	30	-4.94	-6.00	3.42	0.326	0.922	0.331
**5. Physical ability**	1	-	-5.64	-6.00	2.81	0.576	0.916	0.582
**6. Family**	3	1	-5.14	-6.00	3.42	0.619	0.915	0.650
**7. Friendships and social life**	1	-	-4.59	-4.00	3.22	0.676	0.914	0.695
**8. Close personal relationship**	3	21	-4.13	-4.00	3.57	0.657	0.914	0.698
**9. Sex life**	1	35	-3.69	-4.00	3.24	0.611	0.915	0.638
**10. Physical appearance**	0	-	-2.92	-2.00	2.99	0.650	0.942	0.688
**11. Self-confidence**	1	-	-3.40	-2.00	3.14	0.659	0.914	0.702
**12. Motivation**	0	-	-4.20	-4.00	3.05	0.696	0.913	0.730
**13. People react to me**	2	-	-1.45	0.00	2.22	0.542	0.917	0.584
**14. Spiritual/religious life**	2	104	-0.74	0.00	2.03	-	-	0.349
**15. Feelings about the future**	1	-	-5.20	-6.00	3.35	0.682	0.913	0.701
**16. Financial situation**	1	-	-3.79	-4.00	3.50	0.490	0.918	0.519
**17. Living conditions**	2	-	-3.09	-2.00	3.26	0.609	0.915	0.665
**18. Dependence on others**	3	-	-3.95	-4.00	3.27	0.660	0.914	0.695
**19. People fuss/worry**	1	-	-3.64	-4.00	3.16	0.564	0.916	0.582
**20. Freedom to eat**	0	-	-4.56	-4.00	3.24	0.557	0.916	0.570
**21. Freedom to drink**	0	-	-4.84	-4.00	3.35	0.531	0.917	0.543

*Total Cronbach's alpha = 0.920.

These findings suggest that item 14 (spiritual/religious life) should be removed for both groups, as the majority of people reported either not having a spiritual or religious life (54.4% transplant group, 60.4% non-transplant group), or reported that it was not impacted by their renal condition (70.8% transplant group, 52.8% non-transplant group). Removing item 19 (fuss or worry) would improve the psychometrics of the questionnaire for transplant recipients. For the non-transplant group, treating item 2 (work) and item 4 (holidays) as separate items to the AWI scores may be indicated. Although the holidays item was relevant for the majority of the patients (93.6% transplant group, 73.8% non-transplant group), the analyses indicate that its removal from the overall AWI scores would improve the factor structure and reliability for the non-transplant group.

For peer review only

Supplementary Table 4. Reliability analysis and forced one-factor solution Principal Axis Factoring (PAF) on 17 items of the RDQoL weighted impact scores for the transplant group (*n*=142) and non-transplant group (*n*=158).

	Transplant group			Non-transplant group		
	**Corrected Item-total correlation**	**Cronbach's alpha if item deleted***	**PAF Item Loadings**	**Corrected Item-total correlation**	**Cronbach's alpha if item deleted***	**PAF Item Loadings**
**1. Leisure activities**	0.708	0.934	0.729	0.575	0.915	0.596
**3. Local/long distance journeys**	0.592	0.937	0.608	0.529	0.916	0.550
**5. Physical ability**	0.784	0.932	0.812	0.547	0.915	0.565
**6. Family**	0.727	0.934	0.753	0.622	0.913	0.658
**7. Friendships/social life**	0.761	0.933	0.791	0.666	0.912	0.696
**8. Close personal relationship**	0.718	0.934	0.747	0.658	0.912	0.695
**9. Sex life**	0.727	0.934	0.751	0.622	0.913	0.650
**10. Physical appearance**	0.557	0.937	0.577	0.645	0.913	0.674
**11. Self-confidence**	0.786	0.932	0.810	0.673	0.912	0.714
**12. Motivation**	0.817	0.932	0.848	0.709	0.911	0.744
**13. People react to me**	0.627	0.937	0.650	0.553	0.916	0.585
**15. Feelings about the future**	0.570	0.938	0.584	0.672	0.912	0.699
**16. Financial situation**	0.686	0.935	0.701	0.497	0.917	0.516
**17. Living conditions**	0.662	0.935	0.689	0.623	0.913	0.651
**18. Dependence on others**	0.496	0.939	0.512	0.637	0.913	0.668
**20. Freedom to eat**	0.611	0.936	0.629	0.553	0.915	0.572
**21. Freedom to drink**	0.587	0.937	0.600	0.530	0.916	0.548

*Transplant group total Cronbach's alpha 0.939; non-transplant group total Cronbach's alpha 0.918

Running the analyses again with the removal of items 2 (work), 4 (holidays), 14 (spiritual/religious life), and 19 (fuss/worry) for both groups indicated an acceptable factor structure and good reliability (Table 7). Therefore, to be able to calculate the AWI scores for all participants, without negatively impacting the psychometrics of the questionnaires, it was decided that item 2 (work) and 4 (holidays) items would be considered as separate items, whilst items 14 (spiritual/religious life) and 19 (fuss/worry) would be removed.

For peer review only

RTSQs

Factor structure: Parallel analysis indicated that a one-factor solution was optimal for both the transplant and non-transplant groups (see Table 8). For the transplant group, Principal Axis Factoring with a forced one-factor solution explained 50.65% of the variance, with item loadings from 0.521 to 0.814 (KMO=0.88, $\chi^2_{(78)}$=1197.74, p<0.001). Similar results were found with the non-transplant group, with 50.80% of the variance being explained, with item loadings from 0.452 to 0.848 (KMO=0.91, $\chi^2_{(78)}$=1321.69, p<0.001).

Reliability analyses: Previous research on the 11-item RTSQs[24] indicated that a forced one-factor solution was acceptable and up to 5 items could be tolerated whilst retaining an alpha coefficient of 0.79. Reliability analyses on the 13-item RTSQs revealed that it has excellent reliability for the transplant (α=0.905) and non-transplant groups (α=0.918). The removal of item 3 (side effects of treatment) would improve the alpha coefficient for both groups, but the item was correlated with the majority of the other items above 0.30, indicating that it should be included. All other items contributed to the reliability of the measure. It was possible to remove up to five items in this 13-item measure and retain a Cronbach's α coefficient above 0.80 for the transplant (α = 0.806) and non-transplant groups (α = 0.832). These findings confirmed that optimal scoring of the RTSQs includes all 13 items.

For peer review only

Supplementary Table 5. Descriptive statistics, reliability analysis, and forced one-factor solution Principal Axis Factoring (PAF) on the RTSQs items, completed by the transplant and non-transplant groups.

	Missing *N*	Mean	Median	*SD*	Corrected Item-total correlation	Cronbach's alpha if item deleted*	PAF Item Loadings
**Transplant group (*N* = 156)**							
**1. Overall satisfaction score**	0	5.58	6.00	0.78	0.707	0.896	0.740
**2. Control of renal condition**	0	5.48	6.00	0.83	0.668	0.897	0.721
**3. Side effects**	1	4.44	5.00	1.51	0.547	0.908	0.558
**4. Demands of treatment**	0	5.23	5.00	0.93	0.732	0.893	0.756
**5. Convenience of treatment**	0	5.23	6.00	1.08	0.730	0.893	0.763
**6. Flexibility of treatment**	0	5.22	6.00	1.16	0.518	0.904	0.559
**7. Freedom on treatment**	0	5.52	6.00	0.81	0.701	0.896	0.745
**8. Understanding of treatment**	0	5.63	6.00	0.69	0.526	0.902	0.569
**9. Time taken by treatment**	0	5.43	6.00	0.93	0.755	0.892	0.814
**10. Discomfort or pain**	1	5.32	6.00	1.15	0.522	0.904	0.521
**11. Treatment fits in with lifestyle**	0	5.41	6.00	1.02	0.700	0.894	0.745
**12. Recommend treatment to others**	1	5.75	6.00	0.70	0.607	0.900	0.634
**13. Satisfaction to continue treatment**	1	5.71	6.00	0.70	0.647	0.898	0.684
**Non-transplant group (*N* = 190)**							
**1. Overall satisfaction score**	1	4.99	5.00	1.09	0.522	0.917	0.546
**2. Control of renal condition**	0	4.94	5.00	1.03	0.552	0.916	0.576
**3. Side effects**	0	4.04	4.00	1.51	0.606	0.914	0.635
**4. Demands of treatment**	1	3.97	4.00	1.62	0.786	0.906	0.821
**5. Convenience of treatment**	0	4.19	5.00	1.64	0.812	0.905	0.848
**6. Flexibility of treatment**	0	4.30	5.00	1.71	0.730	0.909	0.764
**7. Freedom on treatment**	0	3.78	4.00	1.78	0.732	0.909	0.764
**8. Understanding of treatment**	0	5.16	5.00	1.05	0.430	0.919	0.452
**9. Time taken by treatment**	0	4.03	4.00	1.77	0.763	0.907	0.795
**10. Discomfort or pain**	1	4.39	5.00	1.46	0.557	0.915	0.579
**11. Treatment fits in with lifestyle**	1	3.77	4.00	1.65	0.699	0.910	0.726
**12. Recommend treatment to others**	3	5.05	6.00	1.31	0.619	0.913	0.642
**13. Satisfaction to continue treatment**	1	4.68	5.00	1.62	0.619	0.913	0.646

*Transplant group total Cronbach's alpha = 0.905. Non-transplant total Cronbach's alpha =0.918

For peer review only

RTSQc

Factor structure: See Table 9 for summaries of descriptive statistics, reliability analyses, and the factor structure of the RTSQc. Parallel analysis indicated that a one-factor solution was acceptable for the transplant and non-transplant groups. Principal Axis Factoring with a forced one-factor solution explained 56.22% of the variance with item loadings from 0.466 to 0.832 (KMO=0.90, $\chi^2_{(78)}$ =1353.40, p<0.001). Similar results were found for the non-transplant groups; 67.87% of the variance was explained, with item loadings from 0.601 to 0.935 (KMO=0.93, $\chi^2_{(78)}$ =1929.77, p<0.001).

Reliability analyses: Analyses revealed that the RTSQc has excellent reliability for the transplant (α=0.930) and non-transplant group (α=0.960). The removal of item 8 (understanding of treatment) would improve the alpha coefficient slightly for both groups. Despite this, item was 8 correlated with other items above 0.30, and the item loadings were acceptable, indicating that it should be included. Reliability analyses indicated that in this 13-item measure, it was possible to remove up to six items and retain a Cronbach's α coefficient of 0.826 for the transplant group and 0.906 for the non-transplant group.

In line with previous research, these findings suggest that the RTSQs and RTSQc can be considered to have a one-factor structure across various treatment groups and time points. Although the exclusion of various items is possible for the RTSQs and RTSQc, all items contribute to the factor structure, and the reliability is excellent using all 13 items.

For peer review only

Supplementary Table 6. Descriptive statistics, reliability analysis, and forced one-factor solution Principal Axis Factoring (PAF) on the RTSQc items, completed by transplant (DD and LD) and non-transplant (HD and PD) groups.

	Missing *N*	Mean	Median	*SD*	Corrected Item-total correlation	Cronbach's alpha if item deleted*	PAF Item Loadings for 1 factor solution
**LD and DD groups (*n*=150)**							
**1. Overall satisfaction score**	3	2.42	3.00	1.07	0.702	0.924	0.723
**2. Control of renal condition**	3	2.50	3.00	1.03	0.720	0.923	0.748
**3. Side effects**	3	1.69	2.00	1.54	0.599	0.930	0.616
**4. Demands of treatment**	4	2.32	3.00	1.08	0.800	0.920	0.833
**5. Convenience of treatment**	4	2.27	3.00	1.26	0.783	0.920	0.824
**6. Flexibility of treatment**	4	2.26	3.00	1.21	0.678	0.924	0.714
**7. Freedom on treatment**	4	2.33	3.00	1.09	0.796	0.921	0.832
**8. Understanding of treatment**	5	2.17	3.00	1.15	0.449	0.932	0.486
**9. Time taken by treatment**	5	2.37	3.00	1.07	0.739	0.922	0.764
**10. Discomfort or pain**	8	2.18	3.00	1.24	0.616	0.927	0.628
**11. Treatment fits in with lifestyle**	7	2.32	3.00	1.16	0.760	0.922	0.803
**12. Recommend treatment to others**	6	2.58	3.00	0.99	0.564	0.928	0.581
**13. Satisfaction to continue treatment**	7	2.50	3.00	1.08	0.787	0.921	0.814
**HD and PD groups (*n*=170)**							
**1. Overall satisfaction score**	0	1.30	2.00	1.73	0.788	0.957	0.802
**2. Control of renal condition**	0	1.48	2.00	1.60	0.676	0.960	0.686
**3. Side effects**	0	0.89	1.00	1.77	0.777	0.957	0.791
**4. Demands of treatment**	0	0.96	1.00	1.72	0.888	0.955	0.910
**5. Convenience of treatment**	0	0.98	1.00	1.82	0.912	0.954	0.935
**6. Flexibility of treatment**	0	0.91	1.00	1.68	0.832	0.956	0.852
**7. Freedom on treatment**	0	0.62	1.00	1.86	0.941	0.956	0.862
**8. Understanding of treatment**	0	1.76	2.00	1.27	0.592	0.961	0.601
**9. Time taken by treatment**	0	0.89	2.00	1.80	0.862	0.955	0.884
**10. Discomfort or pain**	0	0.91	2.00	1.57	0.733	0.958	0.746
**11. Treatment fits in with lifestyle**	0	0.73	2.00	1.73	0.851	0.955	0.871
**12. Recommend treatment to others**	0	1.69	3.00	1.59	0.737	0.958	0.748
**13. Satisfaction to continue treatment**	0	1.39	1.00	1.73	0.748	0.958	0.761

*LD and DD groups total Cronbach's alpha = 0.930. HD and PD groups total Cronbach's alpha = 0.960. LD=living donor; DD= deceased donor; HD=haemodialysis' PD= peritoneal dialysis.

For peer review only

References

1. Bradley C. Design of a Renal-Dependent Individualized Quality of Life Questionnaire. *Adv Perit Dial* 1997; 13: 116-120.
2. Bradley C, Todd C, Gorton T, et al. The development of an individualised measure of perceived impact of diabetes on quality of life: The ADDQoL. *Qual Life Res* 1999; 8(1-2): 79-91.doi:10.1023/A:1026485130100.
3. Wee HL, Tan CE, Goh SY, Li SC. Usefulness of the Audit of Diabetes-Dependent Quality-of-Life (ADDQoL) Questionnaire in patients with diabetes in a multi-ethnic Asian country. *Pharmacoeconomics* 2006; 24(7): 673-682.
4. Bradley C, Lewis KS. Measures of psychological well-being and treatment satisfaction developed from the responses of people with tablet-treated diabetes. Diabetic Med 1990; 7, 445-451.
5. Bradley C. The Diabetes Treatment Satisfaction Questionnaire: DTSQ. In Bradley C. (Ed). Handbook of Psychology and Diabetes: A guide to psychological measurement in diabetes research and practice. Chur Switzerland: Harwood Academic Publishers, 1994; 111-132.
6. Howorka K, Pumprla J, Schlusche C, et al. Dealing with ceiling baseline treatment satisfaction level in patients with diabetes under flexible, functional insulin treatment: Assessment of improvements in treatment satisfaction with a new insulin analogue. *Qual Life Res* 2000; 9: 915-930.
7. Barendse SM, Speight J, Bradley C. The Renal Treatment Satisfaction Questionnaire (RTSQ): A measure of satisfaction with treatment for chronic kidney failure. *Am J Kidney Dis* 2005; 45: 572-579.

For peer review only

Your article may not currently address all the items on the checklist. Please modify your text to include the missing information. If you are certain that an item does not apply, please write "n/a" and provide a short explanation.

Upload your completed checklist as an extra file when you submit to a journal.

In your methods section, say that you used the SRQRreporting guidelines, and cite them as:

O'Brien BC, Harris IB, Beckman TJ, Reed DA, Cook DA. Standards for reporting qualitative research: a synthesis of recommendations. Acad Med. 2014;89(9):1245-1251.

		Reporting Item	Page Number
**Title**			
	#1	Concise description of the nature and topic of the study identifying the study as qualitative or indicating the approach (e.g. ethnography, grounded theory) or data collection methods (e.g. interview, focus group) is recommended	1
**Abstract**			
	#2	Summary of the key elements of the study using the abstract format of the intended publication; typically includes background, purpose, methods, results and conclusions	3 (line 2) - 4 (line 2)
**Introduction**			
Problem formulation	#3	Description and signifcance of the problem / phenomenon studied: review of relevant theory and empirical work; problem statement	5 (line 1) – 7 (line 17)
Purpose or research question	#4	Purpose of the study and specific objectives or questions	7 (lines 18) – 8 (line 3)
**Methods**			
Qualitative approach and research paradigm	#5	Qualitative approach (e.g. ethnography, grounded theory, case study, phenomenolgy, narrative research) and guiding theory if appropriate; identifying the research paradigm	7 (lines 15-17); 13 (lines 21-23).

For peer review only

		(e.g. postpositivist, constructivist / interpretivist) is also recommended; rationale. The rationale should briefly discuss the justification for choosing that theory, approach, method or technique rather than other options available; the assumptions and limitations implicit in those choices and how those choices influence study conclusions and transferability. As appropriate the rationale for several items might be discussed together.	
Researcher characteristics and reflexivity	#6	Researchers' characteristics that may influence the research, including personal attributes, qualifications / experience, relationship with participants, assumptions and / or presuppositions; potential or actual interaction between researchers' characteristics and the research questions, approach, methods, results and / or transferability	10 (lines 16-18)
Context	#7	Setting / site and salient contextual factors; rationale	7 (lines 15-17); 8 (line 6) – 10 (line 12).
Sampling strategy	#8	How and why research participants, documents, or events were selected; criteria for deciding when no further sampling was necessary (e.g. sampling saturation); rationale	10 (line 3-13); 13 (lines 18-25)
Ethical issues pertaining to human subjects	#9	Documentation of approval by an appropriate ethics review board and participant consent, or explanation for lack thereof; other confidentiality and data security issues	8 (lines 15-16); 41 (line 1)
Data collection methods	#10	Types of data collected; details of data collection procedures including (as appropriate) start and stop dates of data collection and analysis, iterative process, triangulation of sources / methods, and modification of	10 (lines 1-19)' 13 (lines 18-25)

For peer review only

		procedures in response to evolving study findings; rationale	
Data collection instruments and technologies	#11	Description of instruments (e.g. interview guides, questionnaires) and devices (e.g. audio recorders) used for data collection; if / how the instruments(s) changed over the course of the study	10 (lines 14-16); Supplemental data file 1.
Units of study	#12	Number and relevant characteristics of participants, documents, or events included in the study; level of participation (could be reported in results)	14 (line 24)- 15 (line 4)
Data processing	#13	Methods for processing data prior to and during analysis, including transcription, data entry, data management and security, verification of data integrity, data coding, and anonymisation / deidentification of excerpts	13 (lines 18-25)
Data analysis	#14	Process by which inferences, themes, etc. were identified and developed, including the researchers involved in data analysis; usually references a specific paradigm or approach; rationale	13 (lines 18-25); 17 (lines 8-11)
Techniques to enhance trustworthiness	#15	Techniques to enhance trustworthiness and credibility of data analysis (e.g. member checking, audit trail, triangulation); rationale	13 (lines 18-25)
**Results/findings**			
Syntheses and interpretation	#16	Main findings (e.g. interpretations, inferences, and themes); might include development of a theory or model, or integration with prior research or theory	17 (line 8) – 18 (line 5)
Links to empirical data	#17	Evidence (e.g. quotes, field notes, text excerpts, photographs) to substantiate analytic findings	Table 5
**Discussion**			

For peer review only

Intergration with prior work, implications, transferability and contribution(s) to the field	#18	Short summary of main findings; explanation of how findings and conclusions connect to, support, elaborate on, or challenge conclusions of earlier scholarship; discussion of scope of application / generalizability; identification of unique contributions(s) to scholarship in a discipline or field	18 (line 23) – 19 (line 14)
Limitations	#19	Trustworthiness and limitations of findings	20 (lines 12-19)
**Other**			
Conflicts of interest	#20	Potential sources of influence of perceived influence on study conduct and conclusions; how these were managed	40 (lines 4-12)
Funding	#21	Sources of funding and other support; role of funders in data collection, interpretation and reporting	39 (line 24) – 40 (line 2)

None The SRQR checklist is distributed with permission of Wolters Kluwer © 2014 by the Association of American Medical Colleges. This checklist can be completed online using https://www.goodreports.org/, a tool made by the EQUATOR Network

For peer review only

STROBE 2007 (v4) checklist of items to be included in reports of observational studies in epidemiology*
Checklist for cohort, case-control, and cross-sectional studies (combined)

Section/Topic	Item #	Recommendation	Reported on page #
**Title and abstract**	1	(*a*) Indicate the study's design with a commonly used term in the title or the abstract	1 (line 1-4)
		(*b*) Provide in the abstract an informative and balanced summary of what was done and what was found	3 (line 2) - 4 (line 2)
**Introduction**			
Background/rationale	2	Explain the scientific background and rationale for the investigation being reported	5 (line 1) – 7 (line 17)
Objectives	3	State specific objectives, including any pre-specified hypotheses	7 (line 18) – 8 (line 3)
**Methods**			
Study design	4	Present key elements of study design early in the paper	8 (line 6 -15)
Setting	5	Describe the setting, locations, and relevant dates, including periods of recruitment, exposure, follow-up, and data collection	8 (line 6) – 10 (line 2)
Participants	6	(*a*) *Cohort study*—Give the eligibility criteria, and the sources and methods of selection of participants. Describe methods of follow-up<br>*Case-control study*—Give the eligibility criteria, and the sources and methods of case ascertainment and control selection. Give the rationale for the choice of cases and controls<br>*Cross-sectional study*—Give the eligibility criteria, and the sources and methods of selection of participants	8 (line 11) – 10 (line 2)
		(*b*) *Cohort study*—For matched studies, give matching criteria and number of exposed and unexposed<br>*Case-control study*—For matched studies, give matching criteria and the number of controls per case	8 (line 11) – 10 (line 2)
Variables	7	Clearly define all outcomes, exposures, predictors, potential confounders, and effect modifiers. Give diagnostic criteria, if applicable	10 (line 6) – 12 (line 18)
Data sources/ measurement	8*	For each variable of interest, give sources of data and details of methods of assessment (measurement). Describe comparability of assessment methods if there is more than one group	8 (line 20) – 9 (line 15)
Bias	9	Describe any efforts to address potential sources of bias	12 (line 18) - 13 (line 3)
Study size	10	Explain how the study size was arrived at	9 (lines 15-20)
Quantitative variables	11	Explain how quantitative variables were handled in the analyses. If applicable, describe which groupings were chosen and why	12 (line 18) – 13 (line 18)
Statistical methods	12	(*a*) Describe all statistical methods, including those used to control for confounding	12 (line 18) – 13 (line 18); 15 (lines 6-26).
		(*b*) Describe any methods used to examine subgroups and interactions	12 (line 26) – 13 (line 1)

For peer review only

		(*c*) Explain how missing data were addressed	14 (lines 23-24)
		(*d*) *Cohort study*—If applicable, explain how loss to follow-up was addressed<br>*Case-control study*—If applicable, explain how matching of cases and controls was addressed<br>*Cross-sectional study*—If applicable, describe analytical methods taking account of sampling strategy	9 (lines 15-18)
		(*e*) Describe any sensitivity analyses	n/a
**Results**			
Participants	13*	(a) Report numbers of individuals at each stage of study—eg numbers potentially eligible, examined for eligibility, confirmed eligible, included in the study, completing follow-up, and analysed	Figure 1.
		(b) Give reasons for non-participation at each stage	Figure 1.
		(c) Consider use of a flow diagram	Figure 1.
Descriptive data	14*	(a) Give characteristics of study participants (eg demographic, clinical, social) and information on exposures and potential confounders	Table 1. 14 (lines 1-24)
		(b) Indicate number of participants with missing data for each variable of interest	14 (lines 23-24)
		(c) *Cohort study*—Summarise follow-up time (eg, average and total amount)	Table 1. 14 (lines 1-24)
Outcome data	15*	*Cohort study*—Report numbers of outcome events or summary measures over time	15 (line 6) – 17 (line 6), Tables 2-4
		Case-control study—Report numbers in each exposure category, or summary measures of exposure	15 (line 6) – 17 (line 6), Tables 2-4
		Cross-sectional study—Report numbers of outcome events or summary measures	15 (line 6) – 17 (line 6), Tables 2-4
Main results	16	(*a*) Give unadjusted estimates and, if applicable, confounder-adjusted estimates and their precision (eg, 95% confidence interval). Make clear which confounders were adjusted for and why they were included	15 (line 6) – 17 (line 6), Tables 2-4
		(*b*) Report category boundaries when continuous variables were categorized	n/a
		(*c*) If relevant, consider translating estimates of relative risk into absolute risk for a meaningful time period	n/a
Other analyses	17	Report other analyses done—eg analyses of subgroups and interactions, and sensitivity analyses	n/a
**Discussion**			
Key results	18	Summarise key results with reference to study objectives	18 (lines 7-22); 19 (line 15) – 20 (line 2)
Limitations	19	Discuss limitations of the study, taking into account sources of potential bias or imprecision. Discuss both direction and magnitude of any potential bias	19 (line 3) - 20 (line 3)
Interpretation	20	Give a cautious overall interpretation of results considering objectives, limitations, multiplicity of analyses, results from similar studies, and other relevant evidence	21 (line 13) – 22 (line

For peer review only

			3)
Generalisability	21	Discuss the generalisability (external validity) of the study results	21 (line 13) – 22 (line 3)
**Other information**			
Funding	22	Give the source of funding and the role of the funders for the present study and, if applicable, for the original study on which the present article is based	39 (line 24) – 40 (line 2)

*Give information separately for cases and controls in case-control studies and, if applicable, for exposed and unexposed groups in cohort and cross-sectional studies.
Note: An Explanation and Elaboration article discusses each checklist item and gives methodological background and published examples of transparent reporting. The STROBE checklist is best used in conjunction with this article (freely available on the Web sites of PLoS Medicine at http://www.plosmedicine.org/, Annals of Internal Medicine at http://www.annals.org/, and Epidemiology at http://www.epidem.com/). Information on the STROBE Initiative is available at www.strobe-statement.org.

For peer review only

BMJ Open

Changes in quality of life (QoL) and other patient-reported outcome measures (PROMs) in living-donor and deceased-donor kidney transplant recipients and those awaiting transplantation in the UK ATTOM programme: a longitudinal cohort questionnaire survey with additional qualitative interviews.

Journal:	*BMJ Open*
Manuscript ID	bmjopen-2020-047263.R1
Article Type:	Original research
Date Submitted by the Author:	13-Jan-2021
Complete List of Authors:	Gibbons, Andrea; University of Winchester Department of Psychology; Royal Holloway University of London, Health Psychology Research Unit<br>Bayfield, Janet; Royal Holloway University of London, Health Psychology Research Unit; Health Psychology Research Ltd<br>Cinnirella, Marco; Royal Holloway University of London, Psychology<br>Draper, Heather; University of Warwick Warwick Medical School, Centre for Biomedical Ethics<br>Johnson, Rachel; NHS Blood and Transplant Organ Donation and Transplantation Directorate<br>Oniscu, Gabriel; Royal Infirmary of Edinburgh, Edinburgh Transplant Centre<br>Ravanan, Rommel; Southmead Hospital, Richard Bright Renal Unit<br>Tomson, Charles; Newcastle Upon Tyne Hospitals NHS Foundation Trust<br>Roderick, Paul; University of Southampton Faculty of Medicine, Academic Unit of Primary Care and Population Sciences, Faculty of Medicine<br>Metcalfe, Wendy; Royal Infirmary of Edinburgh, Transplant Unit<br>Forsythe, John L. R.; Royal Infirmary of Edinburgh, Edinburgh Transplant Centre; NHS Blood and Transplant Organ Donation and Transplantation Directorate, Organ Donation and Transplantation<br>Dudley, Christopher; Southmead Hospital, Richard Bright Renal Unit<br>Watson, Christopher; University of Cambridge, NIHR Cambridge Biomedical Research Centre and the NIHR Blood and Transplant Research Unit in Organ Donation and Transplantation ; Addenbrooke's Hospital<br>Bradley, J Andrew; University of Cambridge, NIHR Cambridge Biomedical Research Centre and the NIHR Blood and Transplant Research Unit in Organ Donation and Transplantation ; Addenbrooke's Hospital<br>Bradley, Clare; Health Psychology Research Ltd; Royal Holloway University of London, Health Psychology Research Unit
**Primary Subject Heading**:	Renal medicine
Secondary Subject Heading:	Patient-centred medicine
Keywords:	Renal transplantation < NEPHROLOGY, TRANSPLANT SURGERY,

	NEPHROLOGY, Dialysis < NEPHROLOGY, Chronic renal failure < NEPHROLOGY, TRANSPLANT MEDICINE

SCHOLARONE™
Manuscripts

I, the Submitting Author has the right to grant and does grant on behalf of all authors of the Work (as defined in the below author licence), an exclusive licence and/or a non-exclusive licence for contributions from authors who are: i) UK Crown employees; ii) where BMJ has agreed a CC-BY licence shall apply, and/or iii) in accordance with the terms applicable for US Federal Government officers or employees acting as part of their official duties; on a worldwide, perpetual, irrevocable, royalty-free basis to BMJ Publishing Group Ltd ("BMJ") its licensees and where the relevant Journal is co-owned by BMJ to the co-owners of the Journal, to publish the Work in this journal and any other BMJ products and to exploit all rights, as set out in our licence.

The Submitting Author accepts and understands that any supply made under these terms is made by BMJ to the Submitting Author unless you are acting as an employee on behalf of your employer or a postgraduate student of an affiliated institution which is paying any applicable article publishing charge ("APC") for Open Access articles. Where the Submitting Author wishes to make the Work available on an Open Access basis (and intends to pay the relevant APC), the terms of reuse of such Open Access shall be governed by a Creative Commons licence – details of these licences and which Creative Commons licence will apply to this Work are set out in our licence referred to above.

Other than as permitted in any relevant BMJ Author's Self Archiving Policies, I confirm this Work has not been accepted for publication elsewhere, is not being considered for publication elsewhere and does not duplicate material already published. I confirm all authors consent to publication of this Work and authorise the granting of this licence.

For peer review only

Changes in quality of life (QoL) and other patient-reported outcome measures (PROMs) in living-donor and deceased-donor kidney transplant recipients and those awaiting transplantation in the UK ATTOM programme: a longitudinal cohort questionnaire survey with additional qualitative interviews.

Andrea Gibbons (0000-0002-7774-0563), Janet Bayfield, Marco Cinnirella, Heather Draper, Rachel J Johnson, Gabriel C Oniscu, Rommel Ravanan, Charles RV Tomson, Paul Roderick, Wendy Metcalfe, John LR Forsythe, Christopher Dudley, Christopher JE Watson, J Andrew Bradley, Clare Bradley.

Department of Psychology, University of Winchester, Winchester, SO22 4NR, UK; the Health Psychology Research Unit, Royal Holloway, University of London, Egham, Surrey, TW20 0EX, UK, Andrea Gibbons, Lecturer in Psychology

Health Psychology Research Unit, Royal Holloway, University of London, Egham, Surrey, TW20 0EX, UK. Janet Bayfield, Administrator/Research Assistant

Department of Psychology, Royal Holloway, University of London, Egham, Surrey, TW20 0EX, UK, Marco Cinnirella, Professor of Applied Social Psychology

Division of Health Sciences, University of Warwick, Warwick Medical School, Coventry, CV4 7AL, UK, Heather Draper, Professor of Biomedical Ethics

NHS Blood and Transplant, Fox Den Road, Stoke Gifford, Bristol, BS34 8RR, UK, Rachel J. Johnson, Assistant Director - Statistics and Clinical Studies

Edinburgh Transplant Centre, Royal Infirmary of Edinburgh, 51 Little France Crescent, Edinburgh, EH16 4SA, UK, Gabriel C. Oniscu, Consultant Transplant Surgeon and Honorary Reader

Richard Bright Renal Unit, Southmead Hospital, Southmead Road, Bristol, BS10 5NB, UK, Rommel Ravanan, Consultant Nephrologist

Department of Renal Medicine, Freeman Hospital, Newcastle upon Tyne, NE12 9NY, UK, Charles Tomson, Consultant Nephrologist

Academic Unit of Primary Care and Population Sciences, Faculty of Medicine, University of Southampton, Tremona Road, Southampton, SO16 6YD, UK, Paul Roderick, Professor of Public Health

Edinburgh Transplant Centre, Royal Infirmary of Edinburgh, 51 Little France Crescent, Edinburgh, EH16 4SA, UK, Wendy Metcalfe, Consultant Nephrologist

Edinburgh Transplant Centre, Royal Infirmary of Edinburgh, 51 Little France Crescent, Edinburgh, EH16 4SA, UK, and Organ Donation and Transplantation, NHS Blood and Transplant, Fox Den Road, Stoke Gifford, Bristol, BS34 8RR, UK, John L. R. Forsythe, Honorary Professor, University of Edinburgh and Medical Director of Organ Donation and Transplantation, NHS Blood and Transplant

Richard Bright Renal Unit, Southmead Hospital, Southmead Road, Bristol, BS10 5NB, UK, Christopher Dudley, Consultant Nephrologist

For peer review only

For peer review only - http://bmjopen.bmj.com/site/about/guidelines.xhtml

Department of Surgery, University of Cambridge, and the NIHR Cambridge Biomedical Research Centre and the NIHR Blood and Transplant Research Unit in Organ Donation and Transplantation at the University of Cambridge, Addenbrooke's Hospital, Hills Road, Cambridge, CB2 0QQ, UK, Christopher J. E. Watson, Professor of Transplantation

Department of Surgery, University of Cambridge and the NIHR Cambridge, Biomedical Research Centre, and the NIHR Blood and Transplant Research Unit in Organ Donation and Transplantation, University of Cambridge, Addenbrooke's Hospital, Hills Road, Cambridge, CB2 0QQ, UK. J. Andrew Bradley, Emeritus Professor

Health Psychology Research (HPR) Ltd, 188 High Street, Egham, Surrey, TW20 9ED; Health Psychology Research Unit, Royal Holloway, University of London, Egham, Surrey, TW20 0EX, UK. Clare Bradley Professor emerita, Royal Holloway, Head of the Health Psychology Research Unit and CEO of HPR Ltd.

Correspondence to: Clare Bradley cb@healthpsychologyresearch.com

For peer review only

Abstract

Objective: To examine quality of life (QoL) and other patient-reported outcome measures (PROMs) in kidney transplant recipients and those awaiting transplantation.

Design: Longitudinal cohort questionnaire surveys and qualitative semi-structured interviews using thematic analysis using a pragmatic approach.

Setting: Completion of generic and disease-specific PROMs at two time points, and telephone interviews with participants UK-wide.

Participants: 101 incident deceased-donor (DD) and 94 incident living-donor (LD) kidney transplant recipients, together with 165 patients on the waiting list (WL) from 18 UK centres recruited to the Access to Transplantation and Transplant Outcome Measures (ATTOM) programme completed PROMs at recruitment (Nov 2011-March 2013) and one-year follow-up. Forty-one of the 165 WL patients received a DD transplant and 26 a LD transplant during the study period, completing PROMs initially as WL patients, and again one-year post-transplant. A subsample of 10 LD and 10 DD recipients participated in qualitative semi-structured interviews.

Results: LD recipients were younger, had more educational qualifications and more often received a transplant before dialysis. Controlling for these and other factors, cross-sectional analyses at 12-months post-transplant suggested better QoL, renal-dependent QoL and treatment satisfaction for LD than DD recipients. WL patients reported worse outcomes compared with both transplant groups. However, longitudinal analyses (controlling for pre-transplant differences) showed that LD and DD recipients reported similarly improved health status and renal- dependent QoL (p<0.01) pre-to post-transplant. WL patients had worsened health status but no change in QoL. Qualitative analyses revealed transplant recipients' expectations influenced their recovery and satisfaction with transplant.

Conclusions: Whilst cross-sectional analyses suggested LD kidney transplantation leads to better QoL and treatment satisfaction, longitudinal assessment showed similar QoL improvements in PROMs for both transplant groups, with better outcomes than for those still wait-listed. Regardless of

For peer review only - http://bmjopen.bmj.com/site/about/guidelines.xhtml

transplant type, clinicians need to be aware that managing expectations is important for facilitating patients' adjustment post-transplant.

Keywords: living donor, deceased donor, kidney transplantation, quality of life, PROMs

Article summary

Strengths and limitations of this study

- We examined a number of patient-reported outcomes in people requiring kidney transplantation, including quality of life, well-being, treatment satisfaction, and health status.
- We collected pre-transplant data for a subsample of deceased-donor and living-donor kidney transplant recipients whilst they were still waiting for a transplant, and assessed outcomes from pre-transplant to one-year post-transplant.
- Controlling for various medical and demographic factors, including age, time on dialysis and education, impacts the findings, removing apparent benefits of living-donor kidneys over deceased-donor kidneys.
- The sample of kidney transplant recipients with pre-transplant data is relatively small (n=67 including 26 with living donors and 41 with deceased donors).
- Longer follow-up may be required to examine fully any differences in PROMs between groups post-transplant.

For peer review only - http://bmjopen.bmj.com/site/about/guidelines.xhtml

For peer review only

Introduction

Healthcare usually aims to improve health and minimise disability,[1] and there is growing acknowledgment that patient-reported outcome measures (PROMs) are important for evaluating the effectiveness of healthcare. PROMs are measures of outcomes that are directly reported by the patient, and are usually questionnaires that can be generic or condition-specific. PROMs may measure a wide range of outcomes including health status, QoL, treatment satisfaction, well-being, and other outcomes such as symptoms. Despite their importance in assessing the patient's perspective, PROMs such as quality of life are not measured routinely as part of the evaluation of surgical interventions.[1] Part of the reason for this has been the belief that brief health-status tools (such as the EuroQoL-5D (EQ-5D) captures quality of life (QoL)[1], as well as the time and cost involved in collecting and interpreting the data.[2,3] However, PROMs other than health status tools have an important role in clinical care[3] as they may determine the comparative benefits of different medical interventions used to treat the same condition.[4]

One example of a condition that can benefit from the measurement of PROMs is advanced chronic kidney disease (CKD Grade (G) 5). Kidney transplantation is commonly considered to be the best medical treatment for most people with advanced CKD G5 who are fit enough for the procedure.[5] Recent changes to the kidney donor system, such as deemed consent legislation in Wales since 2015, and a similar system introduced in England in May 2020, and due to be enacted in Scotland in 2021, aim to increase the number of deceased-donor (DD) kidneys available.[6] Kidney transplant waiting times have also reduced in recent years due to an increase in unrelated living donation including altruistic donation, and kidney sharing schemes that allow for greater access to living-donor (LD) transplantation for blood-group or HLA incompatible donor-recipient pairs.[7] Transplantation success is most often measured by patient and graft survival and as such LD transplantation provides a greater survival advantage when compared directly with DD transplantation, controlling for differences in variables such as age[8] and time on dialysis.[9] LD transplantation is associated with lower rejection rates[7] and spares individuals the uncertain wait for

For peer review only - http://bmjopen.bmj.com/site/about/guidelines.xhtml

a deceased-donor organ.[7] However, it is important to note that LD recipients tend to have better clinical indices of health pre-transplant. [10,11] These potential confounding factors can impact graft survival,[12] so pre-transplant differences between LD and DD recipients should be measured and controlled, before comparisons are made between the groups. The other issue apparent with these studies is that they focus on the comparative benefits of LD and DD transplantation for health, but not for other outcomes such as QoL.

When PROMs have been assessed in kidney transplantation, cross-sectional post-transplant comparisons indicated that LD recipients reported greater social involvement and happiness compared with DD recipients.[13] When cross-sectional data are analysed, it is important to have well-matched controls but in practice that is difficult to achieve because those who are left on the waiting list for deceased-donor kidneys tend to have more health problems than those who receive a transplant. Studies that have controlled for underlying differences across the groups, such as differences in age or comorbid disease, report similar outcomes for both transplant groups on health status measures such as the SF-36,[13] although many LD recipients report experiencing feelings of guilt about the risks to their donor.[14,15] Longitudinal studies, which can include and control for baseline measures of outcomes before participants receive a transplant, suggest that improvements in health status can be seen in the first few months post-transplant for both DD and LD, but that these improvements remain stable after this time.[16-19] However, these studies lack any group with which to compare transplant recipients, such as those still awaiting transplantation, and not all include data pre-transplant. Those who are transplanted are more likely to be in better health than those patients still wait-listed for a transplant, so it is important to control for baseline differences between participants who receive a transplant and those who do not.

One major problem with of all of these studies is that the instruments used to measure quality of life (QoL) such as the SF-36, actually measure health status.[20] Health status includes aspects of a person's life such as their physical ability, daily functioning, and experience of symptoms. Health-related QoL is a term that is commonly and misleadingly used to refer to health

For peer review only - http://bmjopen.bmj.com/site/about/guidelines.xhtml

status tools such as the SF-36 and EQ-5D, which may assess health and functioning but do not assess the impact of health and functioning on QoL. In contrast, QoL as measured by the Renal Dependent QoL (RDQoL) measure used in ATTOM,[21] is defined within the questionnaire as 'how good or bad you feel your life to be'. RDQoL, modelled on the ADDQoL for diabetes[22] and influenced by McGee et al's[23] individualised interview method of generic QoL measurement, establishes the relevance of different aspects of life for each individual respondent and excludes non-relevant items from the scoring. It allows for individual differences in the importance of relevant aspects of life for QoL as well as measuring the impact of the renal condition on each relevant aspect of life. Impact is weighted by importance and an average weighted impact (AWI) score obtained for each individual indicating the nature and extent of the impact of the renal condition on the individual's QoL. A single item to measure generic QoL is also included in the measure and this item can be expected to be more strongly related to health status generally than the condition-specific AWI score.[24] Given the confusion surrounding the definitions and measurement of these concepts, it is important that research includes genuine measures of QoL and condition-dependent QoL as well as a health status measure when investigating PROMs in people with CKD. The addition of qualitative research methods alongside PROMs, can be valuable in providing more detailed insight into how transplant recipients experience transplantation and how it impacts QoL.

The main objectives of the present study were 1) to examine QoL (generic and condition-dependent), health status, and other PROMs in recipients of DD or LD transplants, and 2) to compare them over time with those on the waiting list (WL) for a DD transplant in a matched cross-sectional cohort and in a subsample with longitudinal data pre- and post-transplant. Specifically, it was hypothesised that, controlling for potential confounding factors, there would be very few differences in outcomes between DD and LD recipients. It was also hypothesised that transplant recipients would report less negatively impacted QoL and better scores on other PROMs than participants waiting for a transplant. The third objective was to use qualitative interview methods to

For peer review only - http://bmjopen.bmj.com/site/about/guidelines.xhtml

explore in more depth the expectations and experience of transplantation and how it influences QoL one-year post-transplant in DD and LD transplant recipients.

Methods

Study design, participants and procedure.

This study employed a longitudinal cohort survey design, as well as a qualitative interview approach. The study was part of the UK Access to Transplantation and Transplant Outcome Measures (ATTOM) research programme.[25] ATTOM was based on a large national observational cohort study involving all kidney units in the UK in order to examine the reasons for disparities in access to renal transplantation.[26,27] Full ATTOM methodology has been reported elsewhere.[25] The current study was conducted within a work-stream examining detailed PROMs in patients fluent in English, aged less than 75 years, receiving renal-replacement therapy (RRT) who completed measures of health status, well-being, QoL, and treatment satisfaction. Qualitative interviews were conducted with a subset of patients one-year post-transplant.

Following ethical approval (East of England Research Ethics Committee 11/EE/0120), and informed consent, participants were recruited by ATTOM research nurses to the study as incident recipients of DD transplants (*n*=104), incident LD transplant recipients (*n*=94), or patients on the waiting list for a transplant (*n*=165) and followed up either one-year post-recruitment or one-year post-transplant (Figure 1). Across 18 UK renal centres, a subset of participants recruited to ATTOM were recruited to this work-stream. The first eligible patient for each transplant group seen each month (November 2011 to March 2013) by each nurse was invited to take part. Inclusion criteria were fluency in English and <75 years of age. Participants were recruited if they had G5 CKD and received a DD or LD transplant within one month. The UK Transplant Registry identified possible matched controls for recruited transplant recipients every two weeks, and members of this list of potential participants were then invited to take part as WL patients. Participants on the WL were matched to DD transplant recipients as closely as possible in terms of renal centre, age (+/- 5 years),

For peer review only - http://bmjopen.bmj.com/site/about/guidelines.xhtml

time on the waiting list (+/-100 days), and previous type of RRT. Participants completed measures of health status and well-being when first recruited to ATTOM by the research nurses, and measures of generic and renal-dependent QoL, and renal treatment satisfaction three months post-transplant/post recruitment via telephone or post. A timeframe of three months was chosen to allow participants time to experience their treatment (i.e. transplant for those receiving a transplant) and be able to reflect on how their QoL and treatment satisfaction were impacted after their return home for those having surgery. At one year, participants were contacted via telephone and completed all measures again (via telephone or post), as well as a measure of change in treatment satisfaction which compared satisfaction with current renal treatment and previous renal treatment. One-year follow-up was chosen because it would be expected that clinical outcomes would be stable one-year post-transplant, but it's not clear whether non-clinical outcomes (i.e. PROMs) would also remain stable. At the same time, choosing one-year follow-up allows for comparisons to be made with previous research that has examined health status using the same timeline. A sample of 100 participants in each of the three groups was calculated as adequate to reach 80% power within the planned analyses. Some of the participants recruited as WL patients subsequently received a DD (*n*=41) or LD kidney transplant (*n*=26) in the year of follow-up. For these participants, the initial measures were completed while they were still on the WL, giving a pre-transplant measure, and the second set of measures was completed one-year post-transplant (Figure 1). These data allowed for secondary analyses in patients for whom we had true baseline measures comparing the two transplant groups and those remaining on the waiting list pre- and post-transplant. Participants recruited as WL comparison patients who went on to receive a transplant within ATTOM were not asked to complete the measures at 3 months post-transplant in the same way as those recruited to the study as transplant recipients were asked to do. It was not always possible to contact people within three months of receiving their transplant once they had been recruited as WL patients, and a limit of 4 points of data collection were set for each participant, to minimise participant burden.

For peer review only - http://bmjopen.bmj.com/site/about/guidelines.xhtml

A subset of transplant recipients was selected for qualitative telephone interviews. The aim of this qualitative study was to explore the experience of receiving a transplant, and how having a transplant affects QoL and other PROMs. The sample consisted of 10 DD transplant and 10 LD transplant recipients. All participants had been contacted previously by authors (AG or JB) when arranging completion of questionnaires. Participants were invited to take part based on their average weighted impact (AWI) scores on the Renal Dependent Quality of Life (RDQoL) questionnaire,[21] completed one-year post-transplant. Participants with AWI scores above or below one standard deviation of the mean score, as well as those with a mean score, were included so that participants had varying levels of QoL. The qualitative sample was selected on the basis of QoL scores to aid our understanding of the range of responses and how they may relate to variations in QoL. The profile of the qualitative-study participants reflected the age and sex profile of the larger sample. Participants were informed that the interview would explore their questionnaire responses related to their QoL and treatment satisfaction. Semi-structured telephone interviews were conducted between January and August 2014, and were conducted by a single interviewer using an interview schedule (Supplementary Data 1) guided by published literature and participants' questionnaire responses.[28] Interviews were conducted by a postdoctoral research fellow (AG) experienced in qualitative research and trained in use of NVivo software (QSR International, US) for qualitative analysis, with background in the field of health psychology. Field notes were made following every audio-recorded interview. Interview recordings (mean=52min, *SD*=13.4, range=31-76) were transcribed.

<u>Patient and Public Involvement</u>

Patients were involved in the development of the ATTOM project objectives and procedures, as well as analysis and dissemination. A patient representative from the National Kidney Federation (NKF) was involved in all aspects of the development of the research. A patient representative was also invited to all steering group meetings throughout the duration of the project. Findings were

For peer review only

presented to patients and lay people as part of an NIHR stakeholder ATTOM meeting in November 2017.

Outcomes

A summary of the primary outcome measures can be seen in Supplementary Data 2, Table 1. QoL was assessed using the RDQoL questionnaire,[21] developed from the Audit of Diabetes Dependent Quality of Life (ADDQoL) questionnaire.[22,29] It includes one overview item to measure generic QoL on a scale from excellent (+3) to extremely bad (−3). Twenty-one items measure the impact of the renal condition on specific aspects of life. Each specific item includes a rating of the impact of the renal condition on QoL, from -3 (most negative impact) to +1 (positive impact), and a rating of importance of that aspect of life for the individual's QoL ranging from very important (+3) to not at all important (0). Impact and importance ratings are multiplied to give a weighted impact score for each item, ranging from −9 (most negative/important impact of the renal condition on QoL) to +3 (most positive/important impact). Some items include preliminary questions to determine applicability to the individual (e.g. work). The weighted impact scores are summed and divided by the number of applicable items to give an average weighted impact (AWI) score, also ranging from −9 to +3. Principal axis factoring and reliability analyses confirmed that the RDQoL was appropriate for both transplant and non-transplant groups (see Supplementary Data 3, Tables 2-4).

Well-being was measured by the Well-being Questionnaire (W-BQ12).[30] Higher scores indicate greater well-being (range=36-0). Health status was measured by the EQ-5D-5L,[31] which has two sections. The first section includes five dimensions of health that are rated on five levels. These data were then converted into a population preference score called a health-utility value, using the new value set for England.[32] The second section asks participants to rate 'Your health today' on a visual analogue scale (EQ-VAS) from 100 (best health you can imagine) to 0 (worst health you can imagine). The EQ-VAS is a PROM, but the utility measure is not, as it depends not only on patients'

For peer review only - http://bmjopen.bmj.com/site/about/guidelines.xhtml

reports but also on the values assigned by the general population to each profile of scores for the five dimensions of health used to create the value set for England.

Satisfaction with current renal treatment was measured using the Renal Treatment Satisfaction Questionnaire status version (RTSQs).[33] It has 13 items, with 7-point scales (6 to 0), that are summed to give a total score (range=78-0). Higher scores indicate greater satisfaction with current renal treatment. The change version (RTSQc) is modelled on the change version of the Diabetes Treatment Satisfaction Questionnaire (DTSQc),[34] developed to counteract ceiling effects found with the status version (DTSQs).[35] It asks participants to compare satisfaction with current treatment with satisfaction with their previous treatment on 7-point scales (+3 to −3 where 0=no change) and is summed to give a total score from +39 (maximum satisfaction improvement) to -39 (maximum deterioration in satisfaction). Principal axis factoring and reliability analyses confirmed that the RTSQs and RTSQc were appropriate for both transplant and non-transplant groups (see Supplementary Data 3, Tables 5-6).

All sociodemographic and medical information was recorded by research nurses from medical notes at time of recruitment. Any condition mentioned in patients' records was identified and classified into groups (heart disease, heart failure, liver disease, diabetes, mental health problems, peripheral vascular disease). Prescribed immunosuppressant and steroid medication were also recorded. Height and weight were recorded to allow calculation of Body Mass Index (BMI). Any subsequent changes to information (such as change from being on the WL to receiving a transplant) were self-reported by participants.

<u>Analysis</u>

Reflecting previous research in this area, the main analyses involved cross-sectional differences at one-year follow-up in those who received a DD transplant (*n*=145), LD transplant (*n*=120), and those who remained waiting for a transplant (*n*=98) at one-year follow-up. These analyses were conducted using one-way Analysis of Covariance (ANCOVAs), controlling for medical

For peer review only - http://bmjopen.bmj.com/site/about/guidelines.xhtml

and demographic differences recorded at recruitment. Clinical factors such as previous renal replacement therapy, as well as demographic factors including age, and indicators of SES (such as car ownership and qualifications), can influence a person's perspective on their QoL and treatment satisfaction. We therefore assessed these variables and controlled for them in the analyses if differences between groups were identified (using Chi Squared tests or ANOVAs).[36] It was hypothesized that patients still on the WL would have more negative scores on PROMs than those who received a transplant.

Secondary analyses involved a prospective assessment of those participants recruited as patients on the WL (*n*=165). The sample was divided into three groups based on their treatment at follow-up; those who remained waiting for a transplant (*n*=98); those who subsequently had a DD (*n*=41); and those who subsequently had an LD transplant (*n*=26). This allowed for changes in outcomes to be measured pre- and post-transplant for these groups and for pre-transplant differences to be controlled for. Differences between groups and over time were investigated with a series of 3 (group) x 2 (time) ANCOVAs with planned comparisons on all PROMs and utility outcome scores (controlling for various factors). It was hypothesised that those who received a transplant would report improved PROMs, whilst those remaining on the waiting list would report either no change, or a deterioration in scores. It was also hypothesised that when differences in sociodemographic factors are controlled, there will be no differences in outcomes between DD and LD recipients. A one-way ANCOVA was also conducted to examine differences between these three groups (those who remained on the WL, those who subsequently had a DD or LD transplant) for change in satisfaction comparing treatment at one year (e.g. one-year post-transplant) with previous renal treatment (e.g. dialysis at recruitment) using RTSQc scores.

For the qualitative interview data,[37] thematic analysis based on a pragmatic and critical realist approach was used to explore the experiences of transplant recipients. Field notes were reviewed and transcripts read three times for familiarisation prior to analysis. The coding was completed in Microsoft Word, then entered into NVivo10 software for analysis. Using established

For peer review only - http://bmjopen.bmj.com/site/about/guidelines.xhtml

guidelines,[38] independent initial coding by AG established major themes derived from the data which enabled development of a coding framework (AG, CB, MC). There was substantial coder agreement (AG and JB) on 5 interviews; AG completed the remaining coding on subsequent interviews. Reiteration of responses indicated data saturation had been achieved after 20 interviews.

Results

Descriptive findings

Descriptive data for the groups at recruitment (165 patients recruited whilst on waiting list for a kidney transplant, 104 DD recipients, 94 LD recipients) are shown in Table 1. Almost 13 percent (12.5%) of DD recipients and 47.5% of LD recipients received their transplants before requiring dialysis. Almost eighteen percent (17.6%) of those on the waiting list were not yet on dialysis when recruited to the study. At recruitment, 47 (28.5%) patients on the WL had had a previous kidney transplant that failed, whilst 13 LD (13.8%) and 12 DD (11.5%) recipients had had a previous transplant.

Of all those who received a DD transplant during the study, 80 (55.2%) received a kidney from a donor after circulatory death (DCD), and 65 (44.8%) received a kidney from a donor after brain death (DBD). Sixty-nine LD recipients (57.5%) received a kidney from a relative (35 parent or adult child, 24 from a sibling and 10 from another relative e.g. aunt, uncle, niece, nephew, cousin), whilst 51 (42.5%) received a genetically unrelated donor kidney (via the paired/pooled exchange or altruistic donors).

In total, 82 (22.6%) participants did not complete measures at one year; 33 (9.1%) were not contactable, 39 (10.7%) did not return questionnaires, and 10 were known to have died (2.8%). Responders at one year were older ($t_{(120.55)}$= -2.4, p=0.017), and had better EQ-VAS health ratings ($t_{(347)}$= -2.9, p=0.005) at recruitment compared with non-responders. There were no differences in sex, employment status, civil status, education, or previous RRT between those who did and did not respond at one year. Responders at one year were more likely to be White (χ^2 =10.3, df=4, p=0.016)

For peer review only - http://bmjopen.bmj.com/site/about/guidelines.xhtml

than non-responders. Missing data were fewer than 5% for all variables and were missing at random, so cases were deleted analysis by analysis.

For those 20 participants who took part in the qualitative interviews, 10 were women, 13 were living with a partner or married, four were single, and three were separated or divorced. Nine were working, 8 were retired, and three were unemployed/long-term disabled. Six had had a previous transplant, and 17 were White, with the remaining three participants being Chinese, Asian, or identified as mixed race. Six were transplanted before requiring dialysis, 12 were previously on haemodialysis, and two received peritoneal dialysis prior to transplant.

Quantitative findings

LD recipients were significantly younger compared with the other groups ($F_{(3,375)}$=9.7, p<0.001; see Table 1). The majority of DD recipients and WL patients were on haemodialysis, whilst a larger proportion of LD recipients were transplanted before commencing dialysis (χ^2=23.3, df=2, p<0.001). LD recipients were more likely to own a car (χ^2=6.9, df=2, p=0.032). Those who remained on the WL differed from those who received a transplant on two marginally related factors (r=0.11, p=0.039); patients on the waiting list were more likely to have experience of a previous transplant that failed (χ^2=16.8, df=2; p<0.001) and experience of mental health problems (as recorded by research nurses from medical notes; χ^2=8.9, df=2; p=0.011). DD recipients and WL participants reported fewer educational qualifications than LD recipients (χ^2=13.7, df=6, p=0.033). All subsequent analyses controlled for age, previous RRT, previous experience of a transplant, education, car ownership, and history of mental health problems. Participants who were classified as obese at recruitment (BMI >30) or had a diagnosis of diabetes were more likely to experience worse QoL than those without either condition (ps<0.05). In separate research conducted as part of the ATTOM programme, obesity was associated with a greater likelihood of delayed graft function in DD transplant recipients, and diabetes was related to a greater risk of transplant failure in LD transplant recipients.[39] Analyses were therefore run controlling for obesity and diabetes. The difference in

For peer review only - http://bmjopen.bmj.com/site/about/guidelines.xhtml

generic QoL at baseline in those patients who remained on the WL and who did not go on to receive a transplant disappeared, but the sample size was reduced by 16 participants. There were no meaningful differences in the outcomes at 12m follow-up in the analyses that did and did not control for these factors, so reported findings do not control for obesity and diabetes.

Table 2 shows the correlations between the main outcome variables at 12m follow-up. Table 3 shows the cross-sectional differences at one-year post-recruitment/post-transplant between those who remained on the WL for a transplant (n=98), and those who received a DD (n=145) or LD transplant (n=120). Participants on the WL reported significantly worse scores on all PROMs at one year compared with the two transplant groups (all p<0.001). LD recipients reported better generic QoL, renal-dependent QoL, well-being, and treatment satisfaction (all p<0.05) than DD recipients. These analyses suggest that LD recipients have better outcomes than DD recipients post-transplant, but do not consider baseline differences in PROMs pre-transplant.

Table 4 and Figure 2 depict the prospective analyses comparing over time, those who remained on the WL (n=98) and those who were recruited as WL patients, but who subsequently received a LD (n=26) or DD transplant (n=41). There were significant interaction effects between the three groups and from recruitment to one-year post-recruitment/post-transplant for all PROMs. At recruitment, WL patients who did not go on to receive a transplant reported worse generic QoL than those who went on to receive DD transplants (p=0.028). Patients who remained on the WL reported worsening EQ-VAS health ratings (p=0.004), utility values (p<0.001), and well-being (p<0.001) from recruitment to one year, but generic QoL, renal-dependent QoL, and treatment satisfaction remained stable over time. In contrast, DD recipients reported improvements in EQ-VAS health ratings (p=0.017), renal-dependent QoL (p=0.011), and RTSQs scores (p<0.001), but no change in utility values (p=0.450), well-being (p=0.380), or generic QoL (p=0.200) from pre- to post-transplant. LD recipients reported improvements in all outcomes post-transplant (p<0.01). Though LD recipients reported greater treatment satisfaction than DD recipients (p=0.038), the transplant groups did not differ on any other PROMs post-transplant. Patients still on the WL reported worse scores on all

For peer review only - http://bmjopen.bmj.com/site/about/guidelines.xhtml

PROMs at one year compared with both transplant groups (p<0.001). The effect sizes ranged from small to moderate (Table 4; partial η^2 = 0.10-0.27). Sixteen percent of DD recipients and 5% of LD recipients reported worse generic QoL post-transplant. In contrast, 7% of DD recipients and no LD recipients reported more negatively impacted AWI scores from pre- to post-transplant. Similarly, 8% of DD recipients and no LD recipients reported worse health status as measured by the EQ-VAS from pre-to post-transplant.

Using the treatment satisfaction scores from the RTSQs at recruitment as a further covariate in analyses, both DD (M=29.6, SE=2.7) and LD recipient groups (M=31.8, SE=3.4) reported greater satisfaction with renal treatment at one year versus previous treatment than those still on the WL (M=14.4, SE=1.9; $F_{(1, 97)}$= 16.1, p<0.001; partial η^2 = 0.25; Figure 3).

<u>Qualitative findings</u>

Three themes were identified from the qualitative interviews; participants discussed the positive impact of transplantation, the impact of expectations on the ability to cope post-transplant, and their feelings towards donors. Illustrative examples of quotations can be seen in Table 5. Overall, participants viewed their transplant as a way to return to "normal". Both transplant groups reported physical improvements, reduced dietary and fluid restrictions, and improved lifestyle post-transplant. Many DD recipients had not yet returned to work, but some LD recipients reported improvements related to their employment, due to their increased physical ability.

Patient expectations of transplantation had a strong influence on whether or not patients felt their transplant was successful. Many patients believed that once they received a transplant, they would return to the same level of physical health they experienced prior to their kidney problems. Six out of the 10 LD recipients reported ongoing physical problems at one-year post-transplant, such as reduced stamina, which they did not anticipate. This led to more difficulty adjusting post-transplant, and lower rates of treatment satisfaction. An unexpected side effect of transplantation included changes in physical appearance, such as scarring, bloating from the surgery,

For peer review only - http://bmjopen.bmj.com/site/about/guidelines.xhtml

or weight gain from medication. Patients who experienced these changes found it harder to cope, and their QoL was negatively impacted. Other unexpected experiences, such as difficulty adapting to drinking large quantities of fluids and relearning bladder control post-transplant, were issues that participants found particularly difficult.

Although all participants reported feeling immense gratitude to their donors, LD recipients worried about the health of their donors, leading to feelings of guilt up to one-year post-transplant. This was particularly evident in three recipients whose donors were their adult children. Continued worry about their donors meant that LD recipients were likely to report difficulties in coping post-transplant.

Discussion

Previous research has shown consistently that there are differences in the patient characteristics of DD and LD recipients.[13,14,40] Controlling for such factors, our cross-sectional analyses suggest that LD transplantation conveys an advantage over DD transplantation for QoL and treatment satisfaction. However, once baseline differences in factors such as age, education, and time on dialysis were controlled for (in the subset of patients with true baseline measures pre-transplant), DD and LD kidney transplant recipients are found to have similar outcomes post-transplant. The only significant difference between transplant groups with true baseline data was in treatment satisfaction one-year post-transplant with LD recipients having greater treatment satisfaction than DD recipients. Previous research showed that LD recipients reported greater improvements within the first few months post-transplant, but LD and DD recipients did not differ after longer intervals of time.[17] Although the improvements seen in the LD recipients is encouraging, our results suggest that they have worse baseline measures of treatment satisfaction, health status and generic QoL before transplant, so their improvements post-transplant are more evident. Caution should be exercised when interpreting studies that have not controlled for differences at recruitment and rely on cross-sectional follow-up data alone.

For peer review only - http://bmjopen.bmj.com/site/about/guidelines.xhtml

The qualitative interviews show that although participants report improvements to their QoL through a return to 'normal' and improvements in lifestyle, participants still experience ongoing negative impact. Although transplantation is considered a positive and successful treatment, it cannot minimise the negative impact of a renal condition completely. The findings reflect those of previous studies, which report that some transplant recipients find it difficult to adjust and cope post-transplant.[41-43] Differing themes emerged for the LD and DD recipients; Although slightly more LD recipients had returned to work by one year (5/10 LD vs 3/10 DD), they were more likely to mention differences in adapting post-transplant and feel that their positive expectations for recovery and return to work were not realised. Most LD recipients were not on dialysis before they received their transplant, so the benefits they perceived from transplantation were fewer than those who have experienced dialysis. It is important to manage patient expectations (e.g. recovery times) to avoid disappointment and promote effective coping with physical and psychosocial changes following transplant. A small number of LD recipients reported experiencing feelings of guilt about the risks to their donors.[13,14] Further support may be required for those considering such a transplant, to help anticipate and manage the potential for the feelings of guilt which may include deciding to decline the offer. The impact of transplantation on QoL and other PROMs on both recipients and their donors need to be considered, especially when assessing the comparative benefit of LD versus DD transplantation.

In line with our hypotheses, WL patients reported worse health and well-being over time. This may in part be due to the fact that almost 18% of those on the WL were not yet on dialysis when recruited to the study but all required dialysis one year later. Some patients may find that their health improves once they commence dialysis, but on average health status declined. However, QoL and treatment satisfaction scores did not change over the course of 12 months in those on the WL despite their deteriorating health status and well-being. This study cannot explain in detail the whole experience of patients on the WL for a transplant, and due to the relatively small sample size and small effect sizes for some of the outcomes, some of the results may not have been adequately

For peer review only - http://bmjopen.bmj.com/site/about/guidelines.xhtml

powered. The lack of differences between groups over time therefore needs to be considered with caution. Despite this, the results highlight differences between health status and QoL.[20] Quality of life can be affected by multiple aspects of people's lives with their health being just one aspect, so it is important to distinguish between measures of genuine QoL and health status when selecting and interpreting the results from PROMs. This is particularly the case where the treatment for long-term conditions such as CKD may benefit health status but may impair QoL.

There are some limitations to this study. The sample of transplant participants with pre-transplant data was relatively small (n=67), as they were part of secondary analyses, some of which may be underpowered, so caution needs to exercised when drawing conclusions. However, the findings provide insight into how QoL and other patient-reported outcomes change over time in transplant recipients. Only those fluent in English were recruited to ensure that participants could complete the questionnaires: cost of linguistic validation for all the PROMs to be translated into other languages exceeded the ATTOM programme's resources. This meant that, for example, few people of South Asian descent took part in our sub-study, despite the fact that approximately 9.4% of transplant recipients recruited to the larger ATTOM research programme were of Asian ethnicity (with the great majority being of South Asian descent). Those interviewed for the qualitative interviews were mainly White. Therefore, the sample is less ethnically diverse than the UK renal population as a whole. Patients who responded at one year were more likely to be older, White, and have better health ratings at recruitment, so our findings may not be generalizable to younger non-White patients, and may give a more positive view of changes in health over time than if those with worse baseline health status were included. Ten patients were known to have died during the study period and no one reported a failed transplant during data collection, limiting the scope of the findings to those who remained healthy enough to be on the WL, and those with a functioning graft. Despite this, participants to our PROMs sub-study were recruited as part of ATTOM from renal units across the UK, reflecting the full ATTOM sample in terms of age, sex, previous transplant, and rate of pre-emptive transplantation. Most patients had not returned to work at follow-up; those who had

For peer review only - http://bmjopen.bmj.com/site/about/guidelines.xhtml

returned to work had varying levels of QoL compared to those who had yet to return to work. The long-term impact of taking immunosuppressants, including the increased risk of cancer and other diseases, may not yet be reflected in participants' QoL and other PROMs within the first year of having a transplant. Longer follow-up, therefore, may be required to show any differences across transplant groups, and the long-term impact of immunosuppressant medication on QoL. The focus of this study was on PROMs, but medical variables such as the number of complications post-transplant which may impact on QoL, were not assessed. Nevertheless, this is one of the few studies that provide any pre-transplant data, allowing us to examine changes over time in transplant recipients and, importantly, to control for baseline differences in PROMs. Additionally, the inclusion of WL patients along with recipients of LD and DD in analyses which controlled for group differences, allowed for direct comparison of outcomes for transplant recipients and those still on the WL. The paper highlights the importance of measuring and controlling for pre-existing differences between groups. A strength of this study is that it included both qualitative and quantitative data, which allowed for further understanding of the subjective experience of transplantation. It also shows that examining a range of PROMs provides a much better understanding of the experience of waiting for or receiving a transplant than is provided by health-status measures alone.

These findings have implications for clinical practice including future monitoring of patient-reported outcomes. Both transplant groups reported better outcomes than those still on the WL for a transplant, but there were few differences in PROMs between groups post-transplant after adjustment for potential confounders. Living donation has certain medical advantages, including that recipients do not have to wait as long for the transplant, but it is also important to acknowledge that living donation doesn't just affect recipients and their donors physically. Discussion is needed about more than just the medical benefits for the recipient and the medical impact on the donor; PROMs need to be better integrated into the information provided to recipients and donors when transplantation is discussed as a treatment option. Knowing that not all patients report improvements in PROMs and why may help future patients avoid disappointment. Routine

For peer review only - http://bmjopen.bmj.com/site/about/guidelines.xhtml

collection of PROMs data at time of wait-listing and periodically thereafter is required so that a more accurate picture of changes pre- to post-transplant can be achieved from larger more representative datasets. Careful expectation management, information on recovery time, anticipated physical changes (including scar size) and side-effects may help avoid disappointment and ensure better informed decisions about transplantation options. Nevertheless, both transplant types were, overall, more beneficial than remaining on the WL and may be considered similarly advantageous in improving QoL and other patient-reported outcomes.

For peer review only

For peer review only - http://bmjopen.bmj.com/site/about/guidelines.xhtml

Contributorship statement:

AG contributed to the design of the qualitative study, developed the interview schedule, conducted the interviews, developed the coding framework, coded and analysed the quantitative and qualitative data, drafted the manuscript, and edited and approved the final submission. JB managed telephone and postal data collection, coded qualitative data, provided feedback on initial drafts of the manuscript, and edited and approved the final submission. MC contributed to the design of the qualitative study, development of the interview schedule and coding framework, provided feedback on initial drafts of the manuscript, and edited and approved the final submission. HD contributed to the design of the qualitative study, development of the interview schedule, and edited and approved the final submission. RJJ, GCO, RR, CRVT, PR, WM, JLRF, and CD contributed to the design, organization and conduct of the wider ATTOM programme including data collection for this sub-study, and provided feedback on the interview schedule, and edited and approved the final submission. JAB, RR, GCO, CJEW, CRVT, CD and JLRF conceived and designed the ATTOM programme, contributed to the design of the present studies, provided feedback on the interview schedule, and edited and approved the final submission. CB contributed to the design of the quantitative and qualitative studies, analysis planning and interpretation of results, development of the interview schedule and coding framework, edited early drafts of the manuscript, and edited and approved the final submission. AG and CB act as guarantor, and accept full responsibility for the work and/or the conduct of the study, had access to the data, and controlled the decision to publish. The corresponding author attests that all listed authors meet authorship criteria and that no others meeting the criteria have been omitted. We thank the patient advisers for their contributions to the planning of ATTOM and discussions of findings.

Competing interests: All authors completed the ICMJE uniform disclosure form at www.icmje.org/coi_disclosure.pdf. Prof Watson reports personal fees from Glaxo Smith Kline outside the submitted work. Prof. Clare Bradley reports grants from NIHR during the conduct of the

For peer review only - http://bmjopen.bmj.com/site/about/guidelines.xhtml

study, and grants from NIHR and GlaxoSmithKline/ViiV Healthcare, outside the submitted work. CB is the majority shareholder in a company, Health Psychology Research Ltd, which licenses her patient-reported outcome measures, for others to use. These questionnaires include the RDQoL, RTSQ, and W-BQ used in the ATTOM programme. CB owns the copyright in all of these instruments and when they are licensed for use by commercial companies in their clinical trials, receives royalties. All other authors declared no competing interests.

The results presented in this paper have not been published previously in whole or part, except in abstract format.

Funding: This article presents independent research funded by the National Institute for Health Research (NIHR) under the Programme Grants for Applied Research scheme (RP-PG-0109-10116). The views expressed are those of the authors and not necessarily those of the NHS, the NIHR or the Department of Health.

Data sharing: Data is available upon reasonable request.

Ethical approval: IRAS project ID: 68259. REC reference 11/EE/0120

AG and CB affirm that the manuscript is an honest, accurate, and transparent account of the study being reported; that no important aspects of the study have been omitted; and that any discrepancies from the study as originally planned (and, if relevant, registered) have been explained.

The Corresponding author has the right to grant on behalf of all authors and does grant on behalf of all authors, a worldwide licence to the Publishers and its licensees in perpetuity, in all forms, formats and media (whether known now or created in the future), to i) publish, reproduce, distribute, display and store the Contribution, ii) translate the Contribution into other languages, create adaptations,

For peer review only - http://bmjopen.bmj.com/site/about/guidelines.xhtml

reprints, include within collections and create summaries, extracts and/or, abstracts of the Contribution, iii) create any other derivative work(s) based on the Contribution, iv) to exploit all subsidiary rights in the Contribution, v) the inclusion of electronic links from the Contribution to third party material where-ever it may be located; and, vi) licence any third party to do any or all of the above.

For peer review only

For peer review only - http://bmjopen.bmj.com/site/about/guidelines.xhtml

References

1. Black N. Patient reported outcome measures could help transform healthcare. *BMJ* 2013; 346: 1167 https://doi.org/10.1136/bmj.f167
2. Tajima, R., Kondo, M., Kai, H. et al. Measurement of health-related quality of life in patients with chronic kidney disease in Japan with EuroQol (EQ-5D). *Clin Exp Nephrol* 2010; 14: 340–348 https://doi.org/10.1007/s10157-010-0304-1
3. Boyce MB, Browne JP, Greenhalgh J. The experiences of professionals with using information from patient-reported outcome measures to improve the quality of healthcare: A systematic review of qualitative research. *BMJ Qual Saf* 2014; 23:508-518.
4. Coulter A. Measuring what matter to patients. *BMJ* 2017; 356 doi: https://doi.org/10.1136/bmj.j816
5. MacNeill SJ, Ford D. UK Renal Registry 19th Annual Report: Chapter 2 UK Renal Replacement Therapy Prevalence in 2015: National and Centre-specific Analyses/ Nephron 2017; 137(suppl):45-72.
6. Kidney Advisory Group on behalf of NHSBT. Kidney Transplantation: Deceased Donor Organ Allocation. 2019. Policy POL186/10. Available from https://nhsbtdbe.blob.core.windows.net/umbraco-assets-corp/16915/kidney-allocation-policy-pol186.pdf
7. NHS Blood and Transplant. Annual Report on Kidney Transplantation 2016/17. 2017. Available from https://nhsbtdbe.blob.core.windows.net/umbraco-assets-corp/4607/kidney-annual-report-2016-17.pdf. [Accessed 23rd October 2017].
8. Nemati E, Einollahi B, Pezeshki ML, Porfarziani V, Fattahi MR. Does kidney transplantation with deceased or living donor affect graft survival? *Nephrol Urol Mon* 2014; 6(4):e12182. doi: 10.5812/numonthly.12182
9. Gjertson DW, Cecka JM. Living unrelated donor kidney transplantation. *Kidney International* 2000; 58:491-499.

For peer review only - http://bmjopen.bmj.com/site/about/guidelines.xhtml

10. Wu DA, Robb ML, Watson CJE, et al. Barriers to living donor kidney transplantation in the United Kingdom: a national observational study. *Nephrol Dial Transplant* 2017; 1;32(5):890-900.

11. Molmenti EP, Alex A, Rosen L, et al. Recipient criteria predictive of graft failure in kidney transplantation. *Int J Angiol* 2016; 25:29-38.

12. Gozdowska J, Zatorski M, Torchalla P, et al. Living-donor versus deceased-donor kidney transplantation: Comparison of psychosocial consequences for recipients. *Transplant Proc* 2016; 38:1498-1505.

13. Ware JE, Sherbourne CD. The MOS 36-item short-form health survey (SF-36). *Med Care* 1992; 30:473-483.

14. Griva K, Ziegelman JP, Thompson D, et al. Quality of life and emotional responses in cadaver and living related renal transplant recipients. *Nephrol Dial Transplant* 2002; 17: 2204-2211.

15. Zimmermann T, Pabst S, Bertram A, Schiffer M, de Zwaan M. Differences in emotional responses in living and deceased donor kidney transplant patients. *Clin Kidney J* 2016; 9(3): 503-509.

16. Costa-Requena G, Cantarell MC, Moreso F, Parramon G, Seron D. Calidad de vida relacionada con la salud en el trasplante renal: seguimiento longitudinal a 2 años. *Med Clin (Barc)* 2017; 49:114-118.

17. Griva K, Stygall J, Ng JH, Davenport A, Harrison MJ, Newman S. Prospective changes in health-related quality of life and emotional outcomes in kidney transplantation over 6 years. *J Transplant* 2011; 671571.

18. Lønning K, Heldal K, Bernklev T, et al. Improved health-related quality of life in older kidney recipients 1 year after transplantation. *Transplant Direct* 2018; 4: e351; doi: 10.1097/TXD.0000000000000770

19. Suzuki A, Kenmochi T, Maruyama M, et al. Changes in quality of life in deceased versus living-donor kidney transplantations. *Transplant Proc* 2012; 44:287-289.

For peer review only - http://bmjopen.bmj.com/site/about/guidelines.xhtml

20. Bradley C. Importance of differentiating health status from quality of life. The Lancet 2001; 357: 7-8. doi: 10.1016/S0140-6736(00)03562-5

21. Bradley C. Design of a Renal-Dependent Individualized Quality of Life Questionnaire. *Adv Perit Dial* 1997; 13: 116-120.

22. Bradley C, Todd C, Gorton T, et al. The development of an individualised measure of perceived impact of diabetes on quality of life: The ADDQoL. *Qual Life Res* 1999; 8(1-2): 79-91.doi:10.1023/A:1026485130100.

23. McGee HM, O'Boyle CA, Hickey A, O'Malley K, Joyce CRB. Assessing the quality of life of the individual: the SEIQoL with a healthy and a gastroenterology unit population. *Psychol Med* 1991; 21: 749-59.

24. Bradley C, Eschwège E, de Pablos-Velasco P, Parhofer KG, Simon D, Vandenberghe H et al. Predictors of quality of life and other patient-reported outcomes in the PANORAMA multinational study of people with type 2 diabetes. *Diabetes Care* 2018; 41(2):267-276.

25. Oniscu GC, Ravanan R, Wu D, et al. Challenges and opportunities in renal transplantation: Access to Renal Transplantation and Transplant Outcome Measures (ATTOM) study. *BMJ Open* 2016; 6:e010377.

26. Ravanan R, Udayaraj U, Ansell D, et al. Variation between centres in access to renal transplantation in UK: longitudinal cohort study. *BMJ* 2010; 341: c3451

27. Oniscu GC, Schalkwijk AAH, Johnson RJ, et al. Equity of access to renal transplant waiting list and renal transplantation in Scotland: cohort study. *BMJ* 2003; 327:1261.

28. Louis ON, Sankar P, Ubel PA. Kidney transplant candidates' views of the transplant allocation system. *J Gen Intern Med* 1997; 12: 478-484.

29. Wee HL, Tan CE, Goh SY, Li SC. Usefulness of the Audit of Diabetes-Dependent Quality-of-Life (ADDQoL) Questionnaire in patients with diabetes in a multi-ethnic Asian country. *Pharmacoeconomics* 2006; 24(7): 673-682.

For peer review only - http://bmjopen.bmj.com/site/about/guidelines.xhtml

30. Riazi A, Bradley C, Barendse S, Ishii H. Development of the Well-being Questionnaire short-form in Japanese: The W-BQ12. *Health Qual Life Out* 2006; 4:40. doi: 1 0.1186/1477-7525-4-40

31. Herdman M, Gudex C, Lloyd A, et al. Development and preliminary testing of the new five-level version of the EQ-5D (EQ-5D-5L). *Qual Life Res* 2011; 20(10):1717-1736. doi: 10.1007/s11136-011-9903-x

32. Feng Y, Devlin N, Shah K, et al. New methods for modelling EQ-5D-5L value sets: An application to English data. Office of Health Economics, Research Paper 16/2 January 2016; London. Available from https://www.ohe.org/publications/new-methods-modelling-eq-5d-5l-value-sets-application-english-data. [Accessed 20th April 2017].

33. Barendse SM, Speight J, Bradley C. The Renal Treatment Satisfaction Questionnaire (RTSQ): A measure of satisfaction with treatment for chronic kidney failure. *Am J Kidney Dis* 2005; 45: 572-579.

34. Howorka K, Pumprla J, Schlusche C, et al. Dealing with ceiling baseline treatment satisfaction level in patients with diabetes under flexible, functional insulin treatment: Assessment of improvements in treatment satisfaction with a new insulin analogue. *Qual Life Res* 2000; 9: 915-930.

35. Bradley C. The Diabetes Treatment Satisfaction Questionnaire: DTSQ. In Bradley C. (Ed). Handbook of Psychology and Diabetes: A guide to psychological measurement in diabetes research and practice. Chur Switzerland: Harwood Academic Publishers, 1994; 111-132.

36. Field A. Discovering statistics. London. Sage 2009.

37. O'Brien BC, Harris IB, Beckman TJ, Reed DA, Cook DA. Standards for reporting qualitative research: a synthesis of recommendations. *Acad Med* 2014; 89(9):1245-1251.

38. Braun V, Clarke V. Using thematic analysis in psychology. *Qual Res Psychol* 2006; 3:77-101.

For peer review only - http://bmjopen.bmj.com/site/about/guidelines.xhtml

39. Wu DA, Robb ML, Forsythe JLR, Bradley C, Cairns J, Draper H, et al. Recipient comorbidity and survival outcomes after kidney transplantation: a UK-wide prospective cohort study. *Transplantation* 2019, Aug 19. doi: 10.1097/TP.0000000000002931.

40. Guimarães J, Araújo AM, Santos F, Nunes CS, Casal M. Living-donor and deceased-donor renal transplantation: Differences in early outcome - A single-center experience. *Transplantation Proceedings* 2015; 47:958-962.

41. Schipper K, Abma TA, Koops C, et al. Sweet and sour after renal transplantation: A qualitative study about the positive and negative consequences of renal transplantation. *Br J Health Psychol* 2014; 19: 580-591.

42. Fallon M, Gould D, Wainwright SP. Stress and quality of life in the renal transplant patient: A preliminary investigation. *J Adv Nurs* 1997; 25: 562-570.

43. Baines LS, Joseph JT, Jindal RM. Emotional issues after kidney transplantation: A prospective psychotherapeutic study. *Clin Transplant* 2002; 16: 455-460.

Table 1. Summary of baseline demographic characteristics of groups at recruitment.

	Wait-list group (*N*=165)	DD recipients (*N*=104)	LD recipients (*N*=94)	
Variable	*M (SE)*	*M (SE)*	*M (SE)*	*p*
Age[a] in years	50.7 (1.6)	51.1 (1.3)	43.9 (1.4)	<0.001
Time on waiting list in days	980 (124.9)	953 (284.9)	990 (414.4)*	0.995
Sociodemographic variables	*N (%)*	*N (%)*	*N (%)*	*p*
Age[a]				<0.001
18-34	25 (15.1)	12 (11.5)	29 (30.9)	
35-49	46 (27.9)	32 (30.8)	32 (34.0)	
50-64	66 (40.0)	41 (39.4)	27 (28.7)	
65-75	28 (17.0)	19 (18.3)	6 (6.4)	
Sex: Female	59 (35.8)	40 (38.5)	34 (36.2)	0.949
Ethnicity				0.411

For peer review only - http://bmjopen.bmj.com/site/about/guidelines.xhtml

White	134 (81.2)	84 (80.8)	85 (90.4)	
Black	15 (9.1)	10 (9.6)	5 (5.3)	
Asian	1 (0.6)	1 (1.0)	0 (0.0)	
Chinese	13 (7.9)	7 (6.7)	3 (3.2)	
Mixed	2 (1.2)	2 (1.9)	1 (1.1)	
Marital status				0.462
Single	32 (19.4)	20 (19.2)	27 (28.7)	
Married/Living with partner	98 (59.4)	63 (60.6)	60 (63.8)	
Divorced/separated/widowed	35 (21.2)	121 (20.2)	7 (7.5)	
Education				0.166
No qualifications[b]	32 (19.4)	16 (15.4)	8 (8.5)	0.033
Basic (GCSE/A level/NVQ 1-3)	94 (57.0)	66 (63.5)	68 (72.3)	
Higher (Degree/higher degree/NVQ 4-5)	39 (23.6)	22 (21.1)	18 (19.2)	
Car ownership: yes	134 (81.2)	89 (85.6)	90 (95.7)	<0.000
Clinical Variables				
Primary renal diagnosis				0.515
Diabetes: Type 1 or 2	9 (5.5)	11 (10.6)	5 (5.3)	
Glomerulonephritis	50 (30.3)	32 (30.8)	42 (44.7)	
Interstitial nephritis/pyelonephritis	21 (12.7)	13 (12.5)	16 (17.0)	
Hypertension/large vessel disease	10 (6.1)	8 (7.7)	0 (0.0)	
Cystic/hereditary/congenital disease	47 (28.4)	19 (18.3)	14 (14.9)	
Other conditions	28 (17.0)	21 (20.1)	17 (18.1)	
Comorbid conditions				
Diabetes	20 (12.2)	16 (15.4)	8 (8.5)	<0.005
Heart disease	19 (11.6)	6 (5.8)	7 (7.4)	0.006
Heart failure	6 (3.7)	4 (3.8)	0 (0.0)	0.305
Liver disease	4 (2.4)	3 (2.9)	1 (1.1)	0.299
Mental health problems	14 (8.5)	2 (1.9)	5 (5.3)	0.022

For peer review only - http://bmjopen.bmj.com/site/about/guidelines.xhtml

For peer review only

Obesity (BMI >30)	32 (19.5)	22 (21.2)	14 (14.9)	0.600
Previous Renal replacement therapy				<0.001
No dialysis[c]	29 (17.6)	13 (12.5)	43 (45.7)	
Peritoneal dialysis (PD)	27 (16.4)	23 (22.1)	14 (14.9)	
Haemodialysis (HD)[d]	109 (66.0)	68 (65.4)	37 (39.4)	
Central venous catheter (CVC)	35 (53.1)	31 (55.6)	21 (56.8)	
Arteriovenous fistula (AVF)	22 (33.3)	28 (41.2)	15 (40.5)	
Unknown	9 (13.6)	9 (13.2)	1 (2.7)	
Previous transplant failure[e]	47 (28.5)	12 (11.5)	13 (13.8)	0.001
Highly sensitized: yes	8 (4.9)	9 (8.7)	8 (8.5)	0.842
Donor related to patient: yes	-	-	56 (59.6)	
Induction suppression				0.116
None	-	16 (15.4)	19 (20.2)	
Anti-thymocyte Globulin (ATG)	-	7 (6.7)	1 (1.1)	
Basiliximab	-	66 (63.5)	53 (56.4)	
Campath	-	15 (14.4)	21 (1.1)	
Calcineurin inhibitor (CNI) maintenance				0.073
Tacrolimus	-	98 (94.2)	93 (98.9)	
Ciclosporine	-	6 (5.8)	1 (1.1)	
Anti-proliferative drugs				0.341
None	-	13 (12.5)	8 (8.5)	
Mycophenolate	-	70 (67.3)	54 (57.4)	
CellCept	-	8 (7.7)	16 (17.0)	
Myfortic	-	7 (6.7)	10 (10.6)	
Azathioprine	-	6 (5.8)	6 (6.4)	
Steroid maintenance plan				0.193
None	-	9 (8.7)	12 (12.8)	
Withdraw within 3 months	-	18 (17.3)	24 (25.5)	

For peer review only

For peer review only - http://bmjopen.bmj.com/site/about/guidelines.xhtml

Long-term use	-	77 (74.0)	58 (61.7)

Notes. *Figures here reflect only those who were on the waiting list (WL) for a DD transplant before receiving a LD transplant (n=16). [a]LD recipients were younger. [b]Fewer LD recipients reported no educational qualifications. [C] A larger proportion of LD recipients were transplanted pre-emptively. [d]More DD recipients and WL patients reported being on HD prior to transplantation/listing. [e]WL patients reported more previous transplant failures.

For peer review only

Table 2. Correlations between outcome measures 12-month follow-up for all participants.

	Variable	*1*	*2*	*3*	*4*	*5*	*6*
**1**	Generic QoL	-					
**2**	Renal-dependent QoL	0.499***	-				
**3**	Well-being	0.740***	0.551***	-			
**4**	Health status (EQ-VAS)	0.725***	0.415***	0.652***	-		
**5**	Health-utility values	0.674***	0.444***	0.678***	0.612***	-	
**6**	Renal treatment satisfaction (RTSQs)	0.545***	0.420***	0.521***	0.454***	0.364***	-
**7**	Change in treatment satisfaction (RTSQc)	0.282***	0.134*	0.292***	0.293***	0.125*	0.617***

Note. *p<0.05, **p<0.01, ***p<0.001

For peer review only

Table 3. Summary of adjusted means, standard errors, and main effects examining cross-sectional differences in groups in patient-reported outcomes at one year, controlling for age, previous renal replacement therapy (RRT), previous experience of a transplant, education, car ownership, and mental health problems.

	Wait-list group		***DD Transplant***		***LD Transplant***					
	M (SE)	*CI*	*M (SE)*	*CI*	*M (SE)*	*CI*	*F*	*df*	*p*	Partial η^2
Generic QoL[a,b]	0.4 (0.1)	0.1-0.7	1.4 (0.1)	1.1-1.6	1.8 (0.1)	1.5-1.9	27.6	266	<0.001	0.20
Renal-dependent QoL [a,b]	-4.4 (0.3)	-4.9 to -3.9	-2.7 (0.2)	-3.1 to -2.4	-1.9 (0.2)	-2.3 to -1.5	27.2	266	<0.001	0.18
Well-being[a,]	19.0 (0.9)	14.3-20.8	24.9 (0.7)	23.7-26.3	27.4 (0.8)	25.8-28.9	24.7	265	<0.001	0.17
Health status (EQ-VAS)[a]	62.4 (2.2)	58.0-66.8	77.9 (1.6)	74.8-81.2	82.4(1.9)	78.7-86.1	24.1	263	<0.001	0.18
Health-utility values[a]	0.7 (0.1)	0·6-0.8	0.8 (0.1)	0.8-0.9	0.9 (0.1)	0.8-0.9	13.1	259	<0.001	0.11
Renal treatment satisfaction (RTSQs) [a,b]	58.6 (1.3)	56.1-61.1	67.4 (0.9)	65.5-69.2	72.6 (1.1)	70.5-74.7	34.3	265	<0.001	0.21
Change in treatment satisfaction (RTSQc)[a]	14.1 (1.7)	10.8-17.4	29.2 (1.3)	26.7-31.7	30.9 (1.4)	28.2-33.8	33.6	253	<0.001	0.22

Note. DD = deceased-donor transplant, LD = living-donor transplant. *M*=mean, *SE*=standard error. EQ-VAS=Visual Analogue Scale; RTSQs=Renal Treatment Satisfaction Questionnaire status version; RTSQc=Renal Treatment Satisfaction Questionnaire change version. Partial η^2 is a measure of effect size that

For peer review only - http://bmjopen.bmj.com/site/about/guidelines.xhtml

measures the proportion of the total variance in a dependent variable that is associated with the membership of different groups defined by an independent variable, in which the effects of other independent variables and interactions are partialled out. Partial η^2 is seen as giving small (0.01) medium (0.09), or large (0.25) effect sizes.

[a]Wait-list (WL) group reported worse outcomes compared with DD or LD recipients (*ps*<0.001).

[b]LD recipients reported better scores than DD recipients (*ps*<0.05).

For peer review only

Table 4. Summary of adjusted means, standard errors, and interaction effects examining differences in those who remained on the waiting list and those who subsequently had a LD or DD transplant, in patient-reported outcomes from pre- to 12-months post-transplant/recruitment to 12 months, controlling for age, previous renal replacement therapy (RRT), previous experience of a transplant, education, car ownership, and mental health problems.

		Generic QoL	Renal-dependent QoL	Well-being	Health status EQ-VAS scores	Health-utility values	Renal treatment satisfaction (RTSQs)
Recruitment							
WL	*M (SE)*	0.39 (0.15)	-4.29 (0.27)	22.94 (0.78)	69.32 (2.63)	0.78 (0.03)	58.43 (1.76)
	CI	0.10-0.69	-4.82 to -3.75	21.40-24.48	64.10-74.53	0.73-0.83	54.94-61.93
DD	*M (SE)*	1.11 (0.21)	-3.98 (0.39)	23.18 (1.04)	67.42 (3.50)	086 (0.03)	60.10 (2.42)
	CI	0.69-1.52	-4.74 to -3.12	21.11-25.25	60.48-74.36	0.79-0.92	55.29-64.91
LD	*M (SE)*	0.77 (0.27)	-4.08 (0.49)	22.37 (1.40)	61.10 (4.27)	0.86 (0.04)	54.76 (3.15)
	CI	0.23-1.31	-5.06 to -2.15	19.59-25.15	51.78-70.42	0.77-0.94	48.50-61.01
12m post-recruitment / 12m post-transplant							
WL	*M (SE)*	0.39 (0.14)	-4.45 (0.27)	18.95 (0.99)	61.89 (2.39)	0.69 (0.03)	59.11 (1.45)
	CI	0.11-0.68	-4.99 to -3.91	16.99-20.92	57.16-66.63	0.63-0.75	56.24-61.99
DD	*M (SE)*	1.30 (0.20)	-2.92 (0.39)	24.45 (1.33)	77.19 (3.18)	0.85 (0.04)	66.07 (1.99)
	CI	0.90-1.69	-3.68 to -2.15	21.81-27.09	70.88-83.49	0.76-0.93	62.12-70.03

For peer review only - http://bmjopen.bmj.com/site/about/guidelines.xhtml

LD	*M (SE)*	1.77 (0.26)	-2.41 (0.49)	27.30 (1.79)	83.90 (4.27)	0.93 (0.05)	74.07 (2.59)
	CI	1.25-2.28	-3.39 to –1.43	23.74-30.84	75.43-92.37	0.82-1.03	68.93-79.21
	F	5.91	12.08	14.43	20.63	8.04	15.25
	df	103	102	111	110	108	101
	p	0.004	<0.001	<0.001	<0.001	0.001	<0.001
	partial η^2	0.10	0.19	0.21	0.27	0.13	0.23

Note. WL= those who remained on the waiting list from recruitment to 12m post-recruitment, DD=those recruited as WL patients, but who subsequently had a deceased-donor transplant, LD= those recruited as WL patients, but who subsequently had a living-donor transplant, *M*=mean, *SE*=standard error, CI=confidence interval: upper and lower limits. EQ-VAS=Visual Analogue Scale; RTSQs=Renal Treatment Satisfaction Questionnaire status version.

For peer review only - http://bmjopen.bmj.com/site/about/guidelines.xhtml

Table 5. Summary of qualitative themes with illustrative quotations.

Theme	Illustrative quotations
Positive impact of transplantation	**Physical changes:**<br>▪ Uh, I feel a hell of a lot better. I was terribly, terribly ill, and I even look like me now. I didn't look like me for quite a long time. I had yellow eyes and grey skin. There's no doubt at all that it's made a massive positive difference. *Woman, non-related-LD transplant following HD.*<br>▪ My energy levels are amazing… my mates and that, they just couldn't believe it. They said 'god you look better than I do'. And I was full of life, I'm full of life. That's the sort of euphoria that you need, that gradually, very gradually subsided to normality I suppose. But I was, I was almost hyper. I felt so good. *Man, pre-emptive related-LD transplant.*<br>**Lifestyle:**<br>▪ Everything's gone right back to normal now it's fine… You go back to practically normal. *Man, DD transplant following HD.*<br>▪ I feel a bit normal again you know yeah *Woman, DD transplant following APD*.<br>▪ I'm back to how I was some years back. *Woman, pre-emptive non-related-LD transplant.*<br>▪ It gives you more freedom to, to actually live the life that I wanted to live, before dialysis… me and my wife are planning our honeymoon because I missed that because I just hit dialysis after my wedding. So now… after all these years on dialysis, we can plan for the future. *Man, DD transplant following HD.*<br>▪ Being able to work is massive for me and I can now work full-time and not only work full-time but own my own home which I wasn't able to do before. *Woman, non-related-LD transplant following HD.*<br>▪ I can just get up and do things without worrying… It is so much better than what it was before. Before I thought I was going to die, now ok I know I'm going to die but maybe hopefully in the future. So, I don't worry about the little things anymore. *Woman, DD transplant following HD.*<br>▪ I mean I can drink as much as I like now. That before you could only drink like a litre a day and that. *Woman, DD transplant following HD.*<br>▪ I mean I've gone back; I have a normal diet now. *Man, non-related-LD transplant following APD.*

For peer review only - http://bmjopen.bmj.com/site/about/guidelines.xhtml

Impact of expectations on ability to cope post-transplant	**Physical changes:**<br>▪ I have to drink like a fish. So, I'm all bloated … all I've got to show for (the transplant) is a huge stomach, because obviously they fitted a kidney in and the bag in my bladder so I look like I'm pregnant all the time… I'm a bit of a recluse actually. I just stay in all the time or if I was going out I can't, I can't wear anything fitted anymore because I look pregnant. Just, it's really, rubbish! Woman, *pre-emptive DD transplant.*<br>▪ I've lots of marks on my face (from a reaction to the medication) which isn't the greatest thing for confidence in the world. *Man, related-LD transplant following HD.*<br>▪ I'm on steroids so I have put on a bit of weight. *Man, pre-emptive DD transplant.*<br>▪ There's a thing when you have a transplant that you tend to pile it on, pile the weight on quite badly. *Woman,* non-*related LD transplant following HD.*<br>▪ You have to take a lot of water … after your transplant. You're supposed to take two litres every day…I find I'm running to the toilet a lot. It breaks your sleep at night, you're up maybe three times during the night and you're waking up and you've not really properly slept. *Man, pre-emptive related- LD transplant.*<br>▪ I hadn't used my bladder for seven years, and it was pretty darn painful (to use it again) and took a lot of running to the loo every five minutes. *Woman, DD transplant following HD.*<br>▪ Then oh god I was weeing for Britain! You know, it was, that was something I found very hard to get used to because as you know your bladder would have shrunk to nothing … when I had the transplant, I had to drink six litres. I found that quite hard. *Woman, DD transplant following HD.*<br>▪ When you're on dialysis you dream of being able to drink. Once you're told you have to drink its actually really difficult…. It's becomes normal not to drink anything and then all of a sudden, you're told to drink at least 3, 4 litres of water or fluid a day. *Woman, non-related-LD transplant following HD.*<br>▪ I'm still a little bit sort of behind where I would be but I've got like 30, I've got 35% function which is a little bit beyond where I need to be to be feeling properly sort of strong and active. But I've had a year of it being sort of, it's never been really bad but I've had a year of it being not quite where it needs to be so I'm a lot more tired than I'd like to be. *Man, DD transplant following HD.*

For peer review only - http://bmjopen.bmj.com/site/about/guidelines.xhtml

	▪ I've just not got the stamina I had before like you know. *Man, non-related-LD transplant following APD.*<br>▪ I know it's taken me a whole year to really be as I was before and I still feel in some ways I'm not quite back to what I was before. *Woman, pre-emptive non-related-LD.*<br>▪ My metabolism seems to have changed a great deal from the moment I had the transplant. *Woman, related-LD transplant following HD.*<br>▪ I've always wanted to try out cabin crewing before but now I can't even think of doing it because of my health conditions cos I can deteriorate if I start to fly. I have to be very cautious about what I want, you know like what I choose now, just to have to be very careful basically, even holidays. It's just very depressing. *Woman, DD transplant following PD.*
Feelings towards donor	**Gratitude:**<br>▪ I'm so grateful for having it. *Man, DD transplant following HD.*<br>▪ I'm very lucky to get the chance, due to my donor like; you know to get a chance of living again, and being without dialysis. *Woman, DD transplant following PD.*<br>▪ I mean I really, I'm incredibly, incredibly lucky I've got this person who I don't know, somewhere in the world, somewhere in the UK, I don't know where, to thank for this change of my, total change of my life. *Woman, non-related-LD transplant following HD.*<br>**Worry:**<br>▪ Psychologically I would rather have had a dead donor. For the simple reason that you know, it's difficult to deal with taking a part of your daughter's body out which she may need in later years. She was 27 at the time. So… it was very difficult, very difficult. *Man, pre-emptive related-LD transplant.*<br>▪ She's an only child and she has 2 children of her own and (it's) such a sacrifice and if anything had gone wrong or even now if anything happens to her and her other kidney, … I worry about her. *Woman, related-LD transplant following HD.*<br>▪ You worry about the future you know. My (donor) she's very young, she's all of her life and that you know. *Man, pre-emptive related-LD transplant.*

Note. DD=deceased donor, LD=living donor, HD=haemodialysis, PD=peritoneal dialysis, APD=automated peritoneal dialysis.

For peer review only - http://bmjopen.bmj.com/site/about/guidelines.xhtml

For peer review only

For peer review only - http://bmjopen.bmj.com/site/about/guidelines.xhtml

Figure 1. CONSORT diagram.

Note. WL= wait-list group, DD = deceased donor, LD = living donor.

Figure 2. Interaction graphs showing differences in outcomes at recruitment and at one-year post-transplant/post-recruitment in those who remained on the waiting list (WL) for a kidney transplant (*n*=98), or those who were recruited as WL patients and subsequently received a DD kidney transplant (*n*=41), or a LD kidney transplant (*n*=26). Adjusted scores shown, controlling for age, previous RRT, previous experience of a transplant, education, car ownership, and history of mental health problems. **p*<0.05 WL vs LD/DD, ***p*<0.01 WL vs LD/DD, ****p*<0.001 WL vs LD/DD. WL= wait-list group, DD = deceased donor, LD = living donor.

Figure 3. Bar graph showing differences in satisfaction with current renal treatment compared with previous renal treatment at one-year post-transplant/ post-recruitment in those who remained on the waiting list for a kidney transplant, or those who received a DD or LD kidney transplant after recruitment. Adjusted scores shown, controlling for age, previous RRT, previous experience of a transplant, education, car ownership, history of mental health problems, and RTSQs scores at recruitment. WL= wait-list group, DD = deceased donor, LD = living donor.

For peer review only - http://bmjopen.bmj.com/site/about/guidelines.xhtml

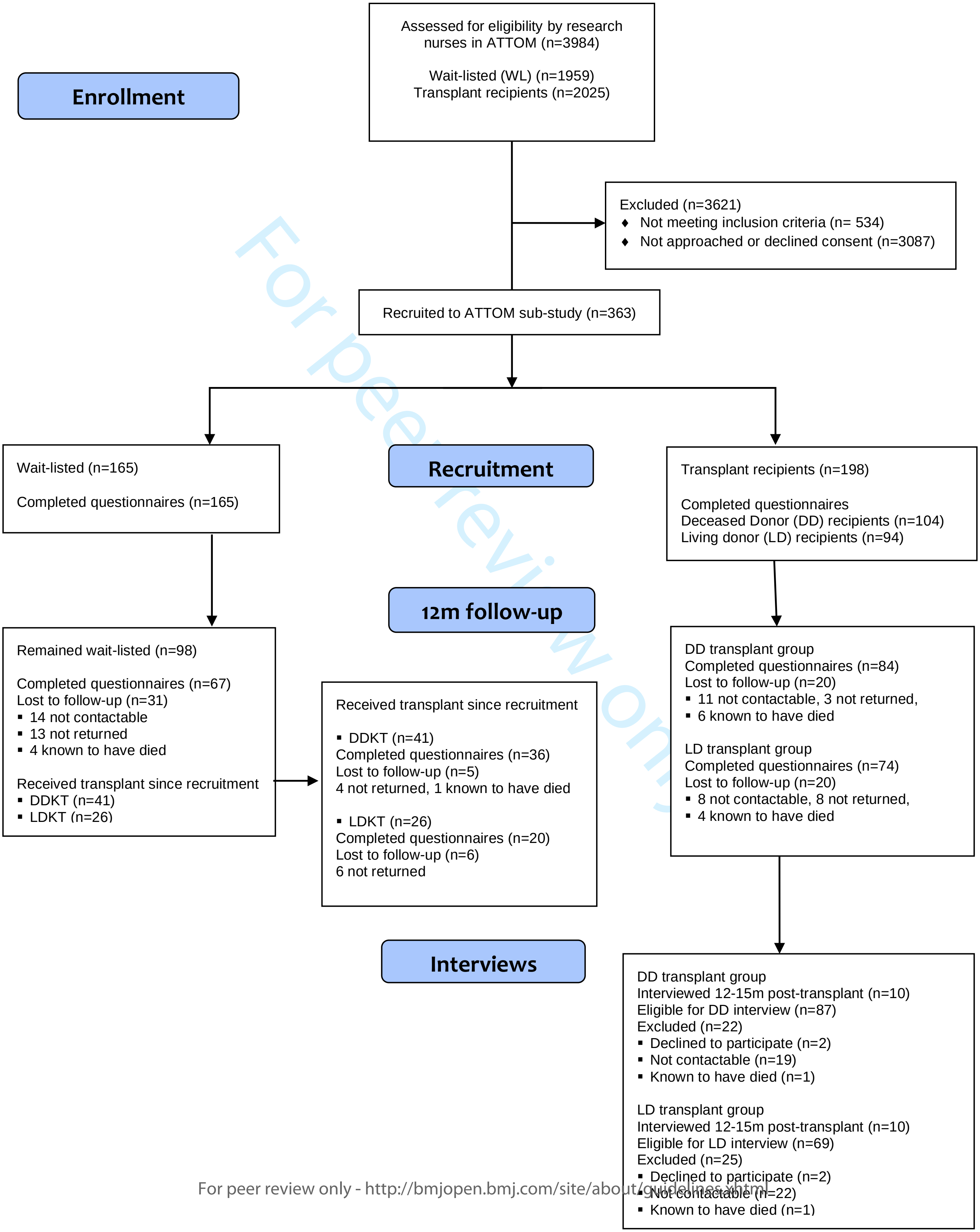

For peer review only - http://bmjopen.bmj.com/site/about/guidelines.xhtml

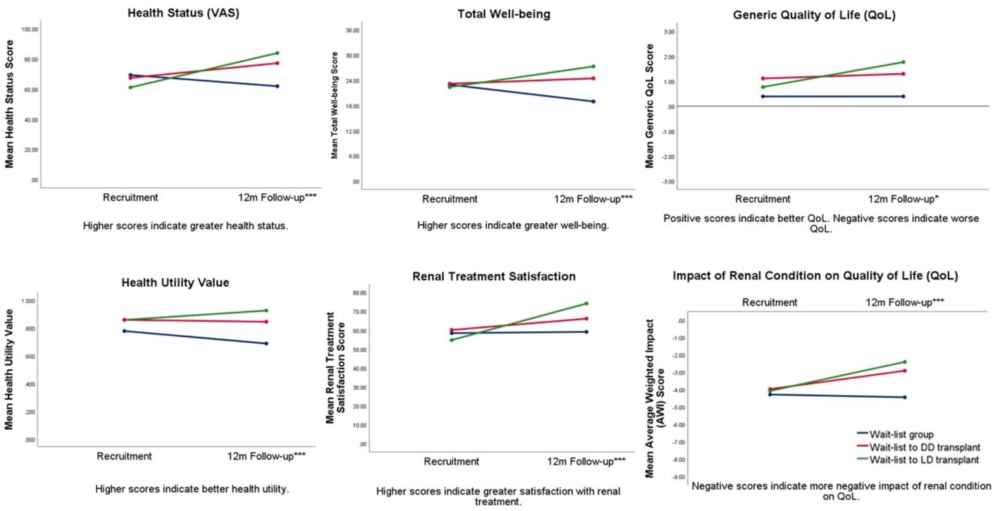

Figure 2. Interaction graphs showing differences in outcomes at recruitment and at one-year post-transplant/post-recruitment in those who remained on the waiting list (WL) for a kidney transplant (n=98), or those who were recruited as WL patients and subsequently received a DD kidney transplant (n=41), or a LD kidney transplant (n=26). Adjusted scores shown, controlling for age, previous RRT, previous experience of a transplant, education, car ownership, and history of mental health problems.

338x190mm (96 x 96 DPI)

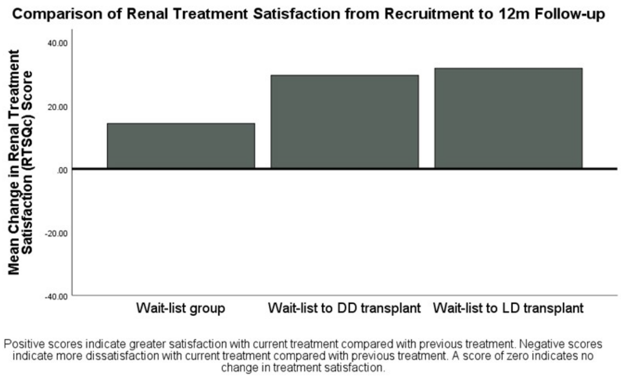

Figure 3. Bar graph showing differences in satisfaction with current renal treatment compared with previous renal treatment at one-year post-transplant/ post-recruitment in those who remained on the waiting list for a kidney transplant, or those who received a DD or LD kidney transplant after recruitment.

338x190mm (96 x 96 DPI)

Supplementary Data

Supplementary Data 1. Interview schedule for semi-structured interviews with LD and DD recipients.

The interview schedule included prompt questions, but each schedule was tailored to the individual and their questionnaire responses to the Renal Dependent Quality of Life (RDQoL) Questionnaire and the Renal Treatment Satisfaction Questionnaires (RTSQs).

HISTORY
Can you describe the process that you went through before you received a kidney transplant?

All patients:
- When did you first start having problems with your kidneys?
- How did your kidney problems develop?
- Were you placed on the transplant list? When?

For patients who received a DD transplant:
- How long were you waiting for a kidney?
- How did you feel about the time you spent waiting?
- Was the option of a living donor mentioned to you?

For patients who received an LD transplant:
- Who first suggested a living donor to you?
- How did you find information about living donation?
  - How satisfied were you with the information you were given?
- How did you bring the topic up with your family or friends?
  - Did you feel able to talk about it?
- Who donated a kidney to you?
  - How are they doing now?

QUALITY OF LIFE
Ok, so now I'd like to move on and talk to you about your quality of life.
In the questionnaires that you completed recently, you report that overall, your quality of life is excellent / very good / good / neither good nor bad / bad / very bad / extremely bad.

You said that your renal condition does / does not have an impact on your quality of life.
Can you give me an example of how (aspect of life) has been affected?
Other aspect(s) of your life that appeared to be most impacted by your renal condition was/were …………
Can you tell me in what ways your renal condition impacts on ………………….?

For patients with QoL changes over time:
In the questionnaires, you report that your quality of life has changed since you received a kidney transplant. Can you tell me why that is?

TREATMENT SATISFACTION
In terms of your treatment satisfaction, I can see from your questionnaires that you are very satisfied / satisfied / a little dissatisfied / dissatisfied / very dissatisfied with your current treatment. Can you tell me why?
I can see from your questionnaires that the aspect(s) of your treatment that you are most satisfied with was/were ………… Can you tell me why?
You were least satisfied with ………….. Can you tell me why?

For peer review only

Your treatment satisfaction did / did not change over time from 3 months after recruitment / transplant to 12 months after recruitment / transplant. Can you tell me why?

How might your satisfaction with treatment be improved?

For peer review only

Supplementary Data 2. Table 1. Summary of outcome measures

		No. of items	Range
Quality of life (ADDQoL and RDQoL)			
Generic QoL item	A single item assessing present QoL.	1	+3 (excellent) to -3 (extremely bad)
Renal Dependent Quality of Life (RDQoL)	Weighted-impact scores calculated by multiplying (a) impact of renal condition on aspect of life with (b) importance rating of aspect of life and summing all applicable items to give an average weighted-impact (AWI) score reflecting the impact of the renal condition on QoL.	21 (6 N/A options) *	-9 (most negative weighted impact) to +3 (most positive weighted impact)
Well-being (W-BQ12)			
Total well-being	12 items scored on 4-point scales from 3 (all the time) to 0 (not at all). Overall well-being score calculated by combining the scores from the 3 subscales (negative well-being, energy, positive well-being). Higher scores indicate greater well-being during the past few weeks.	12	36-0
Health status (EQ-5D-5L)			
Utility values	5 dimensions of health (today) rated on 5 levels converted to population preference values using the new value set for England.[18] The higher the score, the greater the preference utility.	5	+1.00 (perfect health) to -0.281 (worse than death)
EQ-VAS ratings	Self-reported health (today) on visual analogue scale from 100 (best health you can imagine) to 0 (worst health you can imagine).	1	100-0
Treatment satisfaction (RTSQs/c)			
Renal Treatment Satisfaction Questionnaire (RTSQ) status version (RTSQs)	Items assess satisfaction with current renal treatment on 7-point scales from 6 (e.g. very satisfied) to 0 (e.g. very dissatisfied). Items are summed to give a total satisfaction score. The higher the score, the greater the satisfaction with renal treatment.	13	78-0
RTSQ change version (RTSQc)	Items assess satisfaction with current renal treatment compared with satisfaction with previous renal treatment, from +3 (e.g. much more satisfied now) to -3 (e.g. much less satisfied now). A score of 0 indicates no change in satisfaction. Items are summed to give a total change in satisfaction score.	13	+39 (much more satisfied now) to 39 (much less satisfied now)

*after preliminary psychometric analyses, only 17 of the items (including 3 with not applicable options) were included in the Average Weighted Impact (AWI) score (see Appendix 2). The range of AWI scores remains the same.

For peer review only

Supplementary Data 3. Psychometric properties of the RDQoL and RTSQs/c

The Renal Dependent Quality of Life (RDQoL) questionnaire was designed to examine the impact of renal conditions on QoL.[1] Following the template of the Audit of Diabetes-Dependent QoL (ADDQoL),[2,3] the RDQoL contains two overview items and a series of domain-specific items covering different aspects of life commonly impacted by a renal condition and its treatment. Each domain-specific item is worded *'If did not have a renal condition my…would be'*, followed by a rating scale for the impact of renal condition on that domain of life from -3 (e.g. very much better) to +1 (e.g. worse), and a rating of the importance of the domain for QoL from very important (+3) to not at all important (0). The impact and importance scores are multiplied to give a weighted impact score for each item, ranging from -9 (most negative weighted impact on QoL) to +3 (most positive weighted impact). Some of the items include preliminary questions to determine if the domain is applicable to the individual (e.g. work). Each of the applicable weighted impact scores are summed and the total is divided by the number of applicable items to give an average weighted impact (AWI) score, also ranging from -9 to +3.

The Renal Treatment Satisfaction Questionnaire (RTSQ) is a measure of satisfaction with renal treatment. Following the templates of the Diabetes Treatment Satisfaction Questionnaire (DTSQ) [4-6] there are two versions: the status version (RTSQs)[7] and the change version (RTSQc). The RTSQs assesses level of satisfaction with current renal treatment. The 13 items are summed to give an overall treatment satisfaction score, with higher scores indicating greater satisfaction (range=78-0). The RTSQc was developed to overcome the ceiling effects sometimes found with the status version. Using similar item stems as the RTSQs but different response options, the RTSQc asks participants to compare satisfaction with their current treatment with satisfaction with their previous treatment (range +39 e.g. much more satisfied to -39 much less satisfied). Barendse et al[7] conducted analyses on the original 11-item RTSQs, and found that it had acceptable reliability. Although a forced one-factor structure was acceptable for all patients, a two-factor structure was found for transplant recipients, and a three-factor structure for non-transplant patients.[6] The 13-item version of the RTSQs has additional

For peer review only

items related to satisfaction with the side effects of treatment, and satisfaction with the demands of treatment. No psychometric analyses have been published using the 13-item version, and none have been conducted with the RTSQc. Analyses, therefore, were conducted to test the psychometric properties of these measures in this sample.

In ATTOM, the number of participants in each group recruited were as follows; 104 DD recipients, 94 LD recipients, 98 patients wait-listed for a transplant, 136 participants receiving PD or HD, and 16 patients whose transplant failed during ATTOM. Participants were invited to complete the measures at 12m post-transplant or post-recruitment. There was a possibility that differences between the groups might create artefactual correlations in the combined sample, if the subgroup mean scores differed systematically across items. The possibility of artefactual correlations was therefore investigated by converting the scores on each measure to standardised z scores within each subgroup before combining the sub-sets of scores and conducting the factor analyses. Such standardisation renders group means identical and thereby removes the possibility of correlations caused by subgroup differences. Forced one-factor solutions on the raw scores, and then on the Z scores, produced two sets of factor loadings that were then compared using correlation and regression cross-check analyses.

The crosscheck analyses tested whether the transplant groups (DD and LD recipients) and non-transplant groups could be combined for these analyses. The non-transplant groups included dialysis and pre-dialysis patients including those wait-listed for a kidney transplant, and those whose transplants failed during ATTOM. For ease of analysis, these were grouped into those on HD, PD, or pre-dialysis. Participants with missing data on any items were excluded from the analyses. The transplant groups could be combined for analyses for the RDQoL, RTSQs and RTSQc. The non-transplant groups (on either HD, PD or pre-dialysis) could be combined for the RDQoL and RTSQs, whilst only the HD and PD groups could be combined for the RTSQc. Those not yet on dialysis formed a much smaller sub-group (*n*=15), so were not included in separate analyses.

For peer review only

RDQoL

Factor structure

Analyses were conducted to confirm the factor structure of the RDQoL, RTSQs, and RTSQc. Parallel analyses were conducted to identify how many factors are acceptable to retain in the structure of each measure. Principal Axis Factoring (PAF) was then used to examine the variance and the item loadings unto these factors. A Kaiser-Meyer-Olkin Measure of Sampling Adequacy (KMO) value of 0.60 or above, and correlation coefficients of 0.30 and above between each item indicates that the analysis is appropriate and the items should be retained. An item loading above 0·40 is considered acceptable. See Tables 5 and 6 for a summary of the descriptive statistics, reliability analysis, and factor structure of the RDQoL for transplant and non-transplant groups. Parallel analysis indicated that a one-factor solution was acceptable for both the transplant and non-transplant groups.

Transplant (LD and DD) group: Principal Axis Factoring (PAF) with a forced one-factor solution led to 48·04% of the variance being explained, with item loadings from 0.266 to 0.855 (KMO=0.92, $\chi^2_{(210)}$=1738.60, p<0.001). The spiritual/religious life item had the lowest item loading of 0.266, and correlated with only one other item above 0.30, so was removed, and the analysis re-run. This led to 49·97% of the variance being explained, with more acceptable loadings ranging from 0.417 to 0.856 (KMO=0.93, $\chi^2_{(210)}$=1715.451, p<0.001). Item 19 (fuss/worry), although displaying an acceptable factor loading (0.417), correlated with few other items above 0.30.

Non-transplant group: Principal Axis Factoring with a forced one-factor solution led to 38.95% of the variance being explained, with item loadings from 0.250 to 0.730 (KMO=0.92, $\chi^2_{(210)}$=1431.71, p<0.001). Item 2 (work, 0.250), item 4 (holidays 0.331), and item 14 (religious/spiritual life 0.349) had item loadings below 0.40. Work was only correlated above 0.30 with one other item, whilst holidays and spiritual/religious life were each correlated with four other items above 0.30. These items were removed, and the analysis re-run. This led to 43.70% of the variance being explained, with item loadings from 0.552 to 0.740 (KMO=0.93, $\chi^2_{(153)}$=1308.43, p<0.001).

For peer review only

Supplementary Table 2. Descriptive statistics, reliability analysis, and forced one-factor solution Principal Axis Factoring (PAF) on the RDQoL weighted impact scores in the transplant recipients (with deceased and living donor kidneys; *n* = 137).

	Missing N	**Not applicable**	**Mean**	**Median**	***SD***	**Corrected Item-total correlation**	**Cronbach's alpha if item deleted***	**PAF Item Loadings**
**1. Leisure activities**	1	-	-2.72	-2.00	2.68	0.717	0.940	0.741
**2. Work**	3	37	-2.46	0.00	3.18	0.610	0.942	0.617
**3. Local/long distance journeys**	1	-	-1.79	-1.00	2.41	0.612	0.942	0.630
**4. Holidays**	3	9	-2.83	-2.00	2.96	0.716	0.940	0.735
**5. Physical ability**	2	-	-3.06	-3.00	2.80	0.783	0.939	0.811
**6. Family**	1	1	-2.42	-1.50	2.89	0.714	0.940	0.743
**7. Friendships/social life**	0	-	-2.34	-1.50	2.88	0.789	0.939	0.815
**8. Close personal relationship**	3	14	-2.37	0.00	3.10	0.713	0.940	0.746
**9. Sex life**	2	16	-2.61	-2.00	2.99	0.723	0.940	0.751
**10. Physical appearance**	3	-	-2.37	-2.00	2.60	0.540	0.943	0.561
**11. Self-confidence**	1	-	-2.25	-2.00	2.74	0.783	0.939	0.813
**12. Motivation**	4	-	-2.26	-2.00	2.86	0.825	0.938	0.855
**13. People react to me**	1	-	-0.84	0.00	1.78	0.607	0.942	0.633
**14. Spiritual/religious life**	0	75	-0.31	0.00	1.75	-	-	0.266
**15. Feelings about the future**	0	-	-3.42	-2.00	3.34	0.576	0.943	0.588
**16. Financial situation**	1	-	-2.61	-2.00	3.23	0.686	0.941	0.696
**17. Living conditions**	1	-	-1.41	0.00	2.40	0.659	0.941	0.685
**18. Dependence on others**	2	-	-2.40	-1.00	3.06	0.543	0.943	0.548
**19. People fuss/worry**	0	-	-3.20	-2.00	3.05	0.417	0.945	0.427
**20. Freedom to eat**	0	-	-1.76	-1.00	2.45	0.614	0.942	0.635
**21. Freedom to drink**	0	-	-1.69	-1.00	2.51	0.603	0.942	0.614

*Total Cronbach's alpha = 0·944.

Reliability analyses

Cronbach's alpha (α) was calculated as a test of internal consistency; how closely related the items in the measure are as a group. Scores range from 0 to 1, with a score above 0.70 commonly being used as a threshold for acceptable reliability for group comparison with 0.80 providing a more stringent threshold. Analyses are repeated, removing one item at a time to calculate how many missing items a measure can tolerate, whilst still retaining an acceptable alpha coefficient. Even if the alpha

For peer review only

coefficient is above the threshold, no more than 50% of the items in a scale or subscale are allowed to be missing, because to do so is likely to produce too much distortion of the construct measured by the scale.

Transplant (LD and DD) group: Analyses conducted using all items except item 14 (spiritual/religious life) revealed that the RDQoL has excellent reliability (Cronbach's α=0.944). The removal of item 19 (fuss or worry) would improve the alpha coefficient from 0.944 to 0.945. The fuss and worry item also had the lowest item-total correlations (0·417), and the lowest item loadings (0.427), suggesting that its removal could improve the factor structure and reliability of the RDQoL.

Non-transplant group: Reliability analyses revealed that, not including item 2 (work) and 14 (spiritual/religious life), the RDQoL has excellent reliability (Cronbach's α=0.920; Table 6). The removal of item 4 (holidays) would improve the alpha coefficient from 0.920 to 0.922. The item had a low factor loading (0.331) and low item-total correlation (r = 0.326).

Not including those items that have a non-applicable option, reliability analyses indicated that the questionnaire (14 items) could tolerate up to seven missing items and still retain a Cronbach's α coefficient above 0.70 for the transplant group (α = 0.786) and non-transplant group (α = 0.792).

For peer review only

Supplementary Table 3. Descriptive statistics, reliability analysis, and forced one-factor solution Principal Axis Factoring (PAF) on the RDQoL weighted impact scores in the non-transplant groups (*n*= 172).

	Missing N	Not applicable	Mean	Median	*SD*	Corrected Item-total correlation	Cronbach's alpha if item deleted*	PAF Item Loadings
**1. Leisure activities**	3	-	-4.84	-4.00	2.88	0.587	0.916	0.604
**2. Work**	2	85	-2.85	0.00	3.55	-	-	0.250
**3. Local/long distance journeys**	5	-	-4.34	-4.00	3.27	0.545	0.917	0.556
**4. Holidays**	2	30	-4.94	-6.00	3.42	0.326	0.922	0.331
**5. Physical ability**	1	-	-5.64	-6.00	2.81	0.576	0.916	0.582
**6. Family**	3	1	-5.14	-6.00	3.42	0.619	0.915	0.650
**7. Friendships and social life**	1	-	-4.59	-4.00	3.22	0.676	0.914	0.695
**8. Close personal relationship**	3	21	-4.13	-4.00	3.57	0.657	0.914	0.698
**9. Sex life**	1	35	-3.69	-4.00	3.24	0.611	0.915	0.638
**10. Physical appearance**	0	-	-2.92	-2.00	2.99	0.650	0.942	0.688
**11. Self-confidence**	1	-	-3.40	-2.00	3.14	0.659	0.914	0.702
**12. Motivation**	0	-	-4.20	-4.00	3.05	0.696	0.913	0.730
**13. People react to me**	2	-	-1.45	0.00	2.22	0.542	0.917	0.584
**14. Spiritual/religious life**	2	104	-0.74	0.00	2.03	-	-	0.349
**15. Feelings about the future**	1	-	-5.20	-6.00	3.35	0.682	0.913	0.701
**16. Financial situation**	1	-	-3.79	-4.00	3.50	0.490	0.918	0.519
**17. Living conditions**	2	-	-3.09	-2.00	3.26	0.609	0.915	0.665
**18. Dependence on others**	3	-	-3.95	-4.00	3.27	0.660	0.914	0.695
**19. People fuss/worry**	1	-	-3.64	-4.00	3.16	0.564	0.916	0.582
**20. Freedom to eat**	0	-	-4.56	-4.00	3.24	0.557	0.916	0.570
**21. Freedom to drink**	0	-	-4.84	-4.00	3.35	0.531	0.917	0.543

*Total Cronbach's alpha = 0.920.

These findings suggest that item 14 (spiritual/religious life) should be removed for both groups, as the majority of people reported either not having a spiritual or religious life (54.4% transplant group, 60.4% non-transplant group), or reported that it was not impacted by their renal condition (70.8% transplant group, 52.8% non-transplant group). Removing item 19 (fuss or worry) would improve the psychometrics of the questionnaire for transplant recipients. For the non-transplant group, treating item 2 (work) and item 4 (holidays) as separate items to the AWI scores may be indicated. Although the holidays item was relevant for the majority of the patients (93.6% transplant group, 73.8% non-transplant group), the analyses indicate that its removal from the overall AWI scores would improve the factor structure and reliability for the non-transplant group.

For peer review only

Supplementary Table 4. Reliability analysis and forced one-factor solution Principal Axis Factoring (PAF) on 17 items of the RDQoL weighted impact scores for the transplant group (*n*=142) and non-transplant group (*n*=158).

	Transplant group			Non-transplant group		
	**Corrected Item-total correlation**	**Cronbach's alpha if item deleted***	**PAF Item Loadings**	**Corrected Item-total correlation**	**Cronbach's alpha if item deleted***	**PAF Item Loadings**
**1. Leisure activities**	0.708	0.934	0.729	0.575	0.915	0.596
**3. Local/long distance journeys**	0.592	0.937	0.608	0.529	0.916	0.550
**5. Physical ability**	0.784	0.932	0.812	0.547	0.915	0.565
**6. Family**	0.727	0.934	0.753	0.622	0.913	0.658
**7. Friendships/social life**	0.761	0.933	0.791	0.666	0.912	0.696
**8. Close personal relationship**	0.718	0.934	0.747	0.658	0.912	0.695
**9. Sex life**	0.727	0.934	0.751	0.622	0.913	0.650
**10. Physical appearance**	0.557	0.937	0.577	0.645	0.913	0.674
**11. Self-confidence**	0.786	0.932	0.810	0.673	0.912	0.714
**12. Motivation**	0.817	0.932	0.848	0.709	0.911	0.744
**13. People react to me**	0.627	0.937	0.650	0.553	0.916	0.585
**15. Feelings about the future**	0.570	0.938	0.584	0.672	0.912	0.699
**16. Financial situation**	0.686	0.935	0.701	0.497	0.917	0.516
**17. Living conditions**	0.662	0.935	0.689	0.623	0.913	0.651
**18. Dependence on others**	0.496	0.939	0.512	0.637	0.913	0.668
**20. Freedom to eat**	0.611	0.936	0.629	0.553	0.915	0.572
**21. Freedom to drink**	0.587	0.937	0.600	0.530	0.916	0.548

*Transplant group total Cronbach's alpha 0.939; non-transplant group total Cronbach's alpha 0.918

Running the analyses again with the removal of items 2 (work), 4 (holidays), 14 (spiritual/religious life), and 19 (fuss/worry) for both groups indicated an acceptable factor structure and good reliability (Table 7). Therefore, to be able to calculate the AWI scores for all participants, without negatively impacting the psychometrics of the questionnaires, it was decided that item 2 (work) and 4 (holidays) items would be considered as separate items, whilst items 14 (spiritual/religious life) and 19 (fuss/worry) would be removed.

For peer review only

RTSQs

Factor structure: Parallel analysis indicated that a one-factor solution was optimal for both the transplant and non-transplant groups (see Table 8). For the transplant group, Principal Axis Factoring with a forced one-factor solution explained 50.65% of the variance, with item loadings from 0.521 to 0.814 (KMO=0.88, $\chi^2_{(78)}$=1197.74, p<0.001). Similar results were found with the non-transplant group, with 50.80% of the variance being explained, with item loadings from 0.452 to 0.848 (KMO=0.91, $\chi^2_{(78)}$=1321.69, p<0.001).

Reliability analyses: Previous research on the 11-item RTSQs[24] indicated that a forced one-factor solution was acceptable and up to 5 items could be tolerated whilst retaining an alpha coefficient of 0.79. Reliability analyses on the 13-item RTSQs revealed that it has excellent reliability for the transplant (α=0.905) and non-transplant groups (α=0.918). The removal of item 3 (side effects of treatment) would improve the alpha coefficient for both groups, but the item was correlated with the majority of the other items above 0.30, indicating that it should be included. All other items contributed to the reliability of the measure. It was possible to remove up to five items in this 13-item measure and retain a Cronbach's α coefficient above 0.80 for the transplant (α = 0.806) and non-transplant groups (α = 0.832). These findings confirmed that optimal scoring of the RTSQs includes all 13 items.

For peer review only

Supplementary Table 5. Descriptive statistics, reliability analysis, and forced one-factor solution Principal Axis Factoring (PAF) on the RTSQs items, completed by the transplant and non-transplant groups.

	Missing *N*	Mean	Median	*SD*	Corrected Item-total correlation	Cronbach's alpha if item deleted*	PAF Item Loadings
**Transplant group (*N* = 156)**							
**1. Overall satisfaction score**	0	5.58	6.00	0.78	0.707	0.896	0.740
**2. Control of renal condition**	0	5.48	6.00	0.83	0.668	0.897	0.721
**3. Side effects**	1	4.44	5.00	1.51	0.547	0.908	0.558
**4. Demands of treatment**	0	5.23	5.00	0.93	0.732	0.893	0.756
**5. Convenience of treatment**	0	5.23	6.00	1.08	0.730	0.893	0.763
**6. Flexibility of treatment**	0	5.22	6.00	1.16	0.518	0.904	0.559
**7. Freedom on treatment**	0	5.52	6.00	0.81	0.701	0.896	0.745
**8. Understanding of treatment**	0	5.63	6.00	0.69	0.526	0.902	0.569
**9. Time taken by treatment**	0	5.43	6.00	0.93	0.755	0.892	0.814
**10. Discomfort or pain**	1	5.32	6.00	1.15	0.522	0.904	0.521
**11. Treatment fits in with lifestyle**	0	5.41	6.00	1.02	0.700	0.894	0.745
**12. Recommend treatment to others**	1	5.75	6.00	0.70	0.607	0.900	0.634
**13. Satisfaction to continue treatment**	1	5.71	6.00	0.70	0.647	0.898	0.684
**Non-transplant group (*N* = 190)**							
**1. Overall satisfaction score**	1	4.99	5.00	1.09	0.522	0.917	0.546
**2. Control of renal condition**	0	4.94	5.00	1.03	0.552	0.916	0.576
**3. Side effects**	0	4.04	4.00	1.51	0.606	0.914	0.635
**4. Demands of treatment**	1	3.97	4.00	1.62	0.786	0.906	0.821
**5. Convenience of treatment**	0	4.19	5.00	1.64	0.812	0.905	0.848
**6. Flexibility of treatment**	0	4.30	5.00	1.71	0.730	0.909	0.764
**7. Freedom on treatment**	0	3.78	4.00	1.78	0.732	0.909	0.764
**8. Understanding of treatment**	0	5.16	5.00	1.05	0.430	0.919	0.452
**9. Time taken by treatment**	0	4.03	4.00	1.77	0.763	0.907	0.795
**10. Discomfort or pain**	1	4.39	5.00	1.46	0.557	0.915	0.579
**11. Treatment fits in with lifestyle**	1	3.77	4.00	1.65	0.699	0.910	0.726
**12. Recommend treatment to others**	3	5.05	6.00	1.31	0.619	0.913	0.642
**13. Satisfaction to continue treatment**	1	4.68	5.00	1.62	0.619	0.913	0.646

*Transplant group total Cronbach's alpha = 0.905. Non-transplant total Cronbach's alpha =0.918

For peer review only

RTSQc

Factor structure: See Table 9 for summaries of descriptive statistics, reliability analyses, and the factor structure of the RTSQc. Parallel analysis indicated that a one-factor solution was acceptable for the transplant and non-transplant groups. Principal Axis Factoring with a forced one-factor solution explained 56.22% of the variance with item loadings from 0.466 to 0.832 (KMO=0.90, $\chi^2_{(78)}$ =1353.40, p<0.001). Similar results were found for the non-transplant groups; 67.87% of the variance was explained, with item loadings from 0.601 to 0.935 (KMO=0.93, $\chi^2_{(78)}$ =1929.77, p<0.001).

Reliability analyses: Analyses revealed that the RTSQc has excellent reliability for the transplant (α=0.930) and non-transplant group (α=0.960). The removal of item 8 (understanding of treatment) would improve the alpha coefficient slightly for both groups. Despite this, item was 8 correlated with other items above 0.30, and the item loadings were acceptable, indicating that it should be included. Reliability analyses indicated that in this 13-item measure, it was possible to remove up to six items and retain a Cronbach's α coefficient of 0.826 for the transplant group and 0.906 for the non-transplant group.

In line with previous research, these findings suggest that the RTSQs and RTSQc can be considered to have a one-factor structure across various treatment groups and time points. Although the exclusion of various items is possible for the RTSQs and RTSQc, all items contribute to the factor structure, and the reliability is excellent using all 13 items.

For peer review only

Supplementary Table 6. Descriptive statistics, reliability analysis, and forced one-factor solution Principal Axis Factoring (PAF) on the RTSQc items, completed by transplant (DD and LD) and non-transplant (HD and PD) groups.

	Missing *N*	Mean	Median	*SD*	Corrected Item-total correlation	Cronbach's alpha if item deleted*	PAF Item Loadings for 1 factor solution
**LD and DD groups (*n*=150)**							
**1. Overall satisfaction score**	3	2.42	3.00	1.07	0.702	0.924	0.723
**2. Control of renal condition**	3	2.50	3.00	1.03	0.720	0.923	0.748
**3. Side effects**	3	1.69	2.00	1.54	0.599	0.930	0.616
**4. Demands of treatment**	4	2.32	3.00	1.08	0.800	0.920	0.833
**5. Convenience of treatment**	4	2.27	3.00	1.26	0.783	0.920	0.824
**6. Flexibility of treatment**	4	2.26	3.00	1.21	0.678	0.924	0.714
**7. Freedom on treatment**	4	2.33	3.00	1.09	0.796	0.921	0.832
**8. Understanding of treatment**	5	2.17	3.00	1.15	0.449	0.932	0.486
**9. Time taken by treatment**	5	2.37	3.00	1.07	0.739	0.922	0.764
**10. Discomfort or pain**	8	2.18	3.00	1.24	0.616	0.927	0.628
**11. Treatment fits in with lifestyle**	7	2.32	3.00	1.16	0.760	0.922	0.803
**12. Recommend treatment to others**	6	2.58	3.00	0.99	0.564	0.928	0.581
**13. Satisfaction to continue treatment**	7	2.50	3.00	1.08	0.787	0.921	0.814
**HD and PD groups (*n*=170)**							
**1. Overall satisfaction score**	0	1.30	2.00	1.73	0.788	0.957	0.802
**2. Control of renal condition**	0	1.48	2.00	1.60	0.676	0.960	0.686
**3. Side effects**	0	0.89	1.00	1.77	0.777	0.957	0.791
**4. Demands of treatment**	0	0.96	1.00	1.72	0.888	0.955	0.910
**5. Convenience of treatment**	0	0.98	1.00	1.82	0.912	0.954	0.935
**6. Flexibility of treatment**	0	0.91	1.00	1.68	0.832	0.956	0.852
**7. Freedom on treatment**	0	0.62	1.00	1.86	0.941	0.956	0.862
**8. Understanding of treatment**	0	1.76	2.00	1.27	0.592	0.961	0.601
**9. Time taken by treatment**	0	0.89	2.00	1.80	0.862	0.955	0.884
**10. Discomfort or pain**	0	0.91	2.00	1.57	0.733	0.958	0.746
**11. Treatment fits in with lifestyle**	0	0.73	2.00	1.73	0.851	0.955	0.871
**12. Recommend treatment to others**	0	1.69	3.00	1.59	0.737	0.958	0.748
**13. Satisfaction to continue treatment**	0	1.39	1.00	1.73	0.748	0.958	0.761

*LD and DD groups total Cronbach's alpha = 0.930. HD and PD groups total Cronbach's alpha = 0.960. LD=living donor; DD= deceased donor; HD=haemodialysis' PD= peritoneal dialysis.

For peer review only

References

1. Bradley C. Design of a Renal-Dependent Individualized Quality of Life Questionnaire. *Adv Perit Dial* 1997; 13: 116-120.
2. Bradley C, Todd C, Gorton T, et al. The development of an individualised measure of perceived impact of diabetes on quality of life: The ADDQoL. *Qual Life Res* 1999; 8(1-2): 79-91.doi:10.1023/A:1026485130100.
3. Wee HL, Tan CE, Goh SY, Li SC. Usefulness of the Audit of Diabetes-Dependent Quality-of-Life (ADDQoL) Questionnaire in patients with diabetes in a multi-ethnic Asian country. *Pharmacoeconomics* 2006; 24(7): 673-682.
4. Bradley C, Lewis KS. Measures of psychological well-being and treatment satisfaction developed from the responses of people with tablet-treated diabetes. Diabetic Med 1990; 7, 445-451.
5. Bradley C. The Diabetes Treatment Satisfaction Questionnaire: DTSQ. In Bradley C. (Ed). Handbook of Psychology and Diabetes: A guide to psychological measurement in diabetes research and practice. Chur Switzerland: Harwood Academic Publishers, 1994; 111-132.
6. Howorka K, Pumprla J, Schlusche C, et al. Dealing with ceiling baseline treatment satisfaction level in patients with diabetes under flexible, functional insulin treatment: Assessment of improvements in treatment satisfaction with a new insulin analogue. *Qual Life Res* 2000; 9: 915-930.
7. Barendse SM, Speight J, Bradley C. The Renal Treatment Satisfaction Questionnaire (RTSQ): A measure of satisfaction with treatment for chronic kidney failure. *Am J Kidney Dis* 2005; 45: 572-579.

For peer review only

STROBE 2007 (v4) checklist of items to be included in reports of observational studies in epidemiology*
Checklist for cohort, case-control, and cross-sectional studies (combined)

Section/Topic	Item #	Recommendation	Reported on page #
**Title and abstract**	1	(*a*) Indicate the study's design with a commonly used term in the title or the abstract	1 (line 1-4)
		(*b*) Provide in the abstract an informative and balanced summary of what was done and what was found	3 (line 2) - 4 (line 2)
**Introduction**			
Background/rationale	2	Explain the scientific background and rationale for the investigation being reported	5 (line 1) – 7 (line 17)
Objectives	3	State specific objectives, including any pre-specified hypotheses	7 (line 18) – 8 (line 3)
**Methods**			
Study design	4	Present key elements of study design early in the paper	8 (line 6 -15)
Setting	5	Describe the setting, locations, and relevant dates, including periods of recruitment, exposure, follow-up, and data collection	8 (line 6) – 10 (line 2)
Participants	6	(*a*) *Cohort study*—Give the eligibility criteria, and the sources and methods of selection of participants. Describe methods of follow-up<br>*Case-control study*—Give the eligibility criteria, and the sources and methods of case ascertainment and control selection. Give the rationale for the choice of cases and controls<br>*Cross-sectional study*—Give the eligibility criteria, and the sources and methods of selection of participants	8 (line 11) – 10 (line 2)
		(*b*) *Cohort study*—For matched studies, give matching criteria and number of exposed and unexposed<br>*Case-control study*—For matched studies, give matching criteria and the number of controls per case	8 (line 11) – 10 (line 2)
Variables	7	Clearly define all outcomes, exposures, predictors, potential confounders, and effect modifiers. Give diagnostic criteria, if applicable	10 (line 6) – 12 (line 18)
Data sources/ measurement	8*	For each variable of interest, give sources of data and details of methods of assessment (measurement). Describe comparability of assessment methods if there is more than one group	8 (line 20) – 9 (line 15)
Bias	9	Describe any efforts to address potential sources of bias	12 (line 18) - 13 (line 3)
Study size	10	Explain how the study size was arrived at	9 (lines 15-20)
Quantitative variables	11	Explain how quantitative variables were handled in the analyses. If applicable, describe which groupings were chosen and why	12 (line 18) – 13 (line 18)
Statistical methods	12	(*a*) Describe all statistical methods, including those used to control for confounding	12 (line 18) – 13 (line 18); 15 (lines 6-26).
		(*b*) Describe any methods used to examine subgroups and interactions	12 (line 26) – 13 (line 1)

For peer review only

		(*c*) Explain how missing data were addressed	14 (lines 23-24)
		(*d*) *Cohort study*—If applicable, explain how loss to follow-up was addressed<br>*Case-control study*—If applicable, explain how matching of cases and controls was addressed<br>*Cross-sectional study*—If applicable, describe analytical methods taking account of sampling strategy	9 (lines 15-18)
		(*e*) Describe any sensitivity analyses	n/a
**Results**			
Participants	13*	(a) Report numbers of individuals at each stage of study—eg numbers potentially eligible, examined for eligibility, confirmed eligible, included in the study, completing follow-up, and analysed	Figure 1.
		(b) Give reasons for non-participation at each stage	Figure 1.
		(c) Consider use of a flow diagram	Figure 1.
Descriptive data	14*	(a) Give characteristics of study participants (eg demographic, clinical, social) and information on exposures and potential confounders	Table 1. 14 (lines 1-24)
		(b) Indicate number of participants with missing data for each variable of interest	14 (lines 23-24)
		(c) *Cohort study*—Summarise follow-up time (eg, average and total amount)	Table 1. 14 (lines 1-24)
Outcome data	15*	*Cohort study*—Report numbers of outcome events or summary measures over time	15 (line 6) – 17 (line 6), Tables 2-4
		Case-control study—Report numbers in each exposure category, or summary measures of exposure	15 (line 6) – 17 (line 6), Tables 2-4
		Cross-sectional study—Report numbers of outcome events or summary measures	15 (line 6) – 17 (line 6), Tables 2-4
Main results	16	(*a*) Give unadjusted estimates and, if applicable, confounder-adjusted estimates and their precision (eg, 95% confidence interval). Make clear which confounders were adjusted for and why they were included	15 (line 6) – 17 (line 6), Tables 2-4
		(*b*) Report category boundaries when continuous variables were categorized	n/a
		(*c*) If relevant, consider translating estimates of relative risk into absolute risk for a meaningful time period	n/a
Other analyses	17	Report other analyses done—eg analyses of subgroups and interactions, and sensitivity analyses	n/a
**Discussion**			
Key results	18	Summarise key results with reference to study objectives	18 (lines 7-22); 19 (line 15) – 20 (line 2)
Limitations	19	Discuss limitations of the study, taking into account sources of potential bias or imprecision. Discuss both direction and magnitude of any potential bias	19 (line 3) - 20 (line 3)
Interpretation	20	Give a cautious overall interpretation of results considering objectives, limitations, multiplicity of analyses, results from similar studies, and other relevant evidence	21 (line 13) – 22 (line

For peer review only

			3)
Generalisability	21	Discuss the generalisability (external validity) of the study results	21 (line 13) – 22 (line 3)
**Other information**			
Funding	22	Give the source of funding and the role of the funders for the present study and, if applicable, for the original study on which the present article is based	39 (line 24) – 40 (line 2)

*Give information separately for cases and controls in case-control studies and, if applicable, for exposed and unexposed groups in cohort and cross-sectional studies.

Note: An Explanation and Elaboration article discusses each checklist item and gives methodological background and published examples of transparent reporting. The STROBE checklist is best used in conjunction with this article (freely available on the Web sites of PLoS Medicine at http://www.plosmedicine.org/, Annals of Internal Medicine at http://www.annals.org/, and Epidemiology at http://www.epidem.com/). Information on the STROBE Initiative is available at www.strobe-statement.org.

For peer review only
